# Supplementary material for: Explainable Thyroid Cancer Diagnosis Through Two-Level Machine Learning Optimization with an Improved Naked Mole-Rat Algorithm
Source: Cancers (Basel). 2024 Dec 10;16(24):4128. doi: 10.3390/cancers16244128 (PMC11674737; doi:10.3390/cancers16244128)
Supplement: Supplementary file 1 [file cancers-16-04128-s001.zip › cancers-3312311-supplementary.pdf]

Supplementary materials for „Explainable Thyroid Cancer Diagnosis Through Two-Level Machine Learning Optimization with an Improved Naked Mole-Rat Algorithm"

The supplementary materials contain detailed results (confusion matrices and ROC curves) for all classification models used in the three scenarios of the conducted experiments.

1. Classifiers with default parameter values 4
   1.1. Decision Tree 4
   1.2. Extra Tree 5
   1.3. Extra Trees 6
   1.4. Gradient Boosting 7
   1.5. KNN 8
   1.6. LightGBM 9
   1.7. Logistic Regression 10
   1.8. NuSVM 11
   1.9. Random Forest 12
   1.10. XgBoost 13
2. Optimization of classifier parameters using the Naked Mole-Rat Algorithm 14
   2.1. Decision Tree 14
   2.2. Extra Tree 15
   2.3. Extra Trees 16
   2.4. Gradient Boosting 17
   2.5. KNN 18
   2.6. LightGBM 19
   2.7. Logistic Regression 20
   2.8. NuSVM 21
   2.9. Random Forest 22
   2.10. XgBoost 23
3. Optimization of classifier parameters and feature selection using the Naked Mole-Rat Algorithm 24
   3.1. Decision Tree 24
   3.2. Extra tree 25

3.3. Extra Trees 26
3.4. Gradient Boosting 27
3.5. KNN 28
3.6. LightGBM 29
3.7. Logistic Regression 30
3.8. NuSVM 31
3.9. Random Forest 32
3.10. XgBoost 33
4. Optimization of classifier parameters and feature selection using the Naked Mole-Rat Algorithm with oversampling techniques. 34
4.1. Decision tree 34
4.1.1. SMOTE 34
4.1.2. ADASYN 35
4.2. Extra Tree 36
4.2.1. SMOTE 36
4.2.2. ADASYN 37
4.3. Extra Trees 38
4.3.1. SMOTE 38
4.3.2. ADASYN 39
4.4. Gradient Boosting 40
4.4.1. SMOTE 40
4.4.2. ADASYN 41
4.5. KNN 42
4.5.1. SMOTE 42
4.5.2. ADASYN 43
4.6. LightGBM 44
4.6.1. SMOTE 44
4.6.2. ADASYN 45
4.7. Logistic Regression 46
4.7.1. SMOTE 46
4.7.2. ADASYN 47
4.8. NuSVM 48

4.8.1. SMOTE 48
4.8.2. ADASYN.. 49
4.9. Random Forest.. 50
4.9.1. SMOTE 50
4.9.2. ADASYN.. 51
4.10. XgBoost 52
4.10.1. SMOTE.. 52
4.10.2. ADASYN.. 53
5. Ranking, Absolute numbers, Incidence, Both sexes, in 2022 for thyroid cancer. ... 54
6. Optimized parameters of individual classifiers.. 54
6.1. Decision tree... 54
6.2. Extra Tree 55
6.3. Extra Trees 55
6.4. Gradient Boosting.. 55
6.5. KNN.. 56
6.6. LightGBM 56
6.7. Logistic Regression.. 56
6.8. NuSVM 56
6.9. Random Forest.. 57
6.10. XgBoost 57

1. Classifiers with default parameter values

1.1. Decision Tree

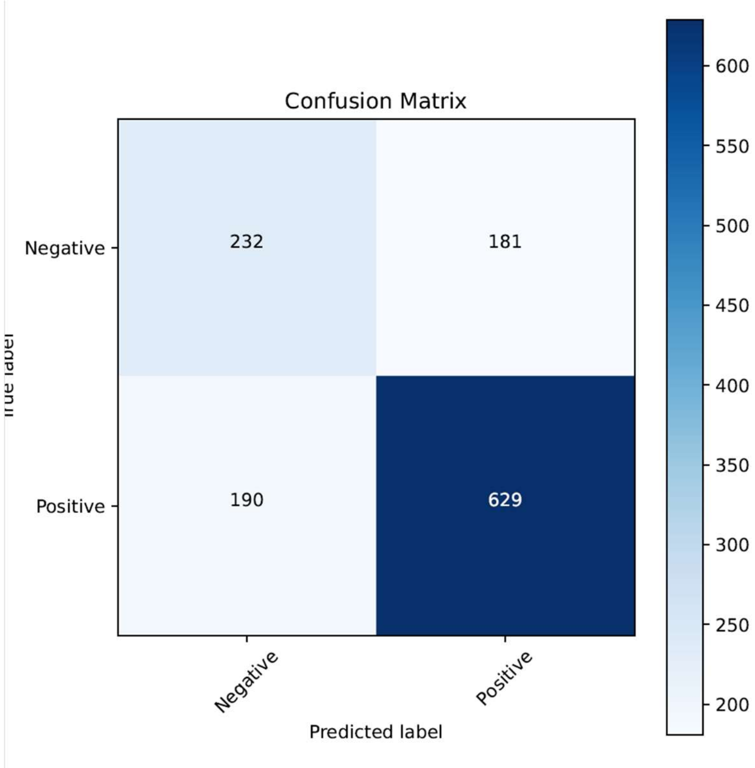

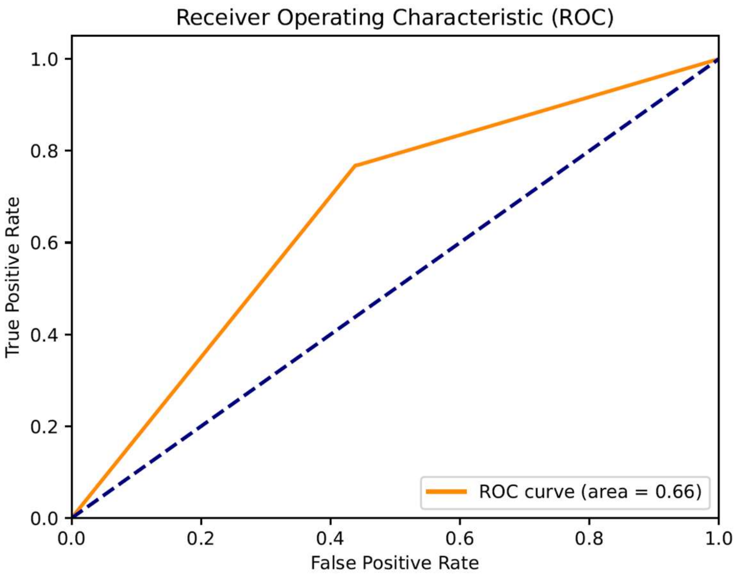

1.2. Extra Tree

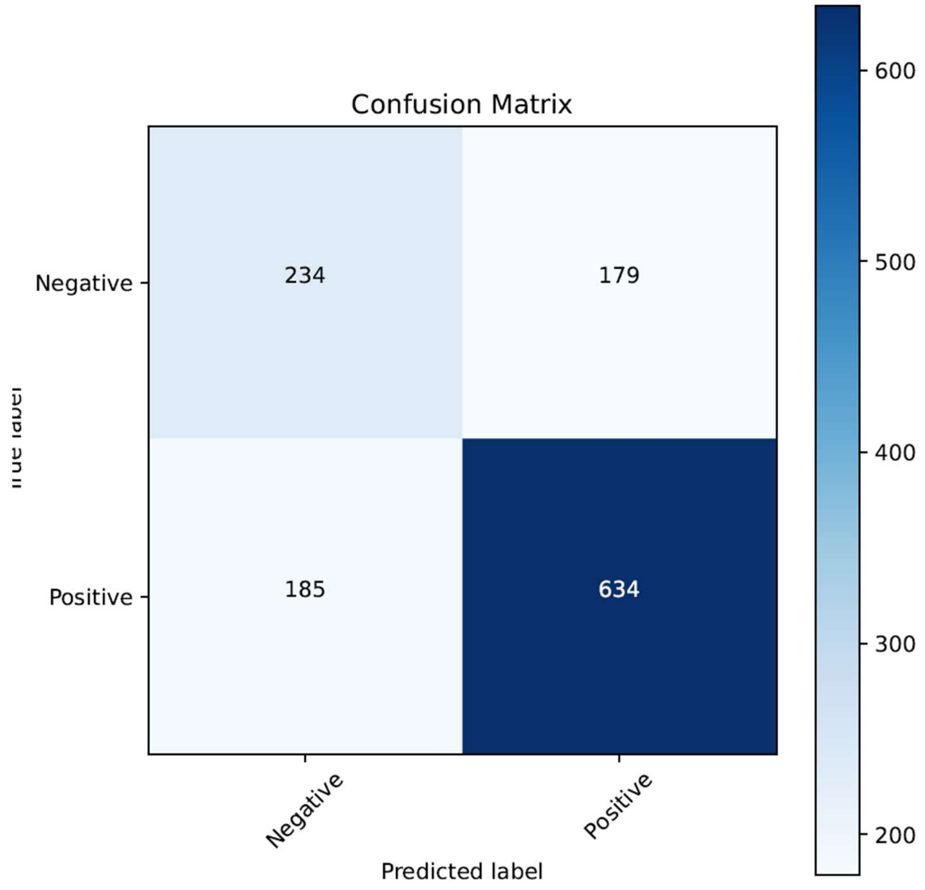

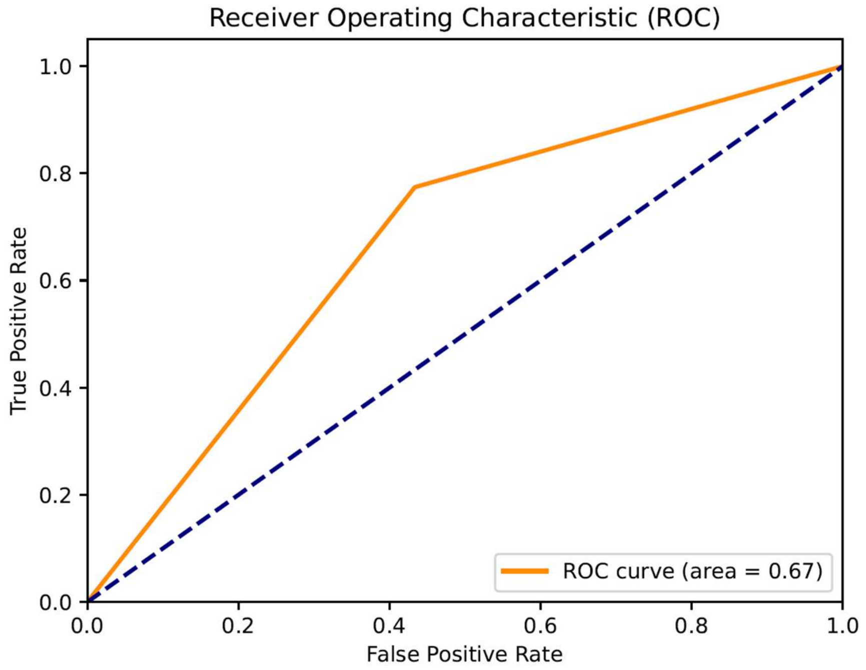

1.3. Extra Trees

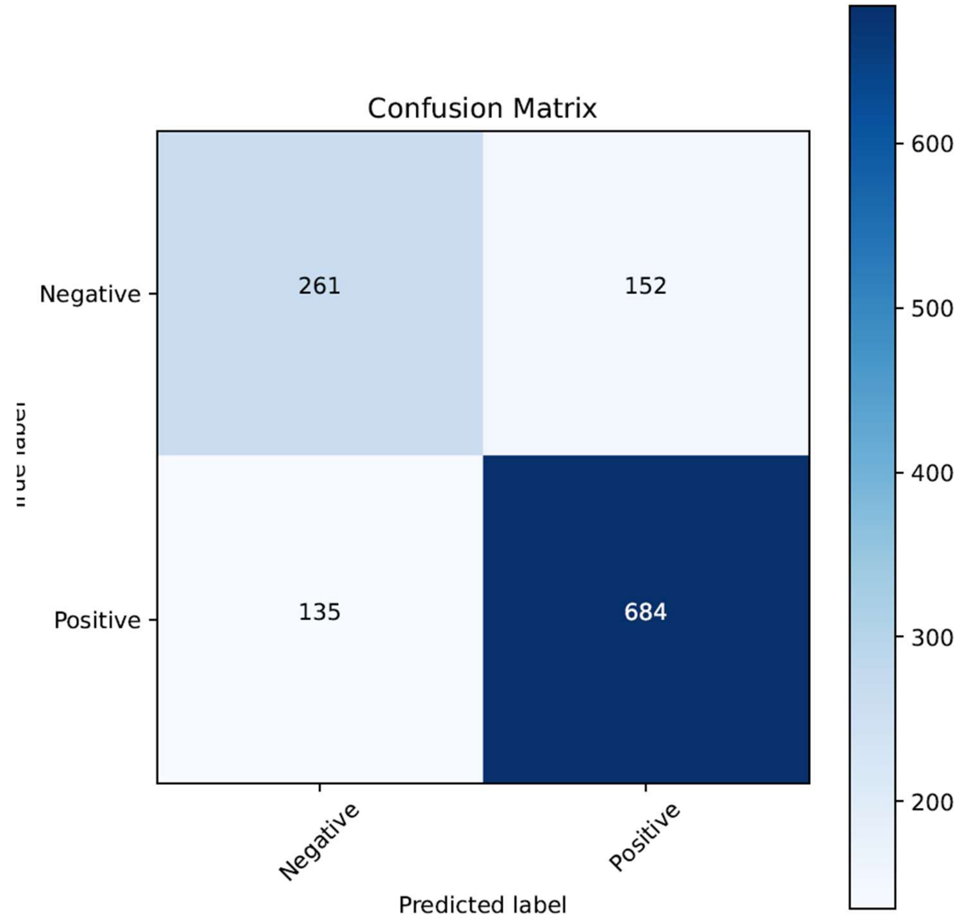

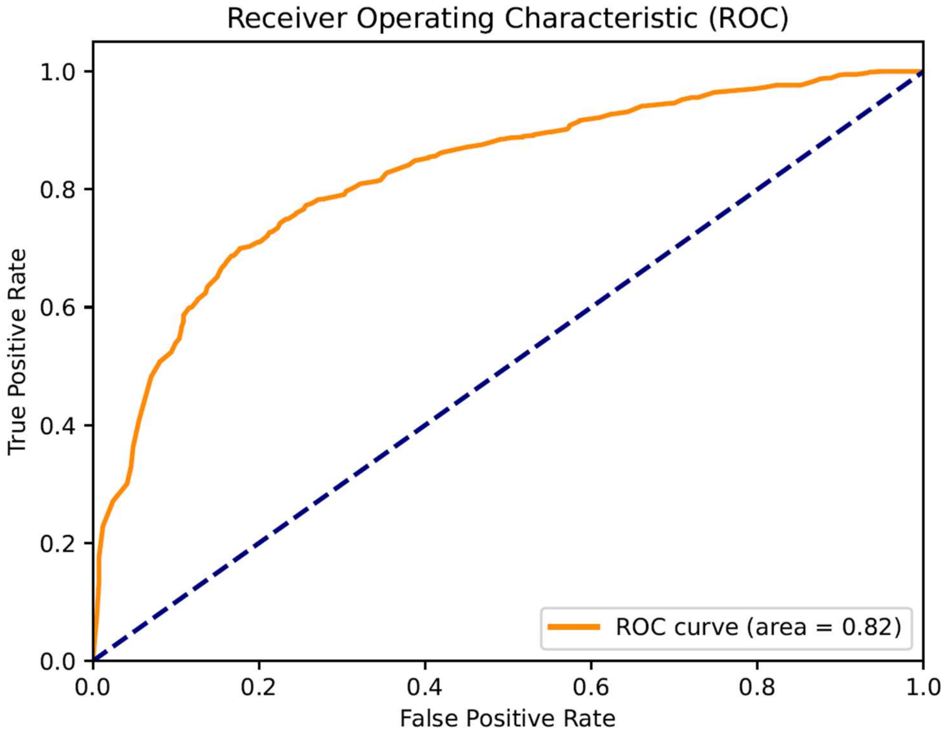

1.4. Gradient Boosting

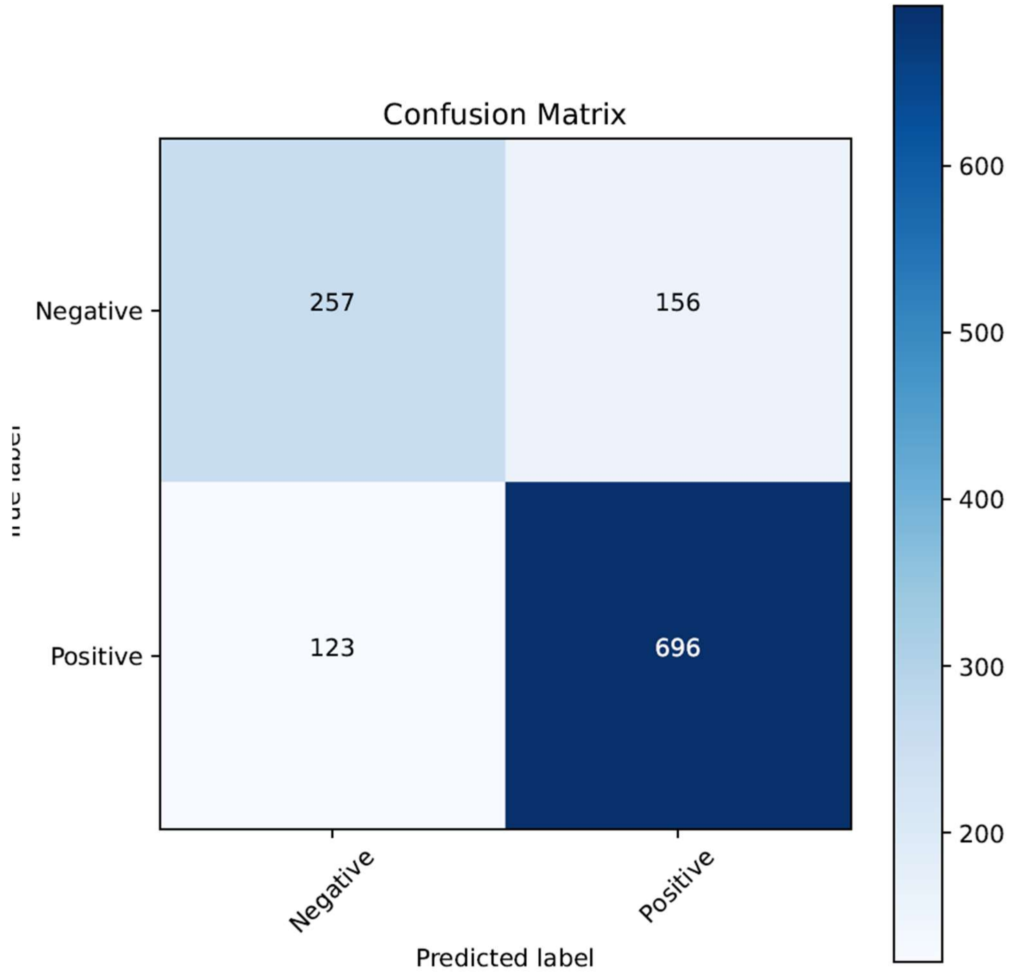

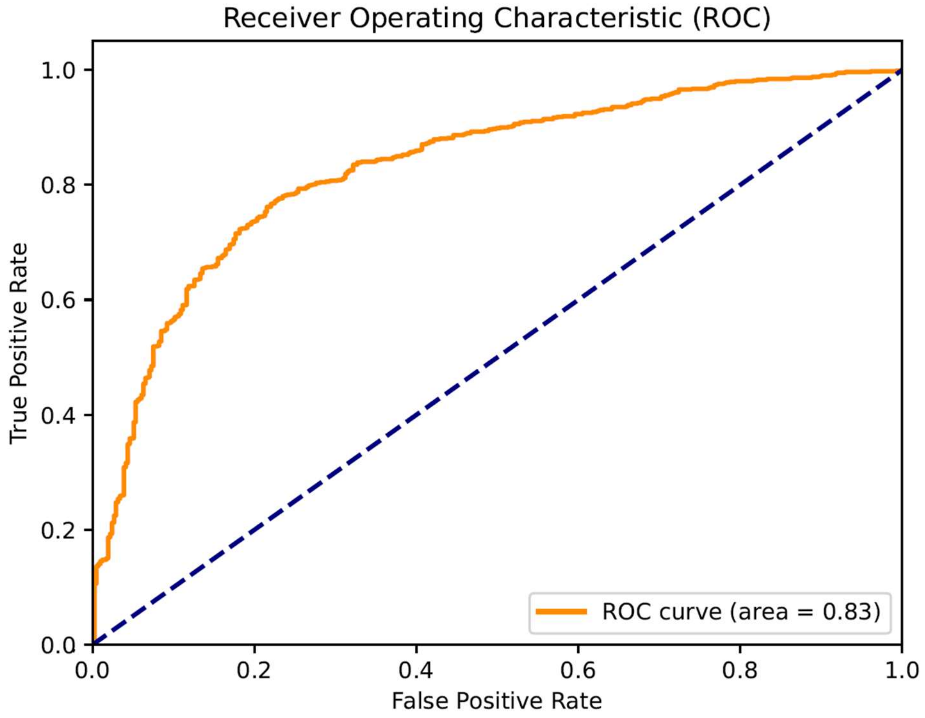

1.5. KNN

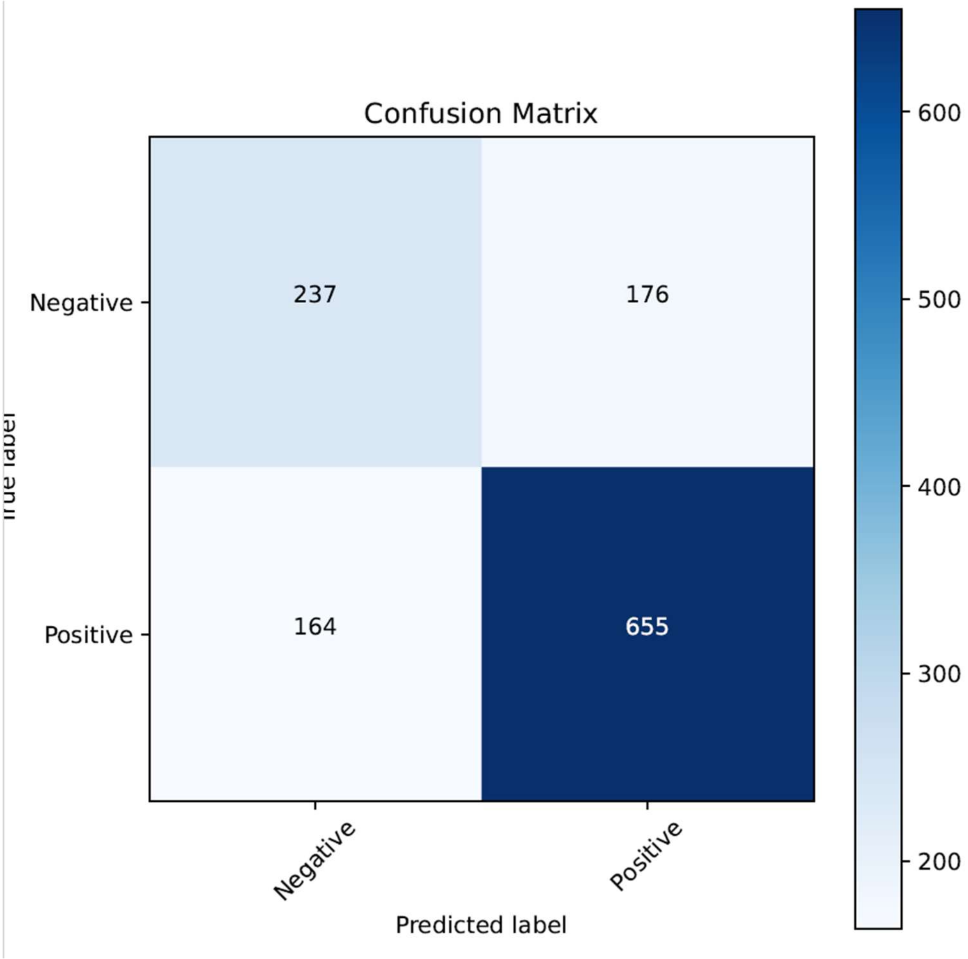

Confusion Matrix
Negative
Positive
237
176
164
655
True label
Negative
Positive
Predicted label
600
500
400
300
200

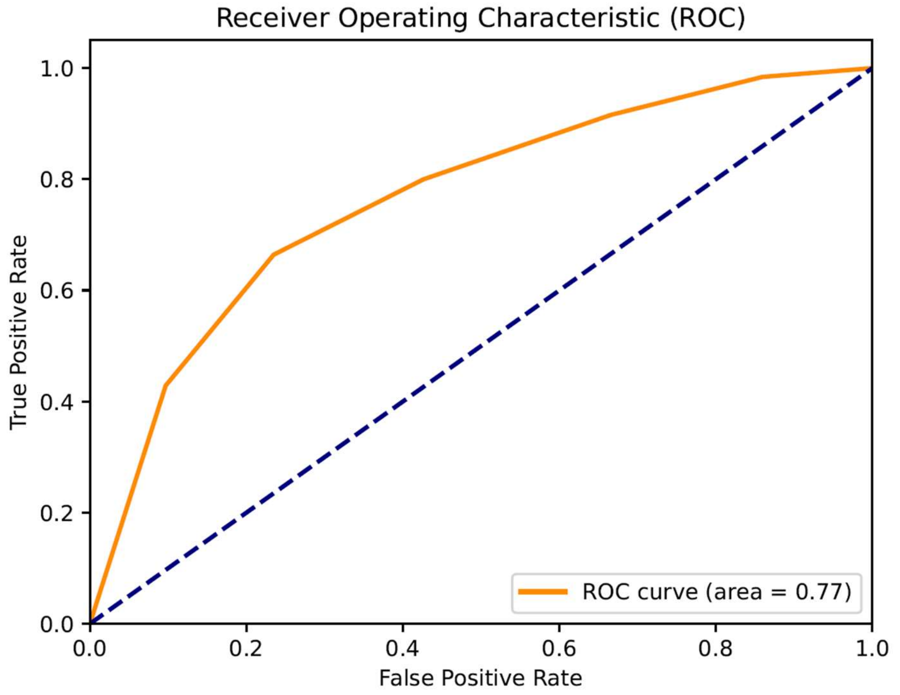

Receiver Operating Characteristic (ROC)
1.0
0.8
0.6
0.4
0.2
0.0
True Positive Rate
ROC curve (area = 0.77)
0.0
0.2
0.4
0.6
0.8
1.0
False Positive Rate

1.6. LightGBM

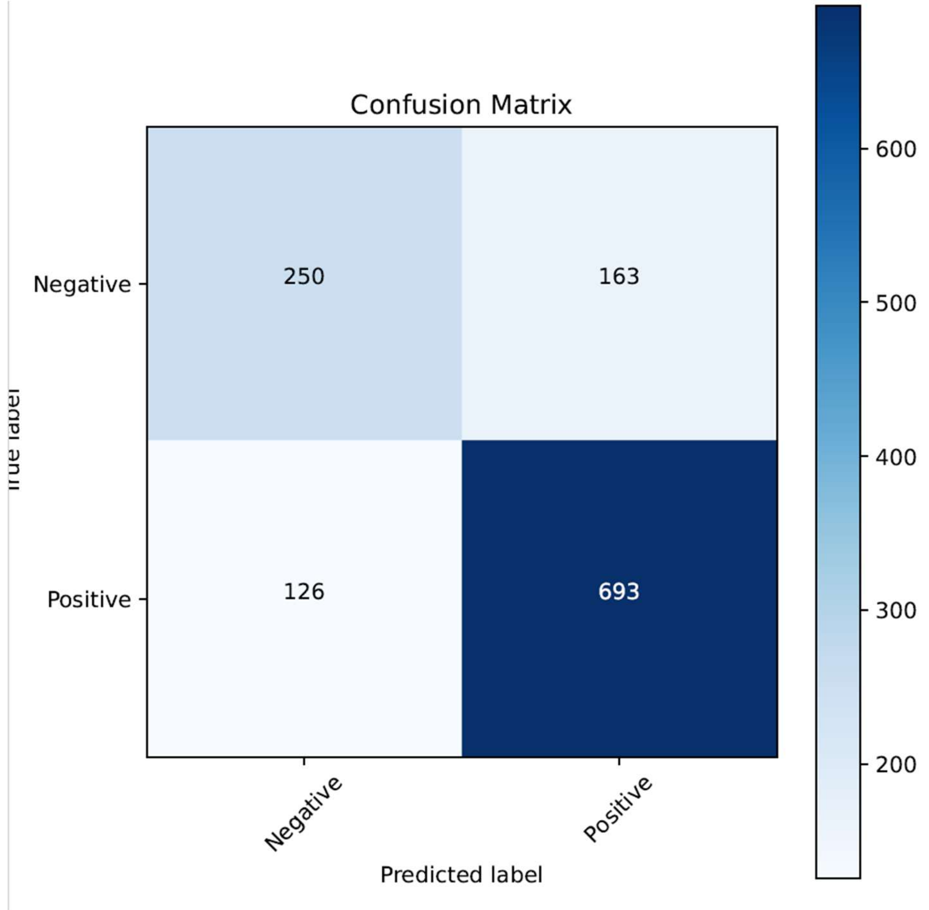

Confusion Matrix
Negative
Positive
250
163
126
693
Negative
Positive
Predicted label
600
500
400
300
200

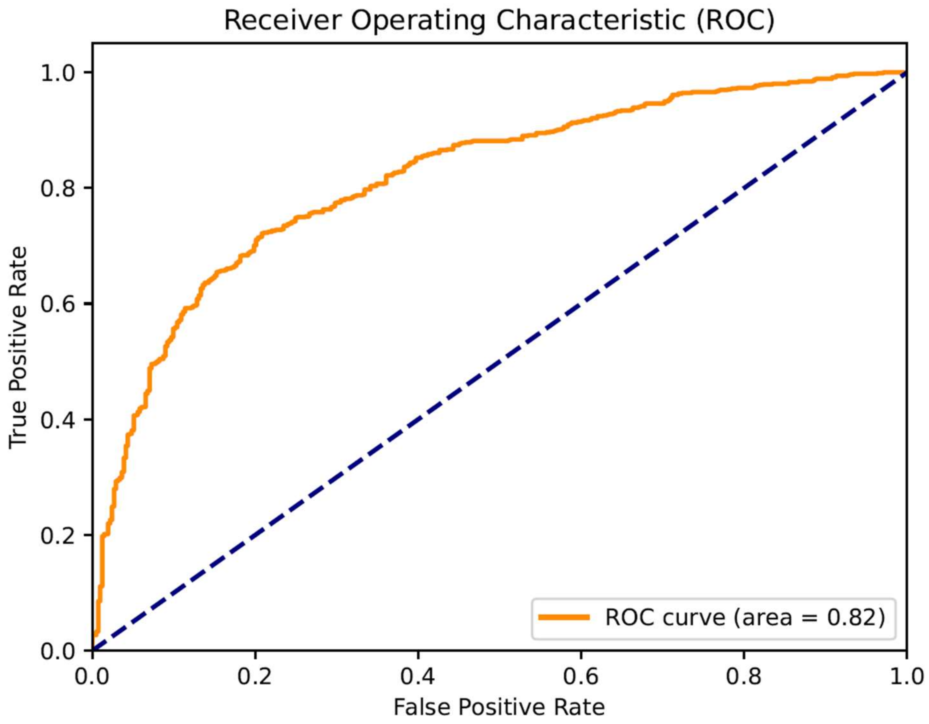

Receiver Operating Characteristic (ROC)
True Positive Rate
False Positive Rate
0.0
0.2
0.4
0.6
0.8
1.0
ROC curve (area = 0.82)

1.7. Logistic Regression

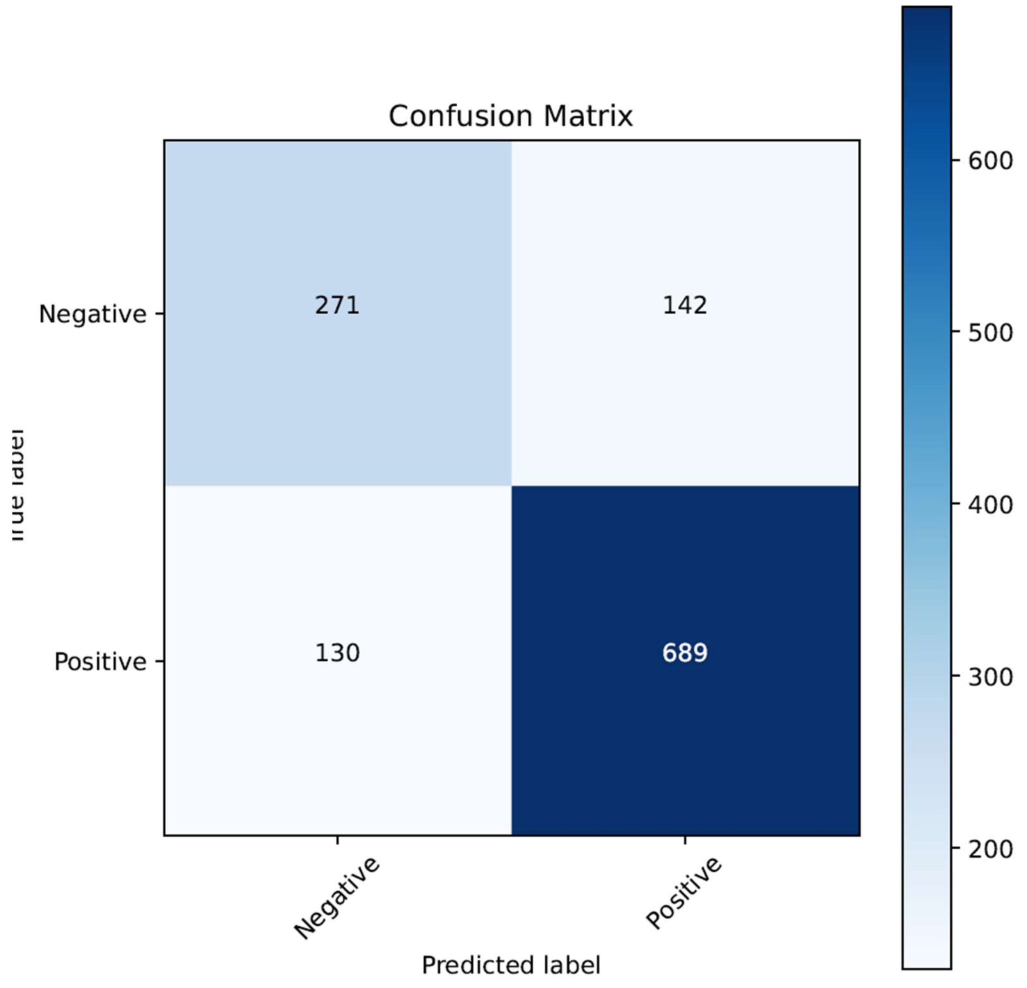

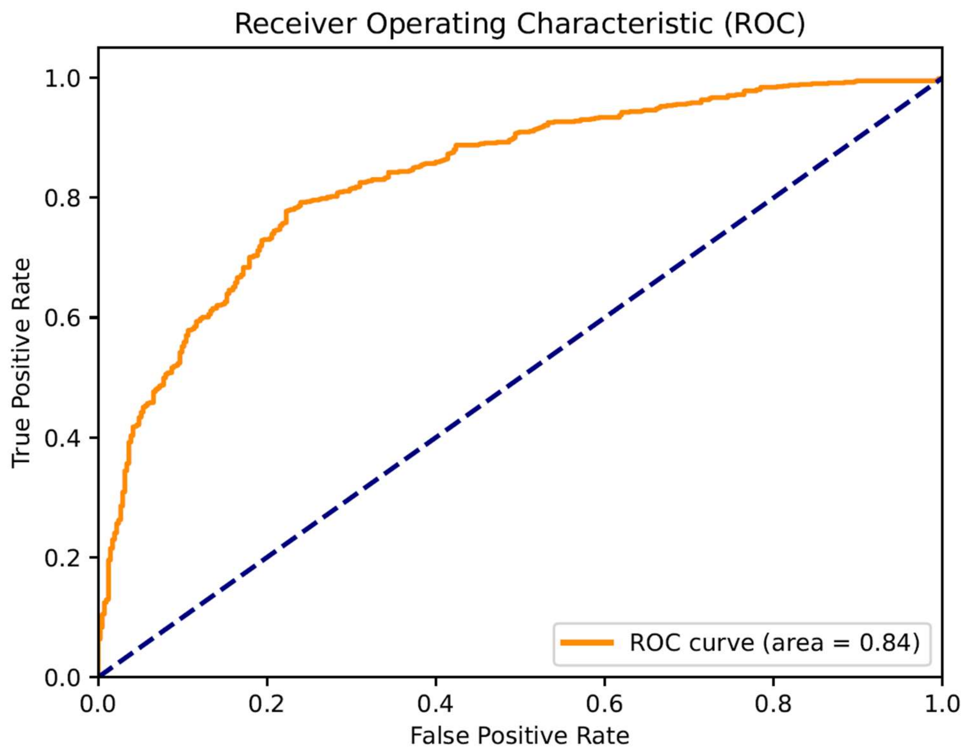

1.8. NuSVM

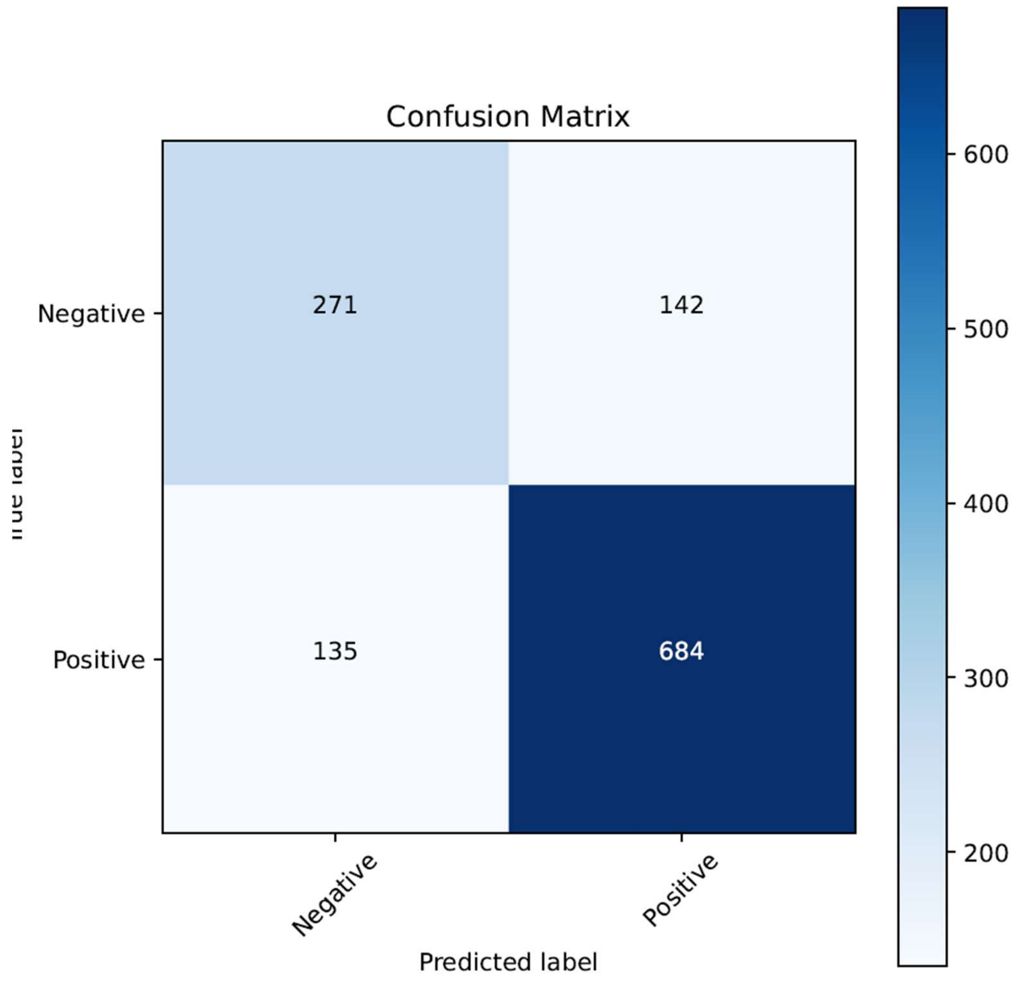

Confusion Matrix
Negative
Positive
271
142
135
684
Negative
Positive
Predicted label
600
500
400
300
200

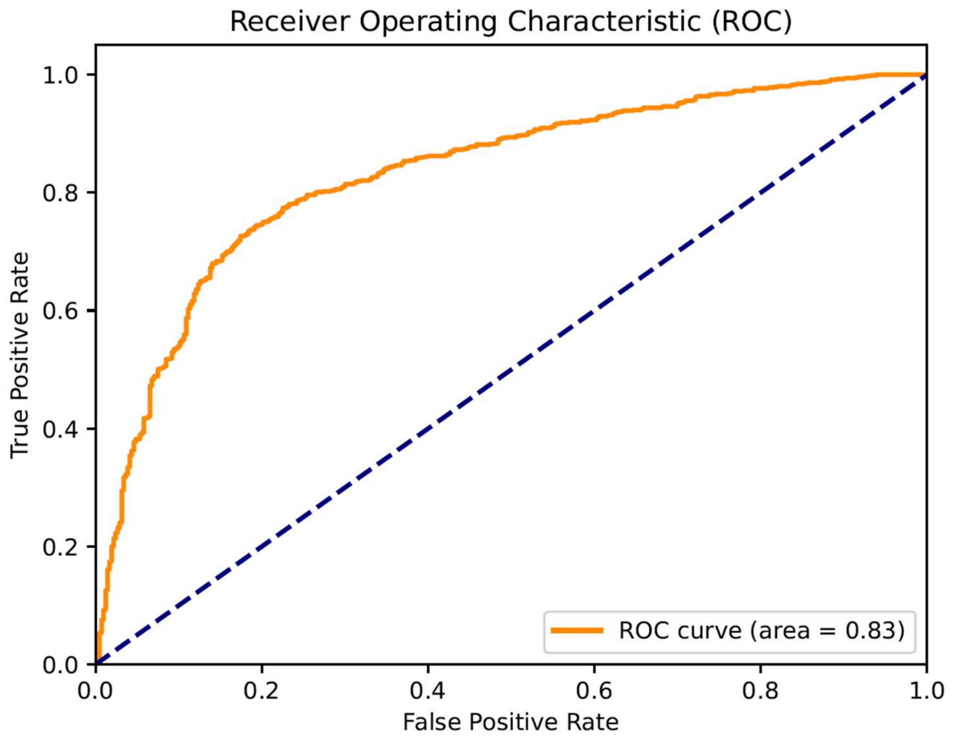

Receiver Operating Characteristic (ROC)
1.0
0.8
0.6
0.4
0.2
0.0
True Positive Rate
0.0
0.2
0.4
0.6
0.8
1.0
False Positive Rate
ROC curve (area = 0.83)

1.9. Random Forest

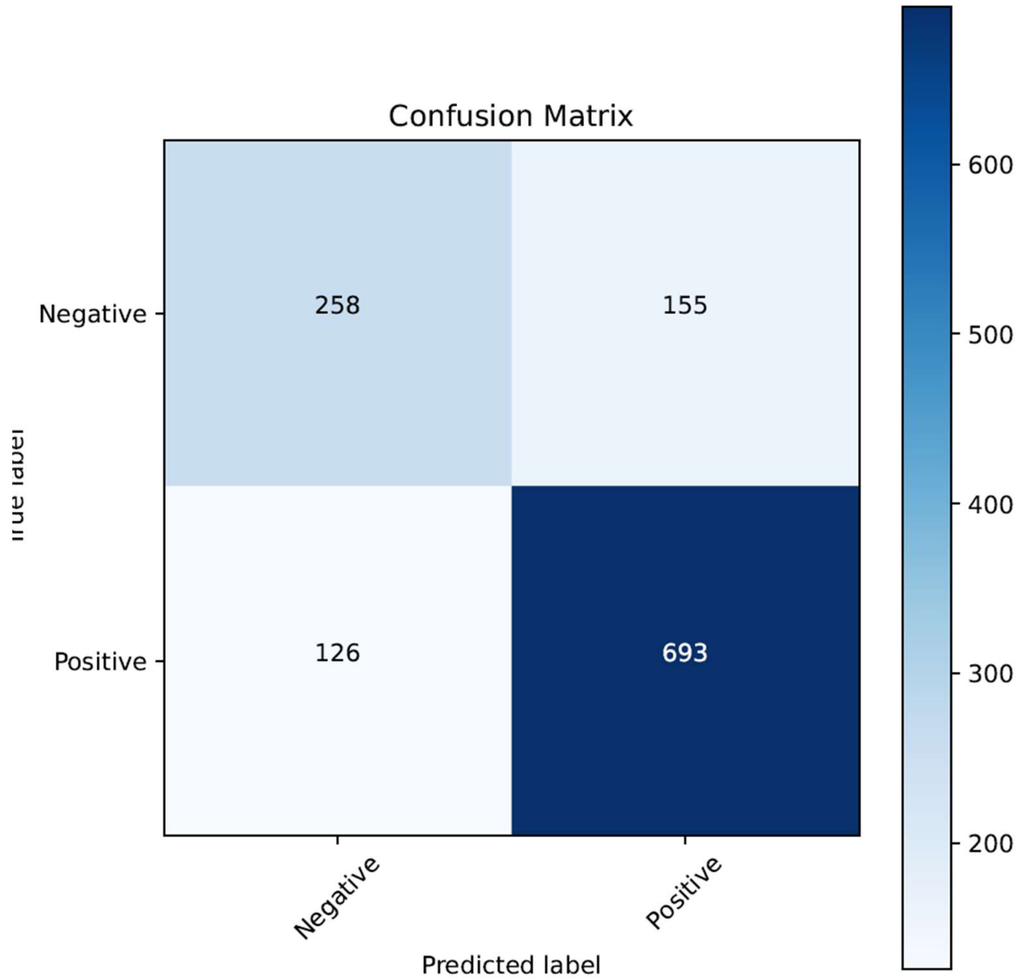

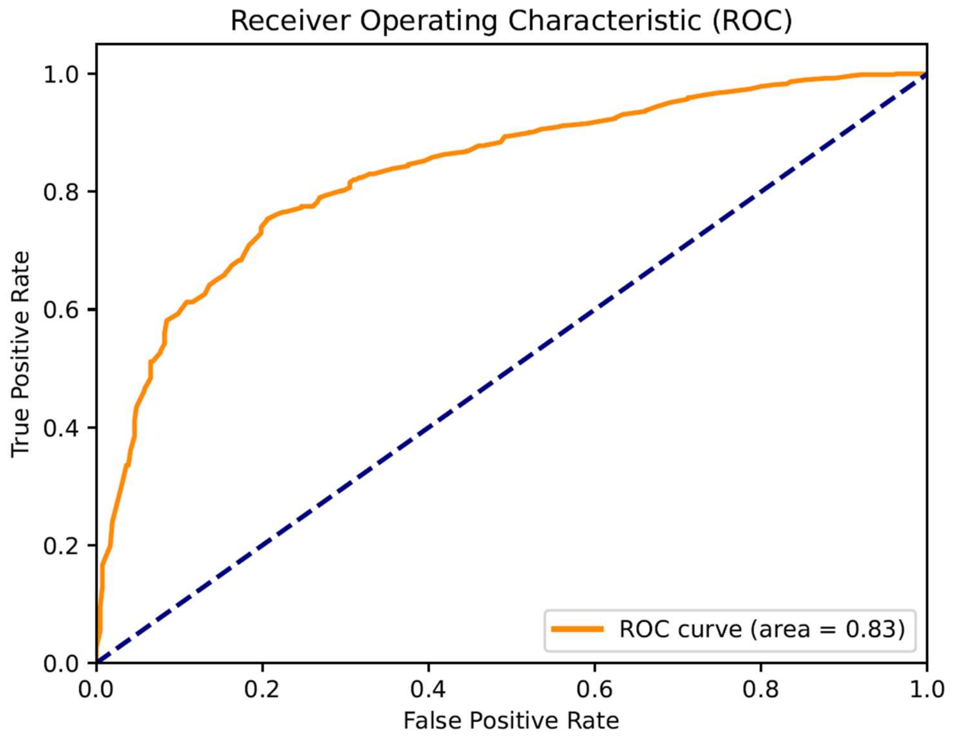

1.10. XgBoost

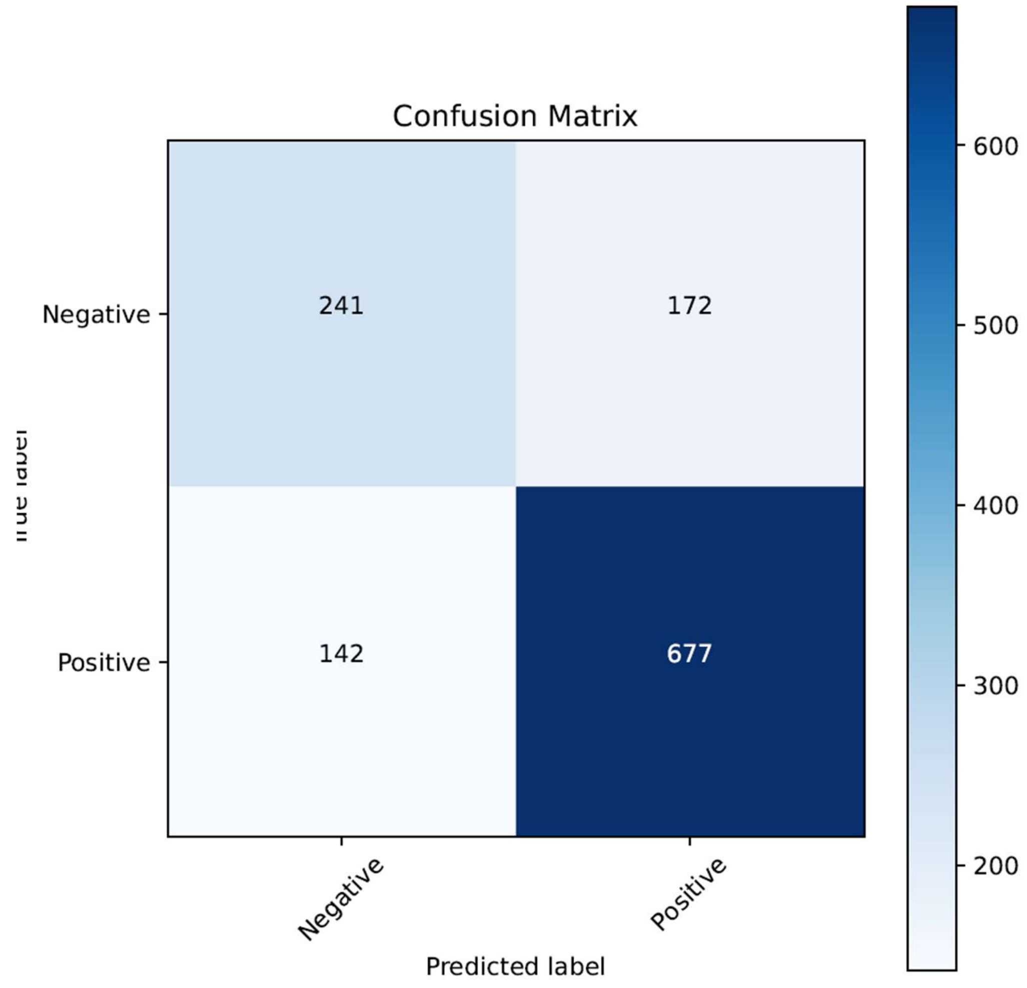
Confusion Matrix
Negative
Positive
241
172
142
677
Negative
Positive
Predicted label
600
500
400
300
200

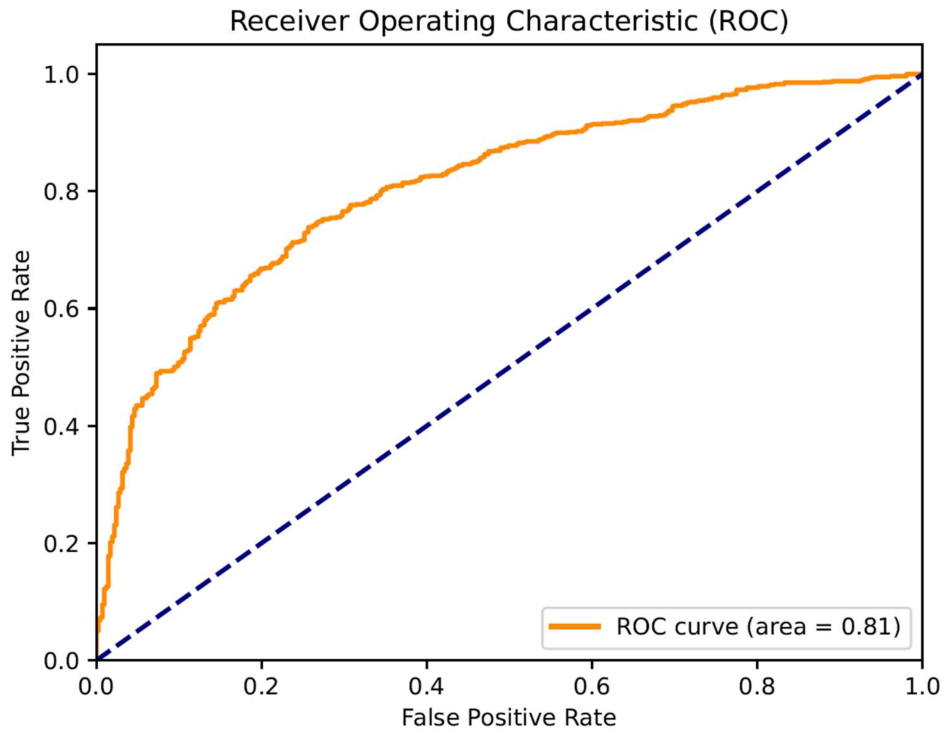
Receiver Operating Characteristic (ROC)
True Positive Rate
False Positive Rate
0.0
0.2
0.4
0.6
0.8
1.0
ROC curve (area = 0.81)

2. Optimization of classifier parameters using the Naked Mole-Rat Algorithm

2.1. Decision Tree

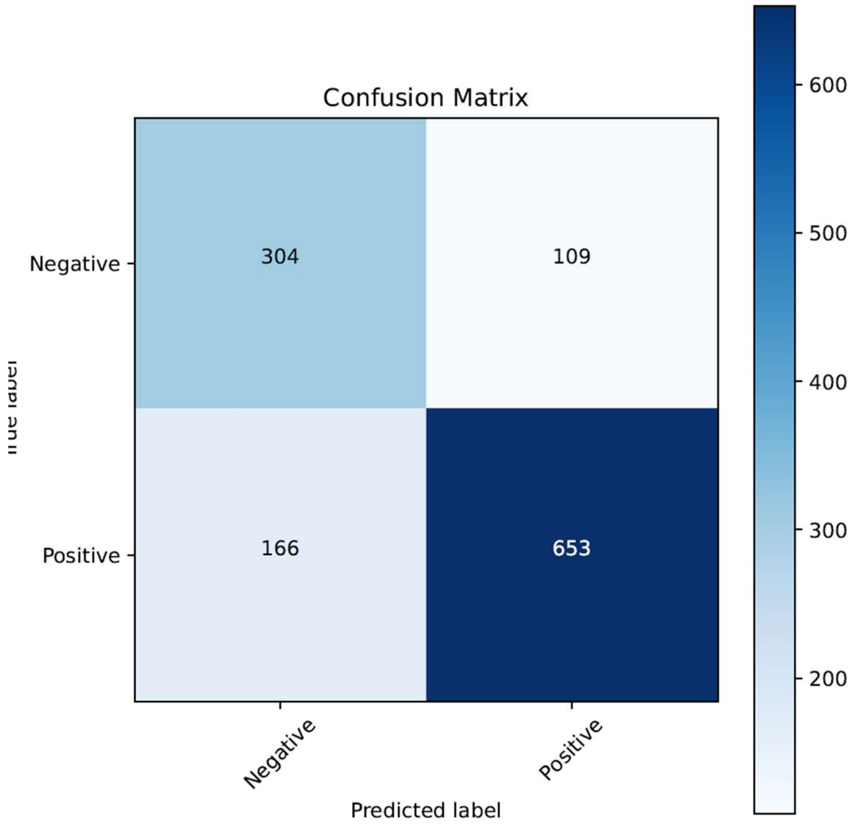

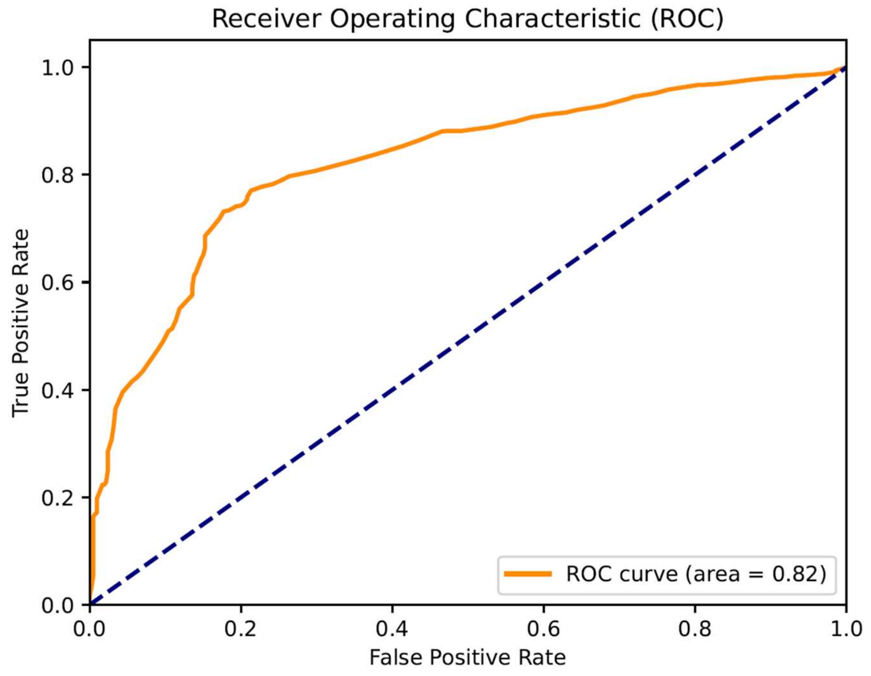

2.2. Extra Tree

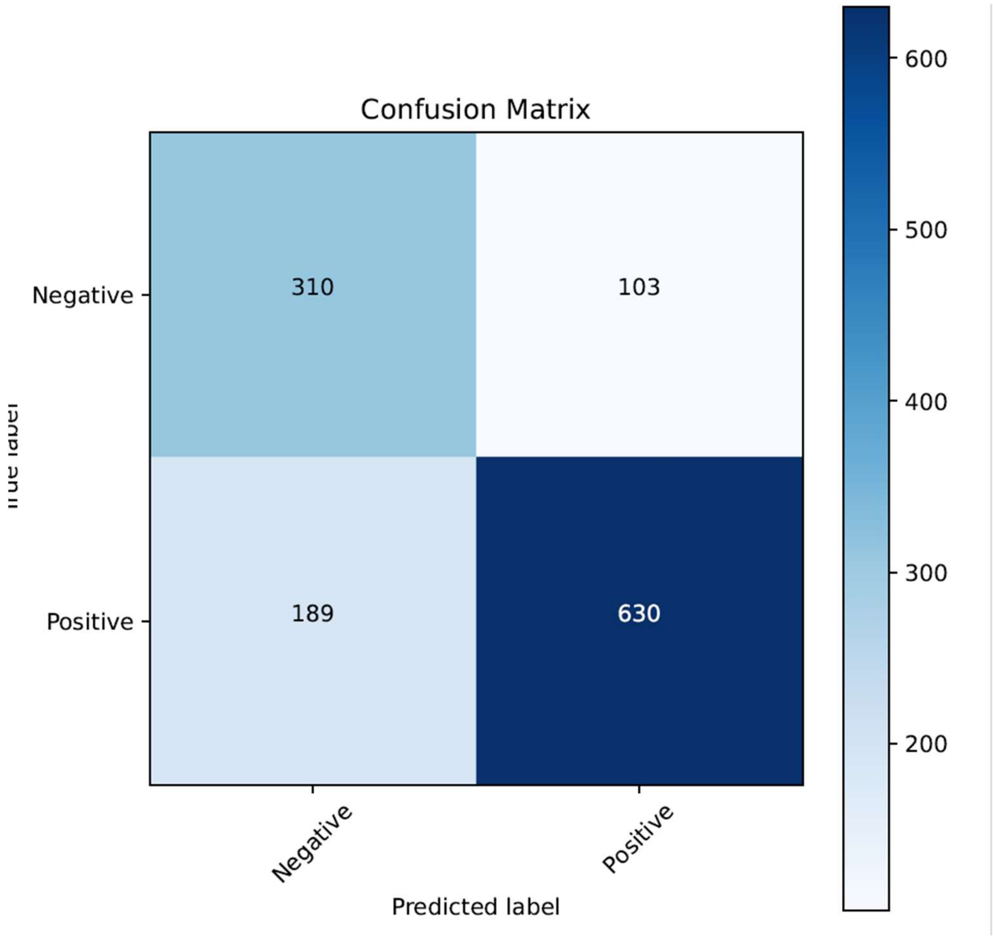

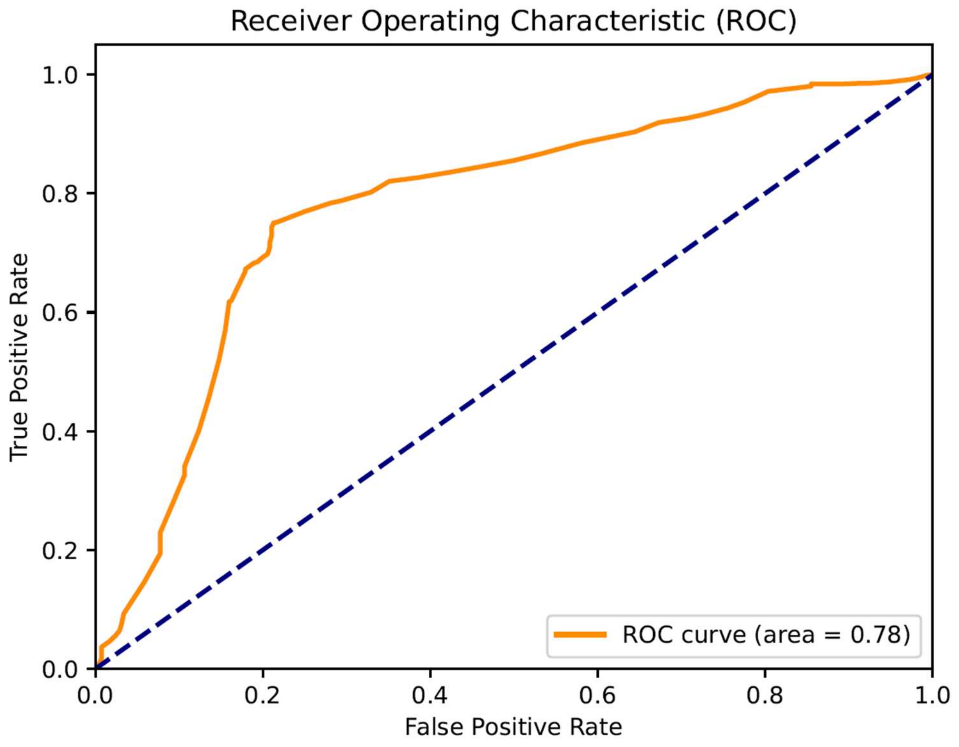

2.3. Extra Trees

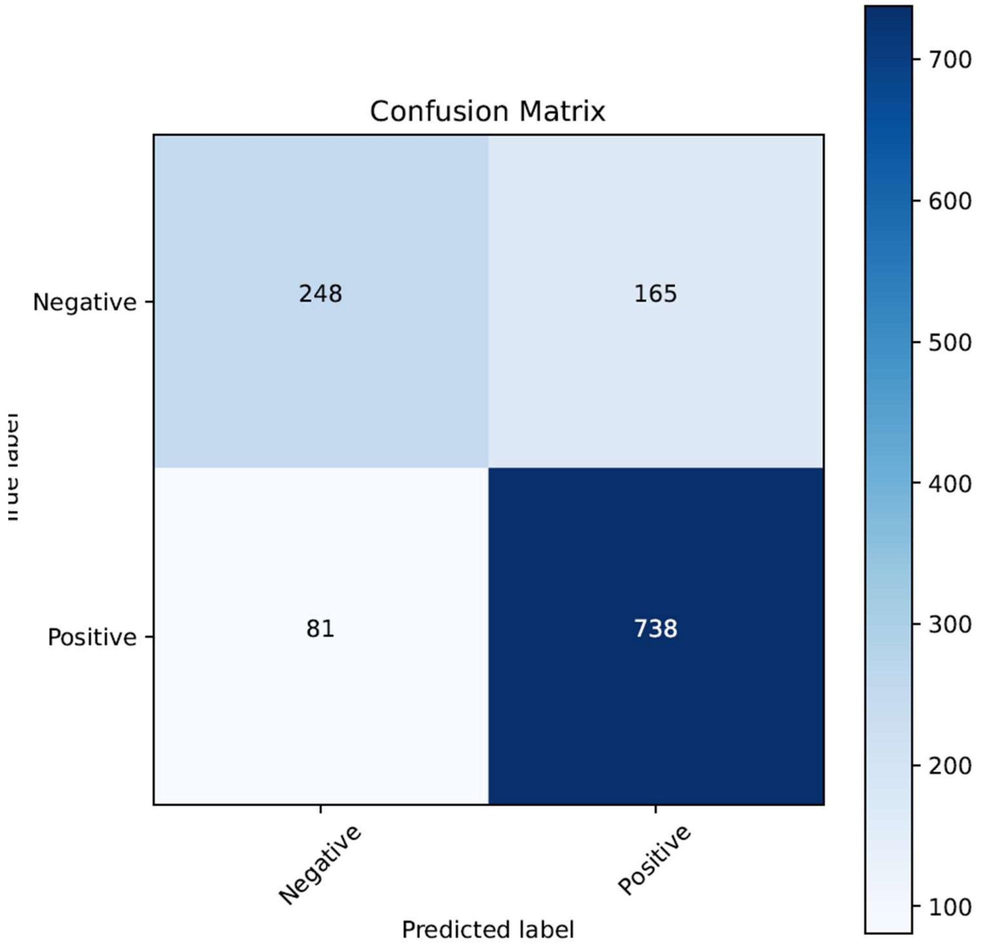

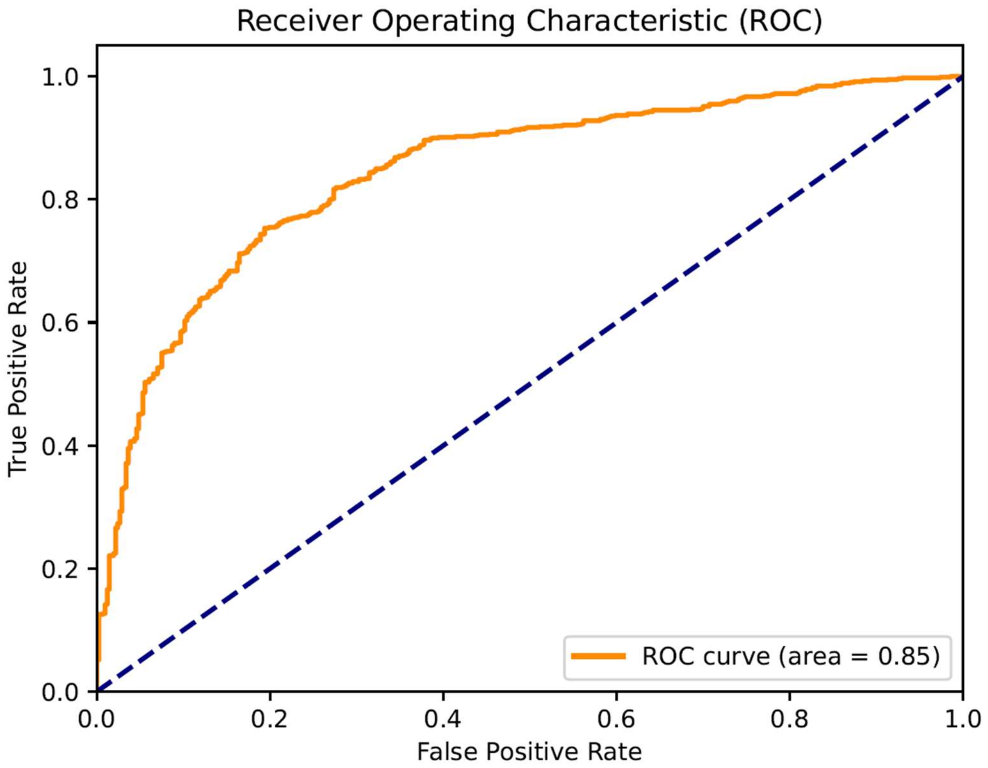

2.4. Gradient Boosting

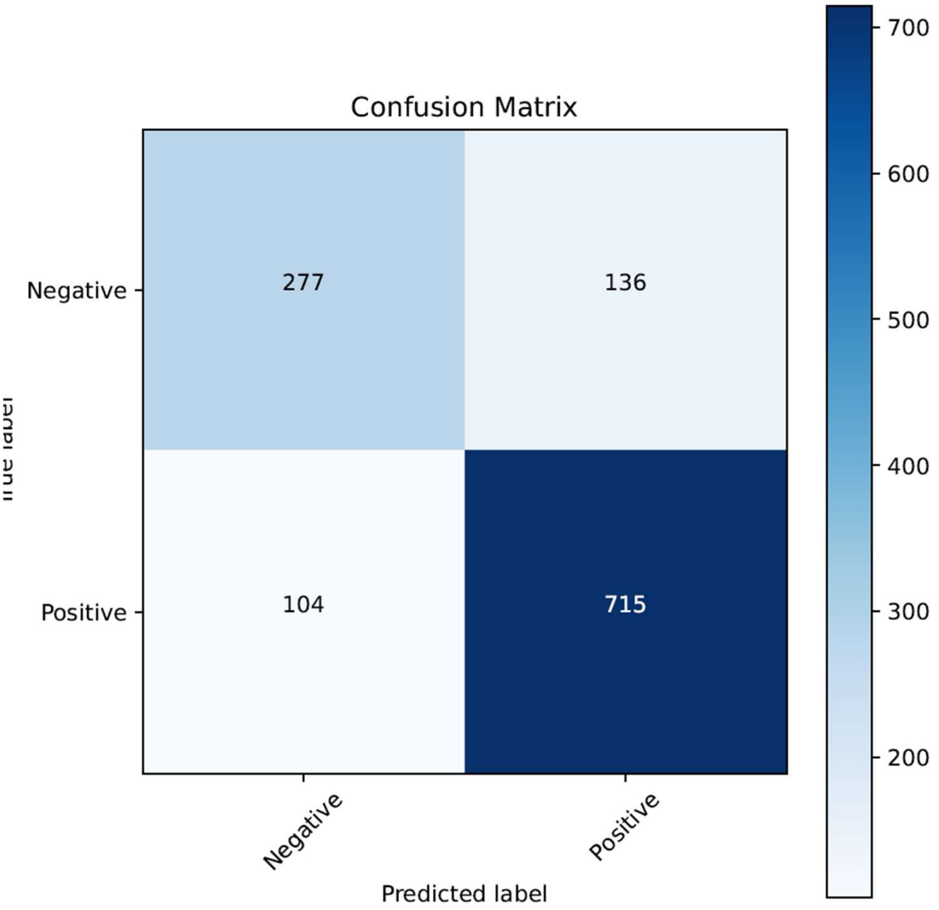

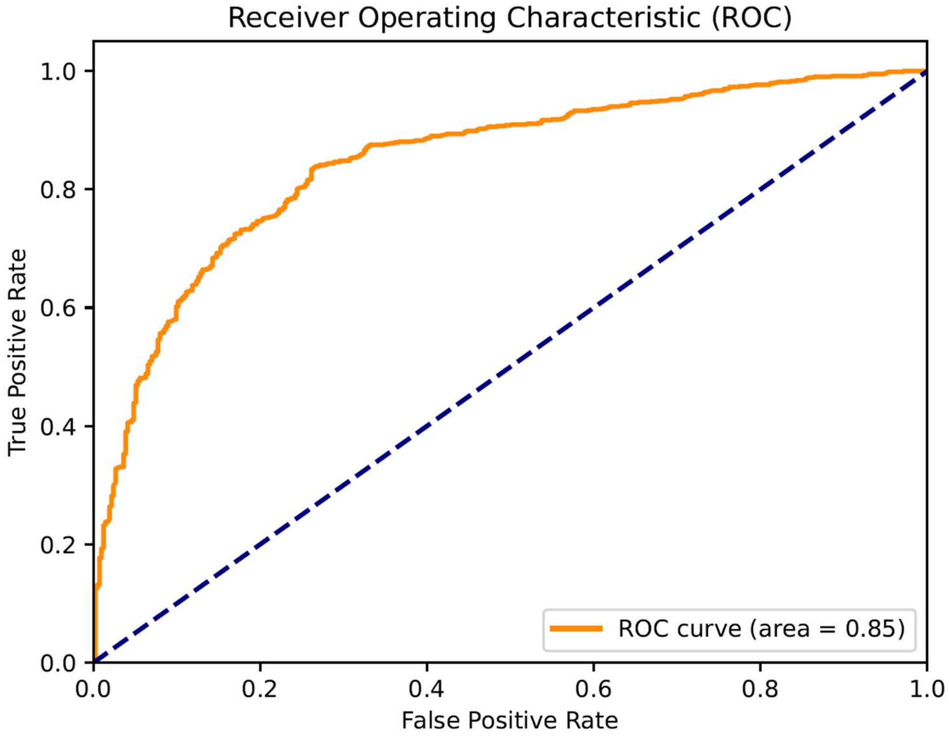

2.5. KNN

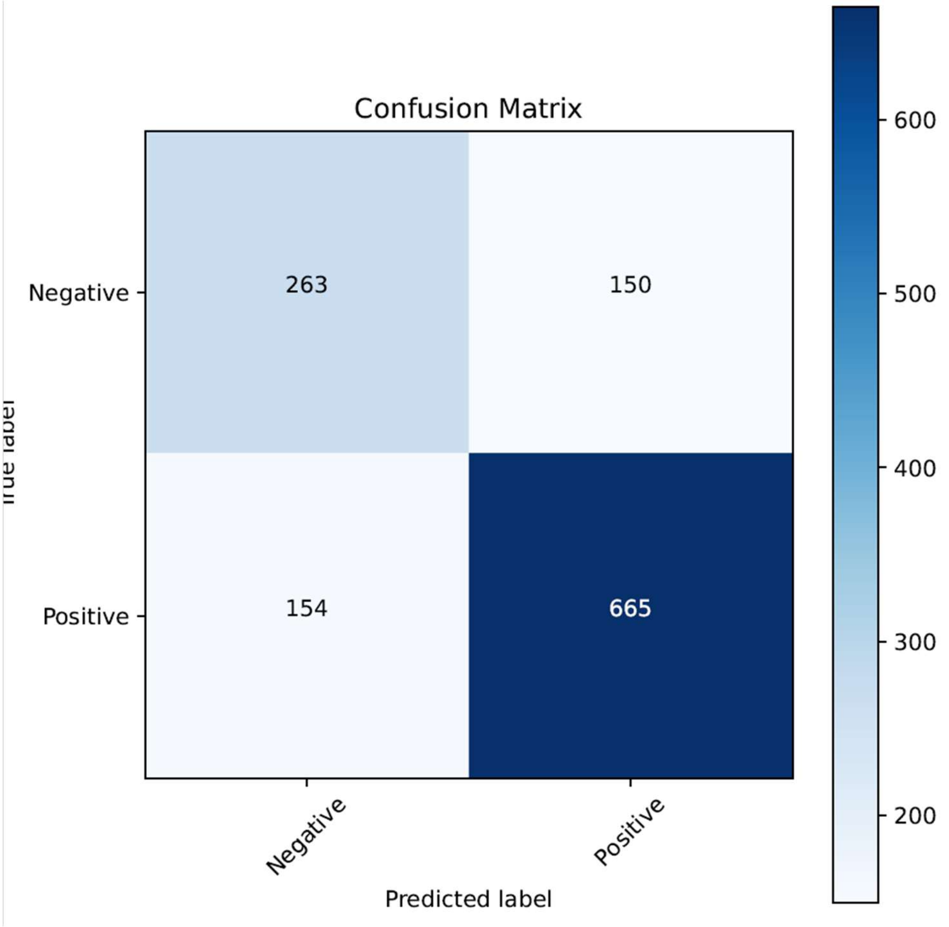

Confusion Matrix
Negative
Positive
263
150
154
665
True label
Predicted label
600
500
400
300
200

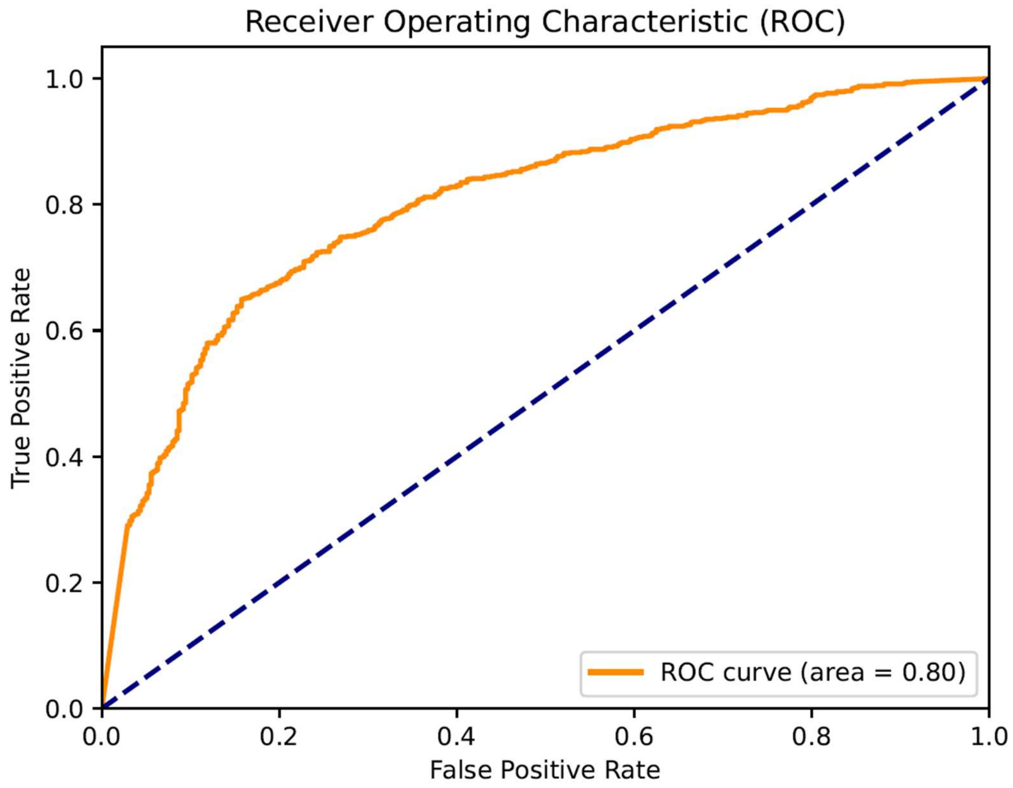

Receiver Operating Characteristic (ROC)
True Positive Rate
False Positive Rate
0.0
0.2
0.4
0.6
0.8
1.0
ROC curve (area = 0.80)

2.6. LightGBM

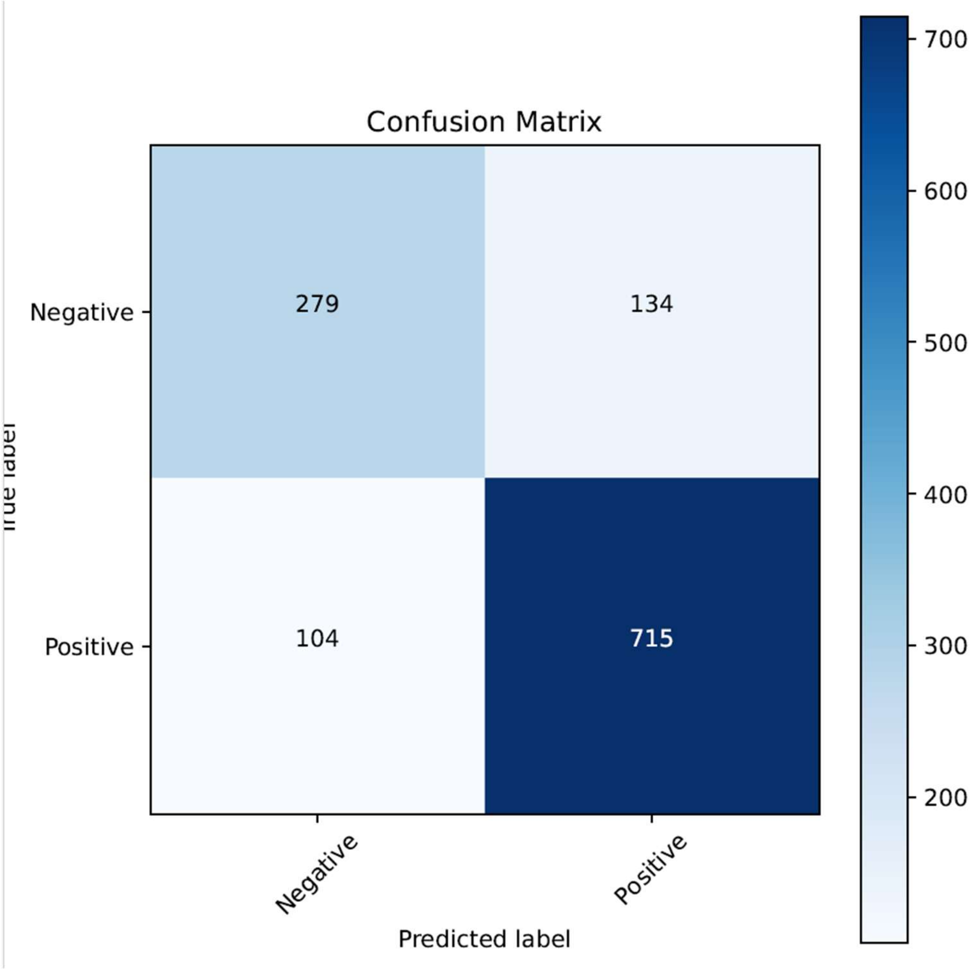

Confusion Matrix
True label
Negative
Positive
279
134
104
715
Negative
Positive
Predicted label
700
600
500
400
300
200

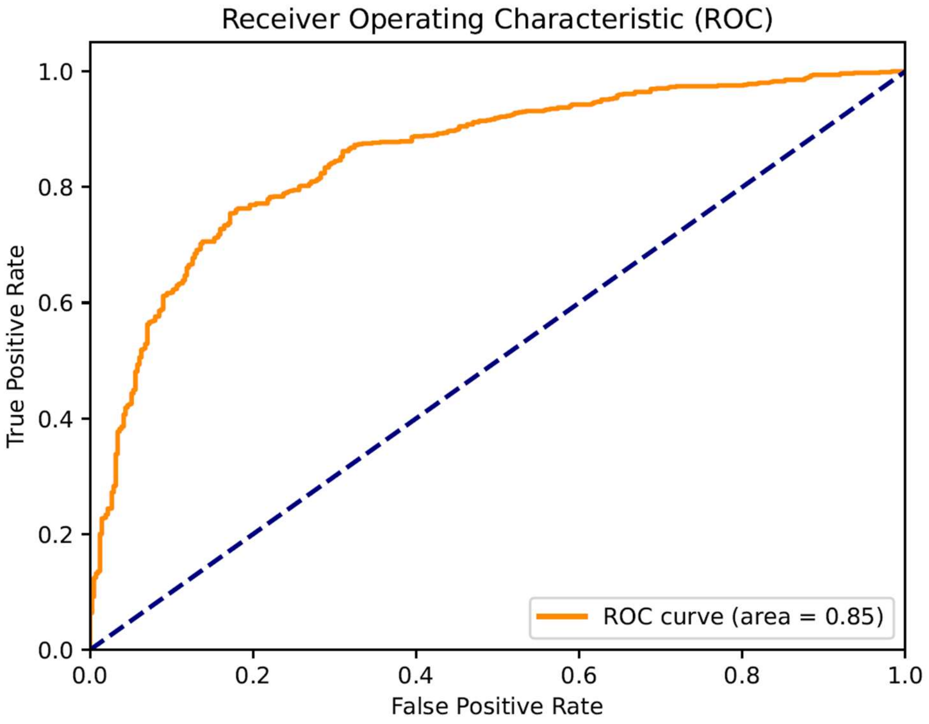

Receiver Operating Characteristic (ROC)
True Positive Rate
False Positive Rate
0.0
0.2
0.4
0.6
0.8
1.0
ROC curve (area = 0.85)

2.7. Logistic Regression

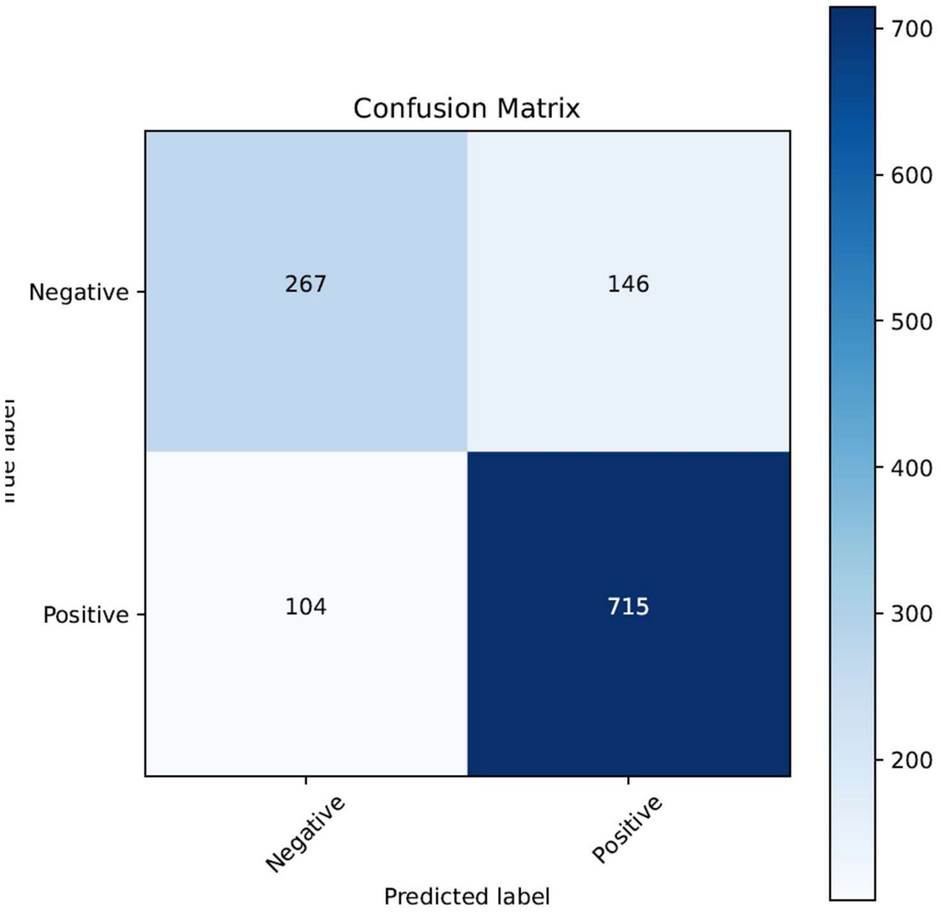

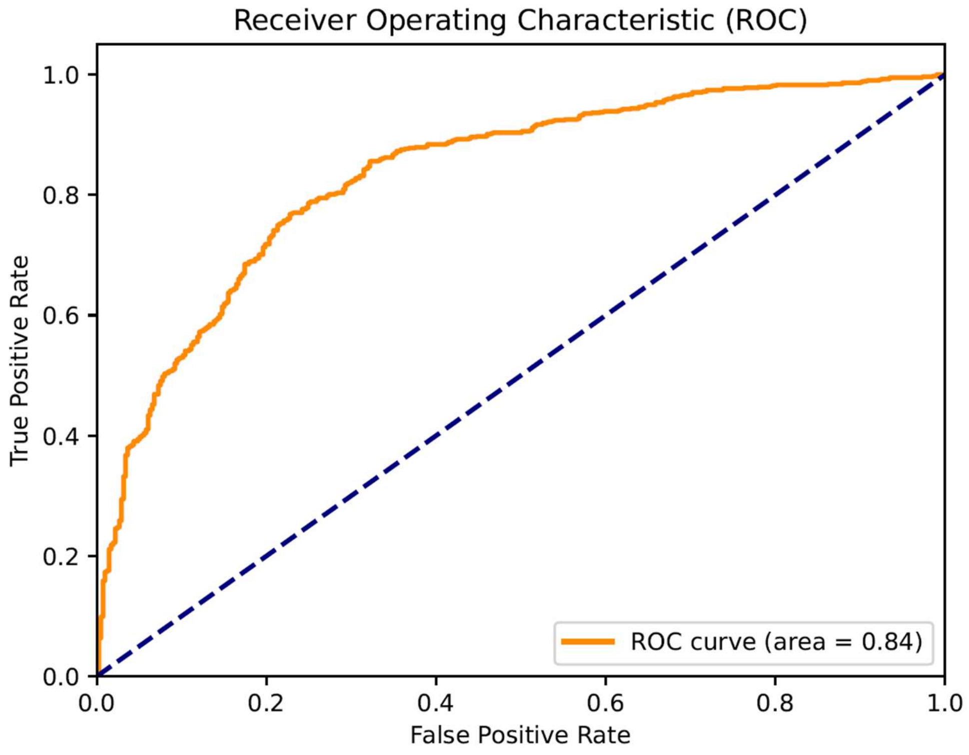

2.8. NuSVM

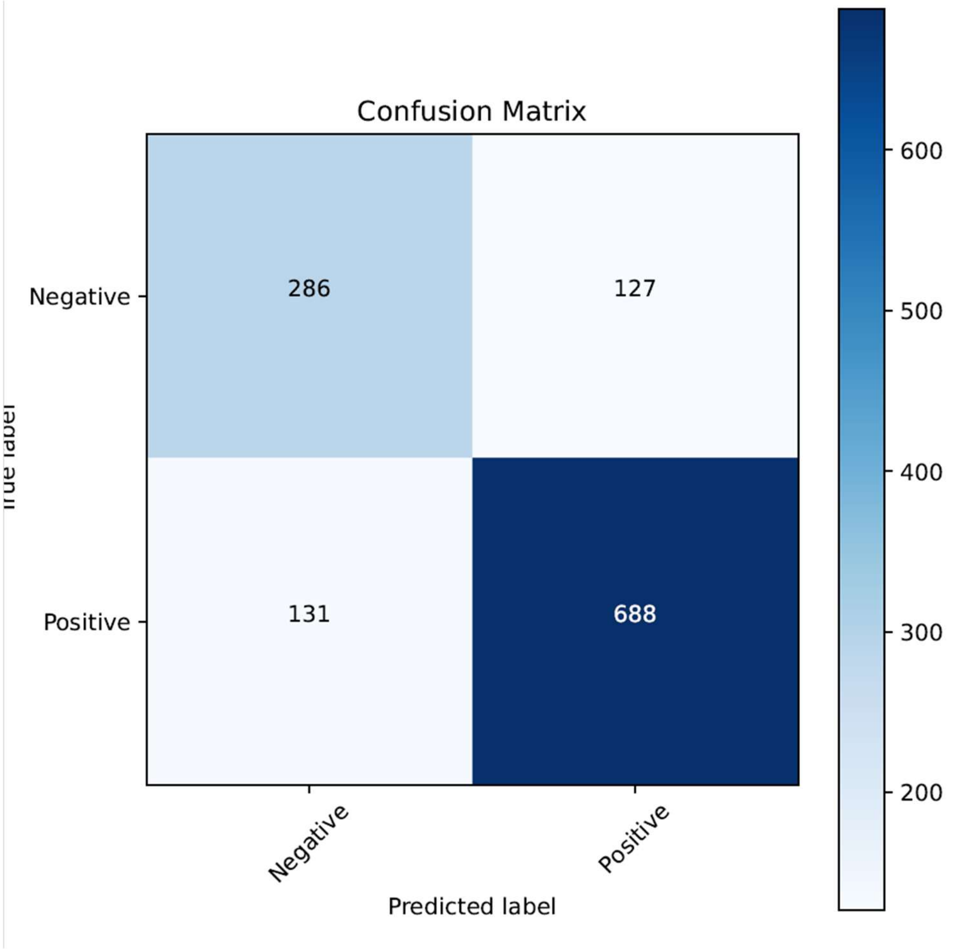

Confusion Matrix
Negative
Positive
286
127
131
688
True label
Negative
Positive
Predicted label
600
500
400
300
200

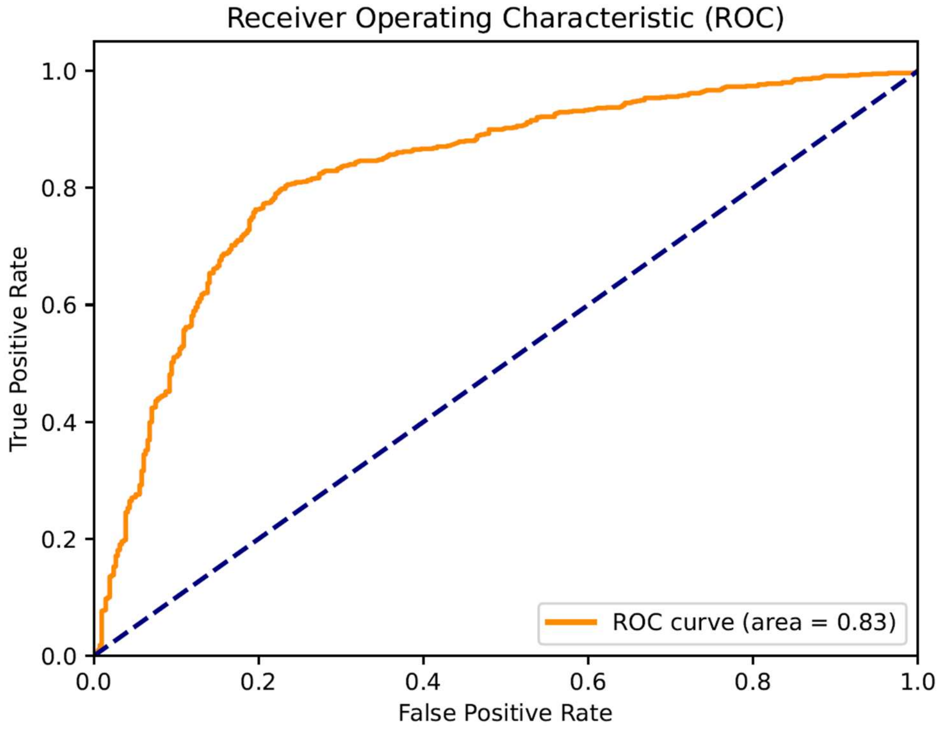

Receiver Operating Characteristic (ROC)
1.0
0.8
0.6
0.4
0.2
0.0
True Positive Rate
0.0
0.2
0.4
0.6
0.8
1.0
False Positive Rate
ROC curve (area = 0.83)

2.9. Random Forest

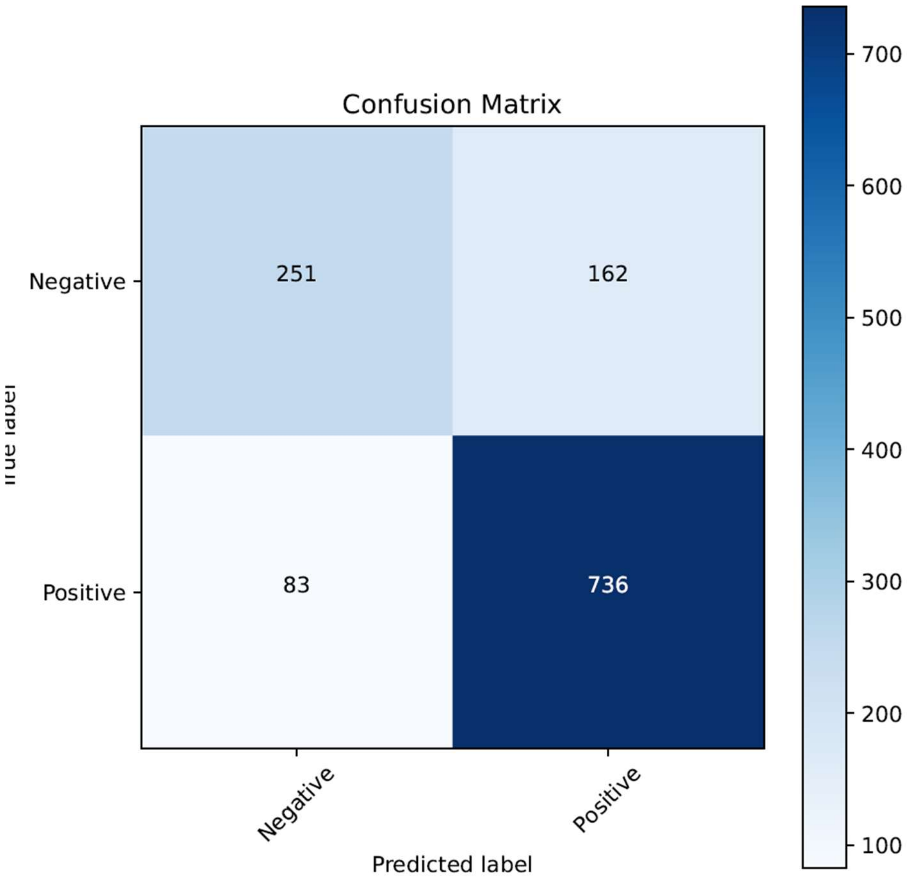

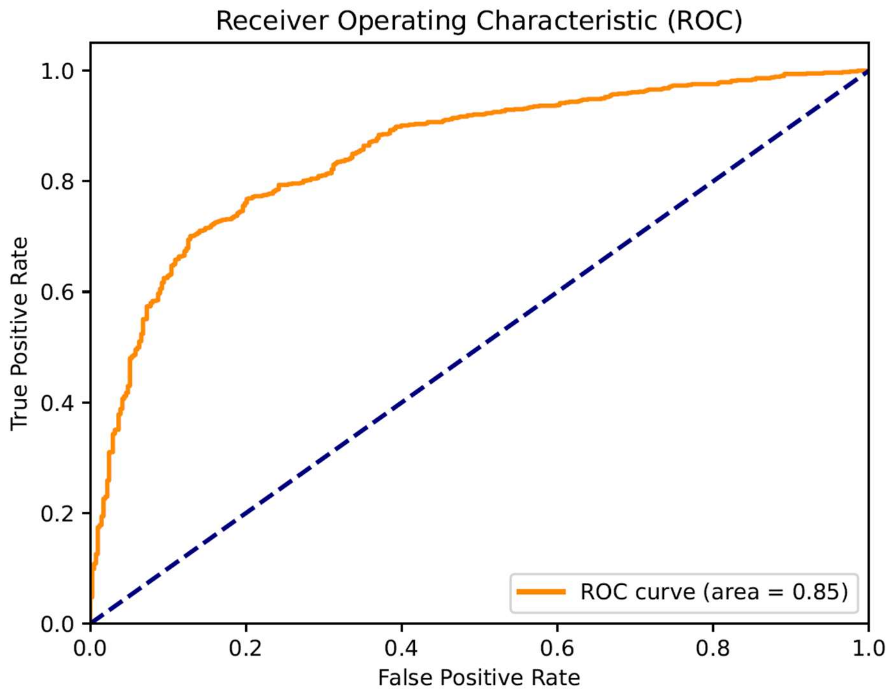

2.10. XgBoost

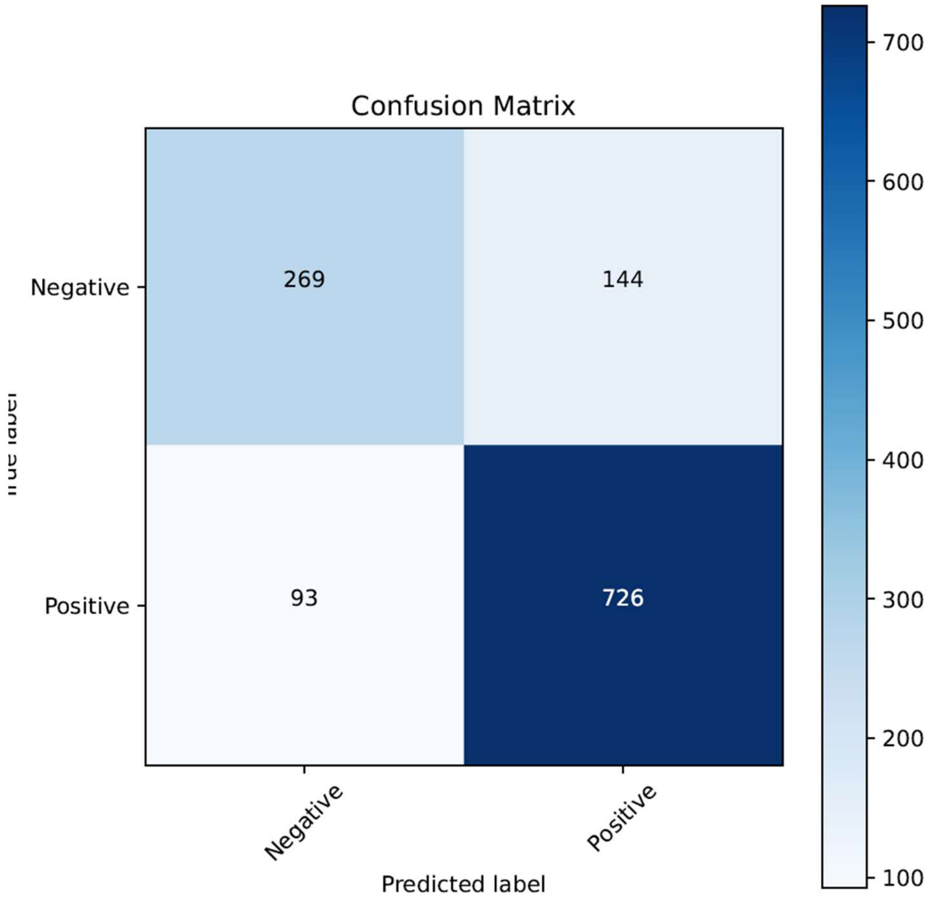
Confusion Matrix
Negative
Positive
269
144
93
726
Negative
Positive
Predicted label
700
600
500
400
300
200
100

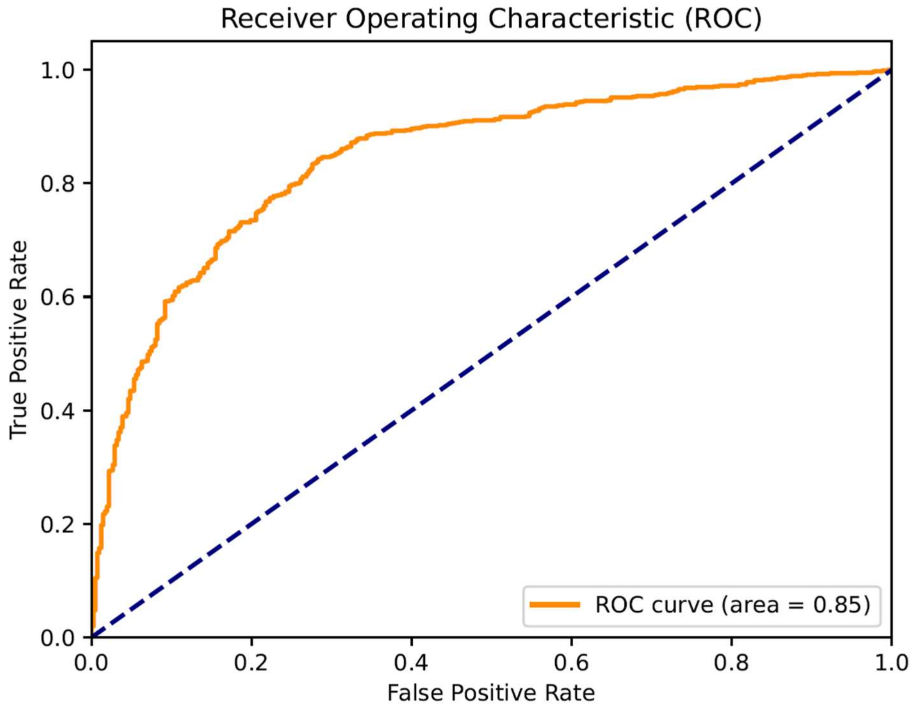
Receiver Operating Characteristic (ROC)
True Positive Rate
False Positive Rate
ROC curve (area = 0.85)
0.0
0.2
0.4
0.6
0.8
1.0

3. Optimization of classifier parameters and feature selection using the Naked Mole-Rat Algorithm

3.1. Decision Tree

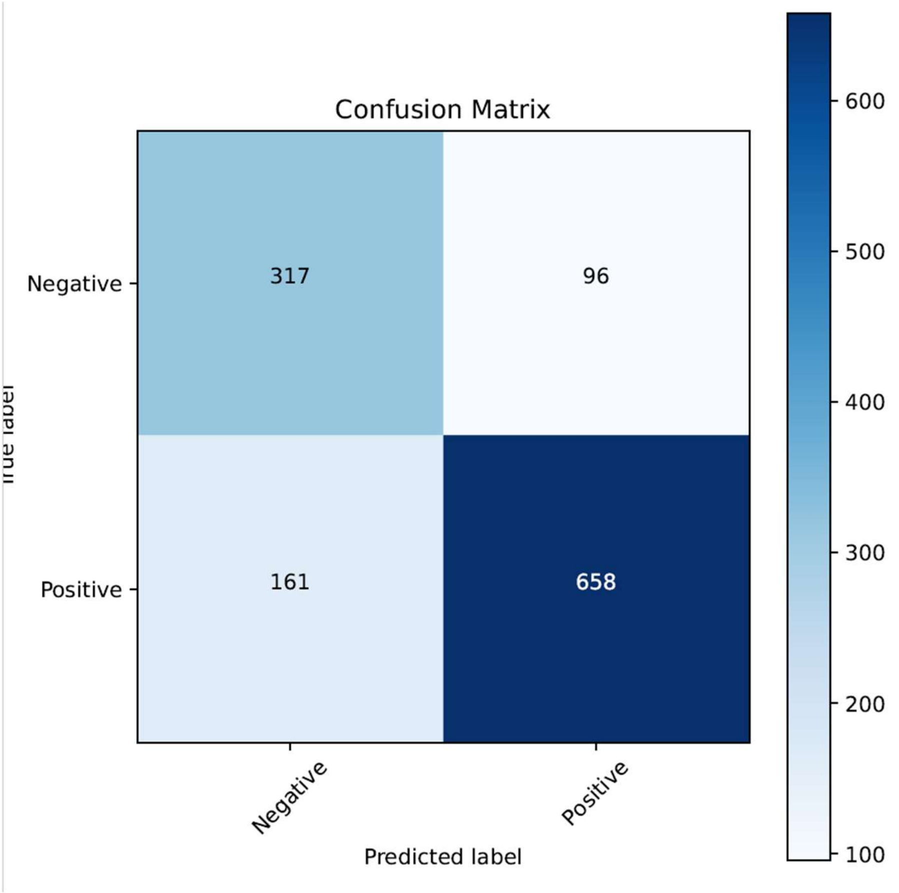

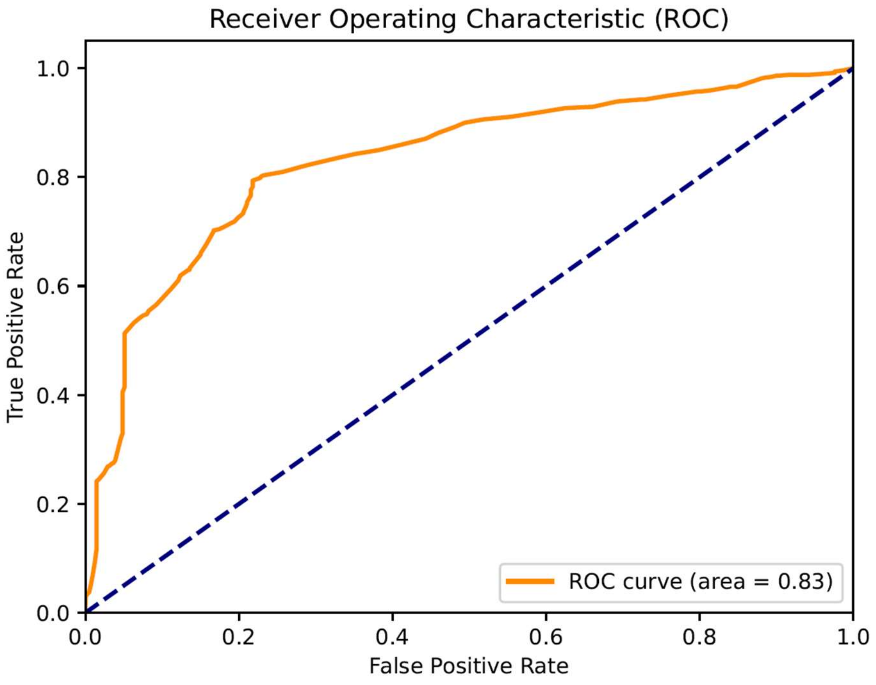

3.2. Extra tree

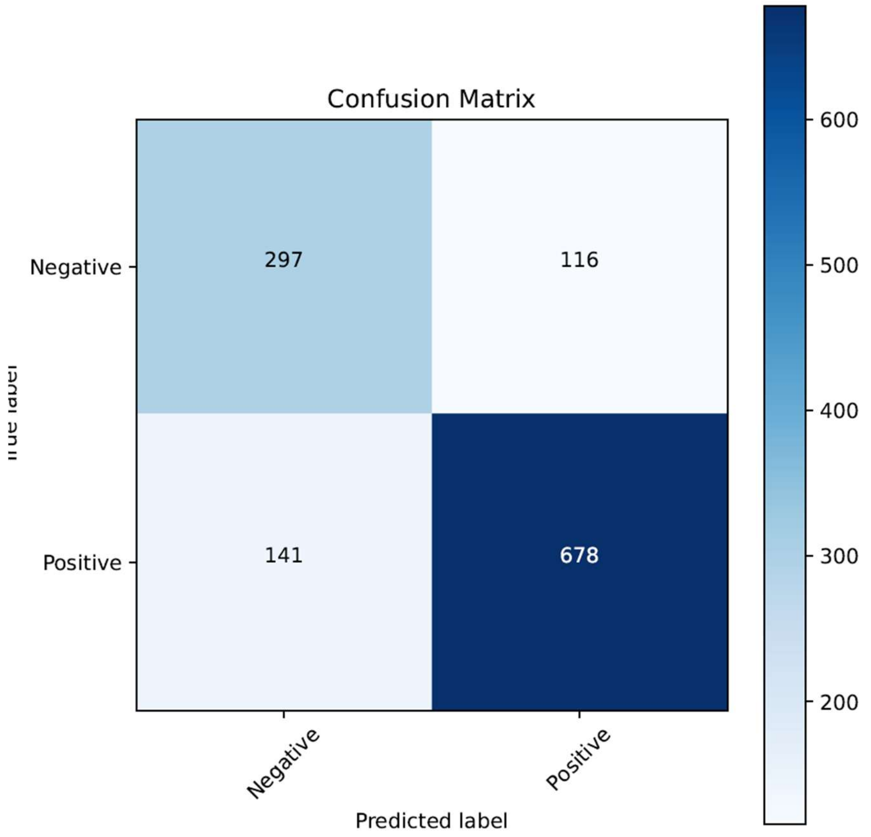

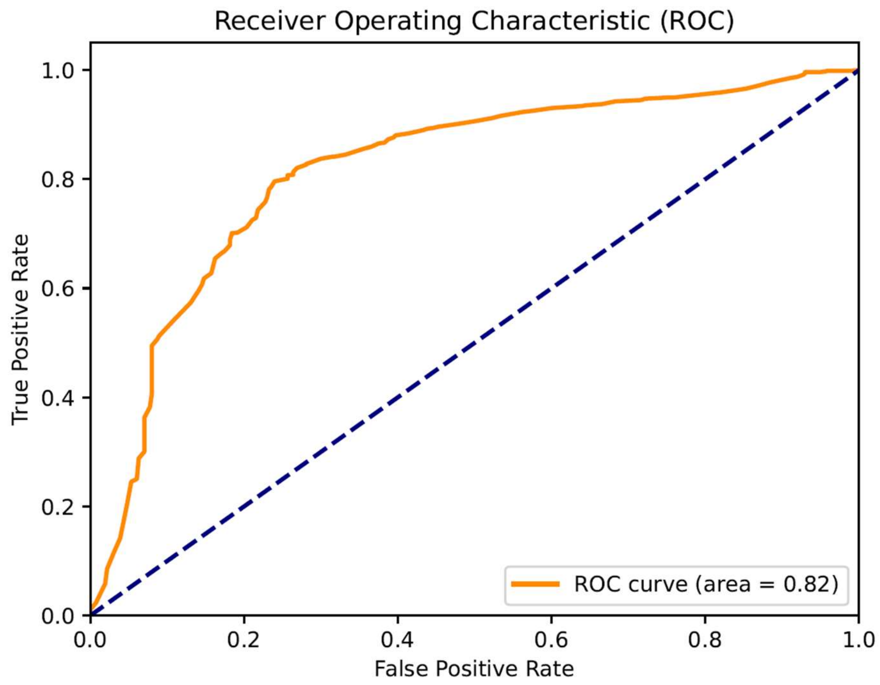

3.3. Extra Trees

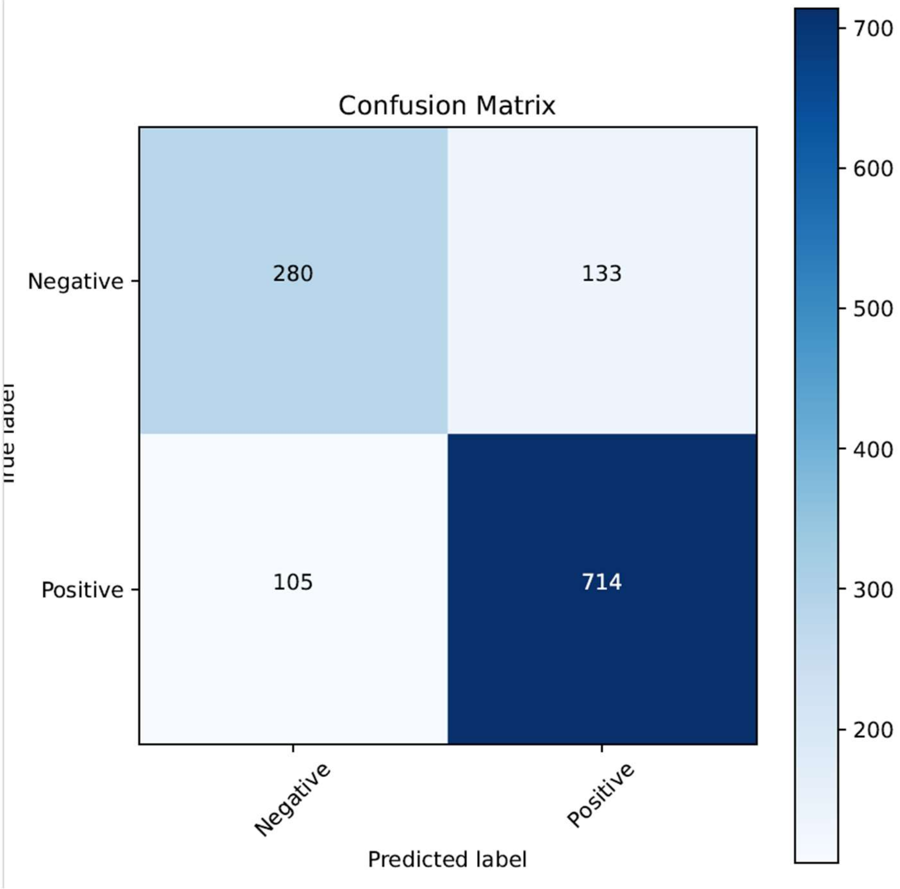

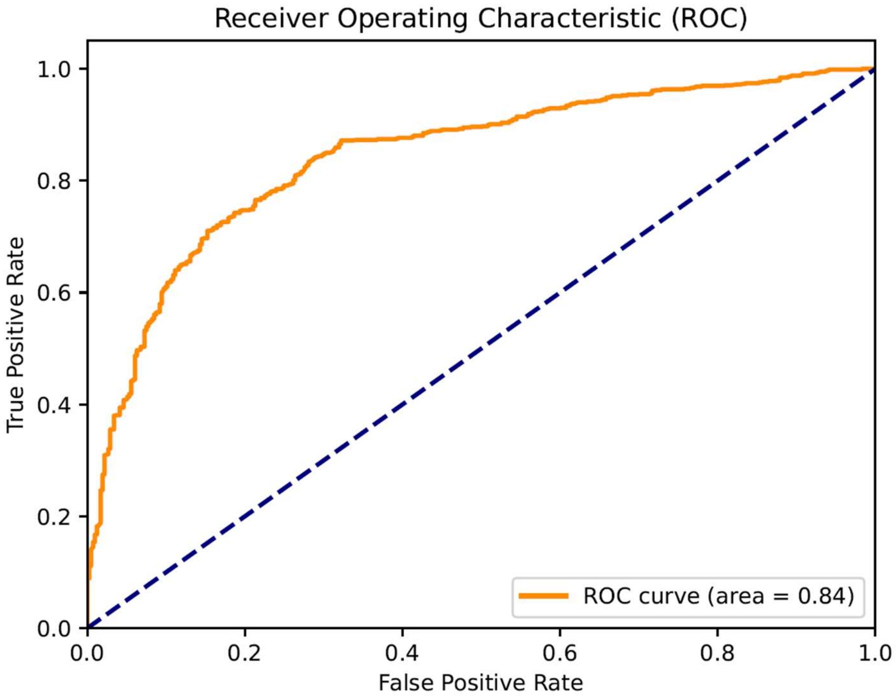

3.4. Gradient Boosting

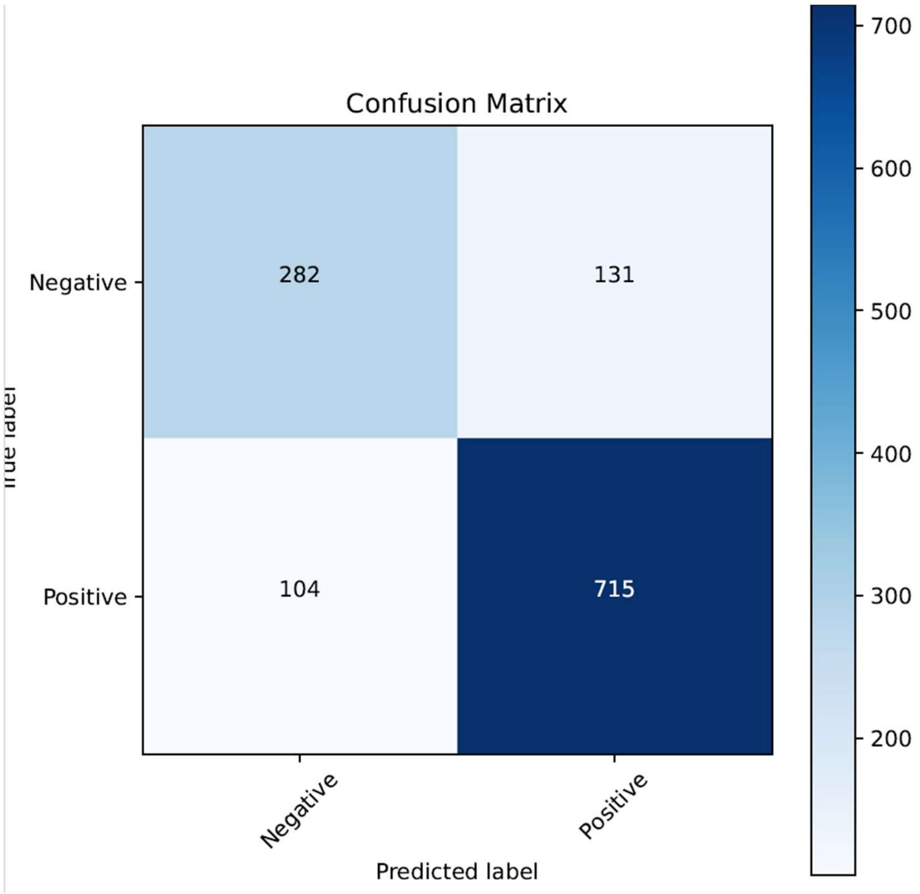

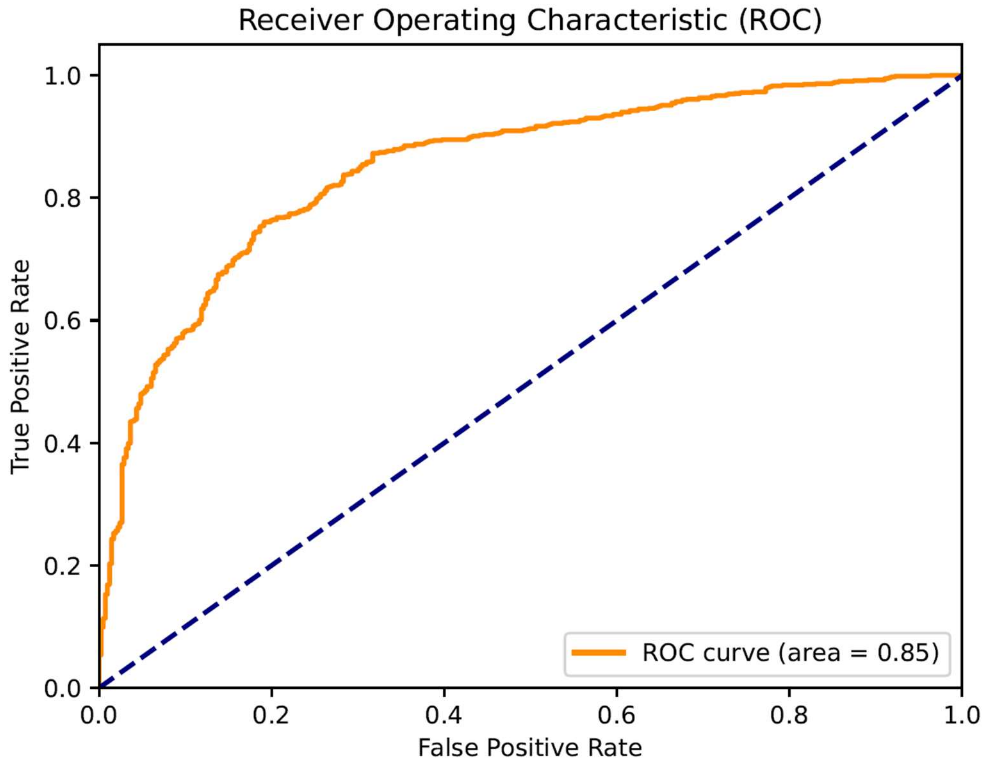

3.5. KNN

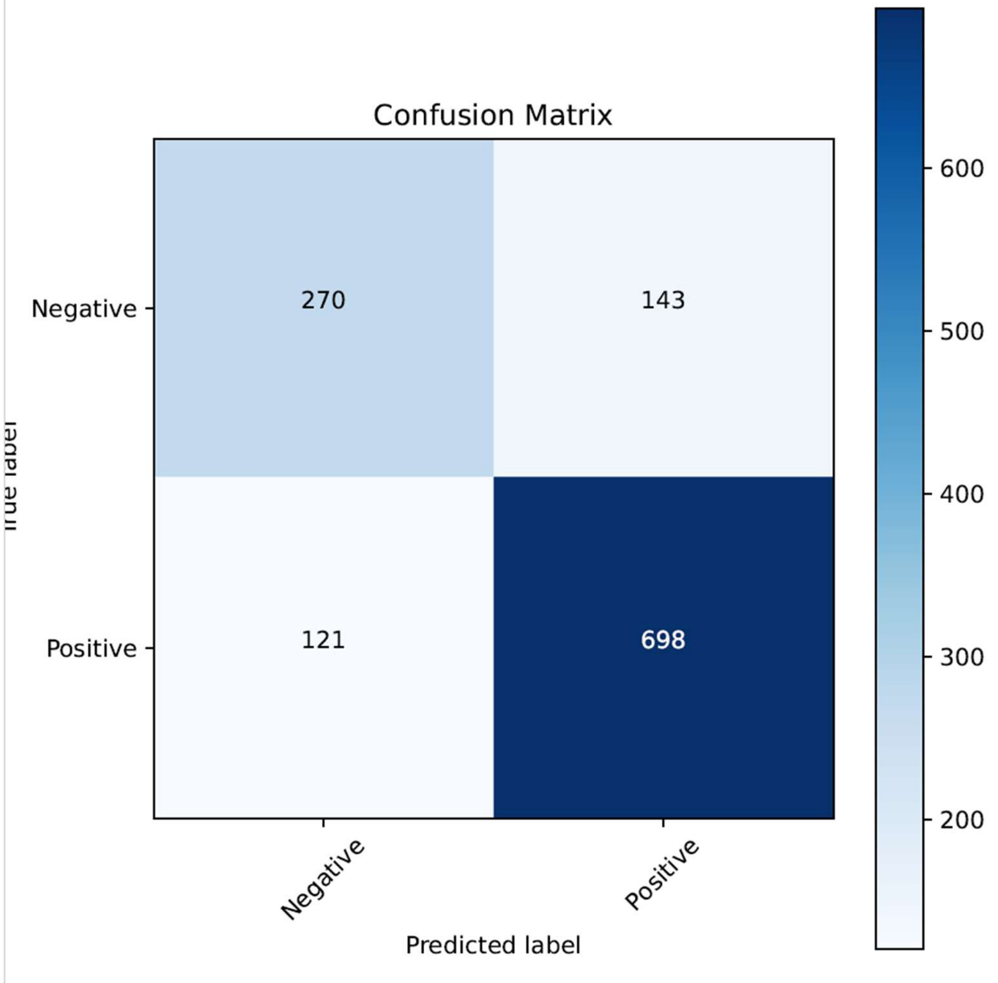

Confusion Matrix
Negative
Positive
270
143
121
698
True label
Negative
Positive
Predicted label
600
500
400
300
200

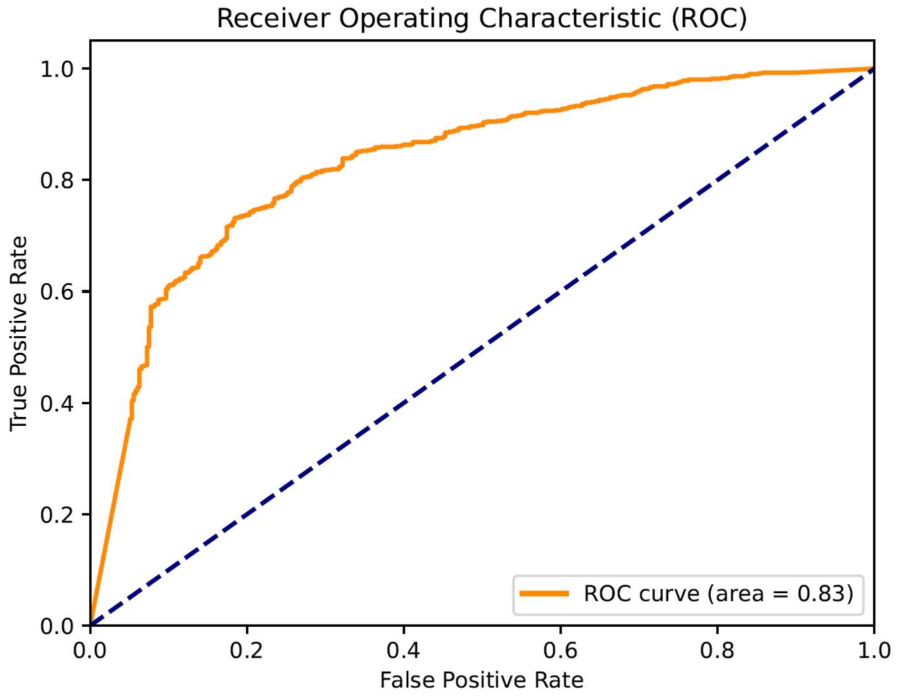

Receiver Operating Characteristic (ROC)
1.0
0.8
0.6
0.4
0.2
0.0
True Positive Rate
ROC curve (area = 0.83)
0.0
0.2
0.4
0.6
0.8
1.0
False Positive Rate

3.6. LightGBM

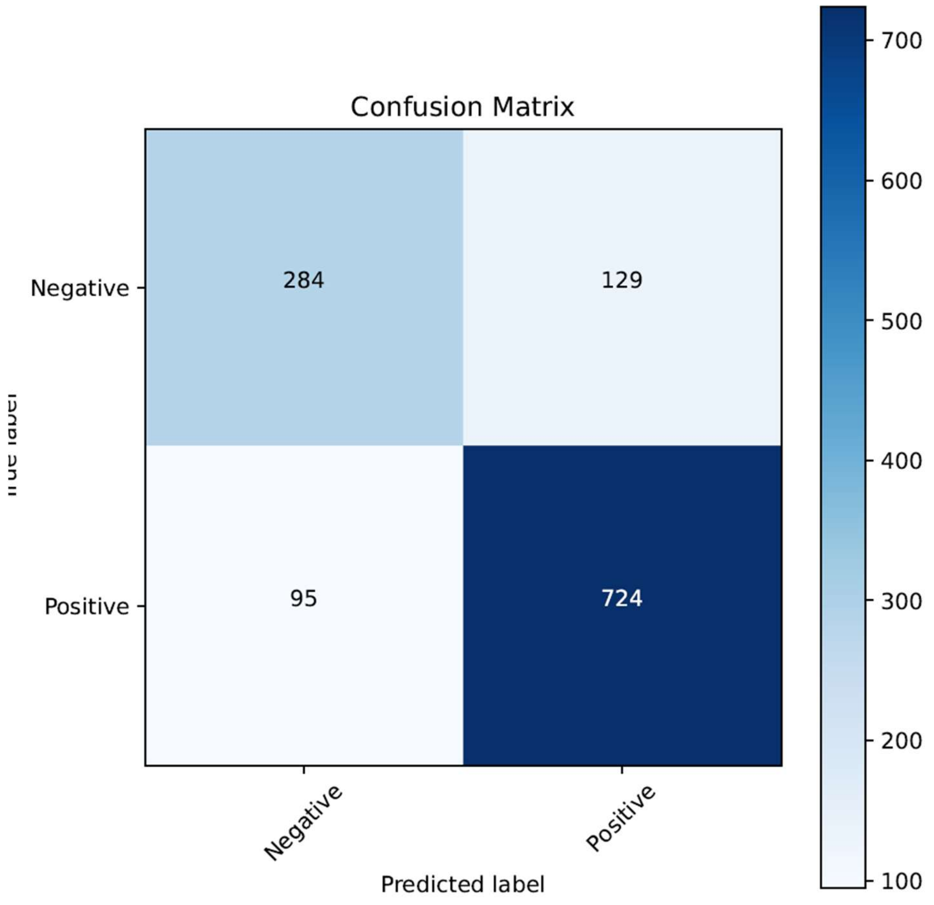

Confusion Matrix
Negative
Positive
284
129
95
724
Predicted label
700
600
500
400
300
200
100

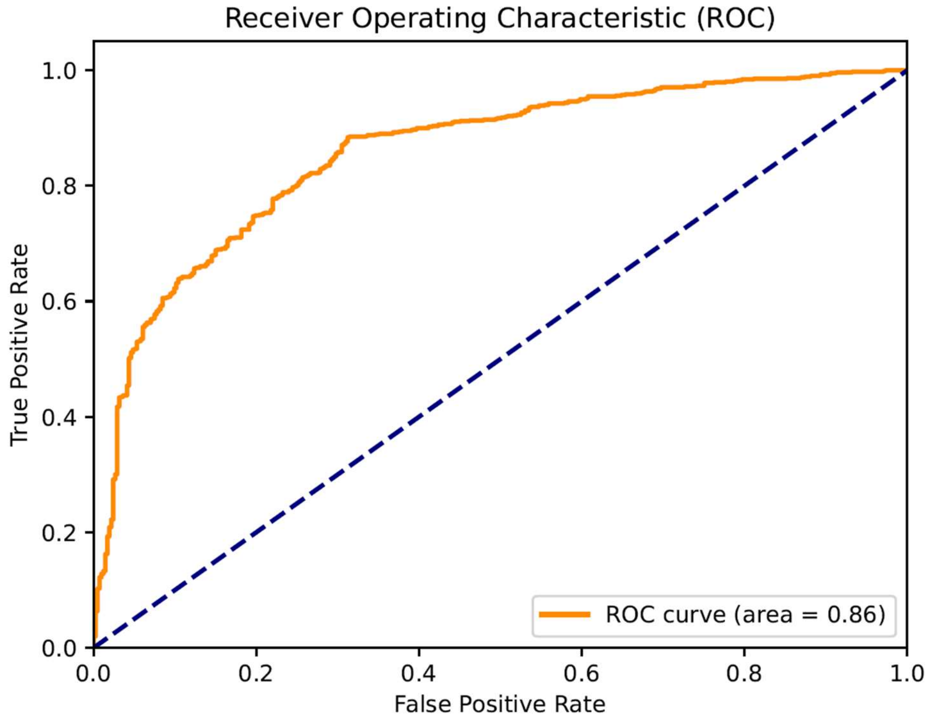

Receiver Operating Characteristic (ROC)
True Positive Rate
False Positive Rate
ROC curve (area = 0.86)
0.0
0.2
0.4
0.6
0.8
1.0

3.7. Logistic Regression

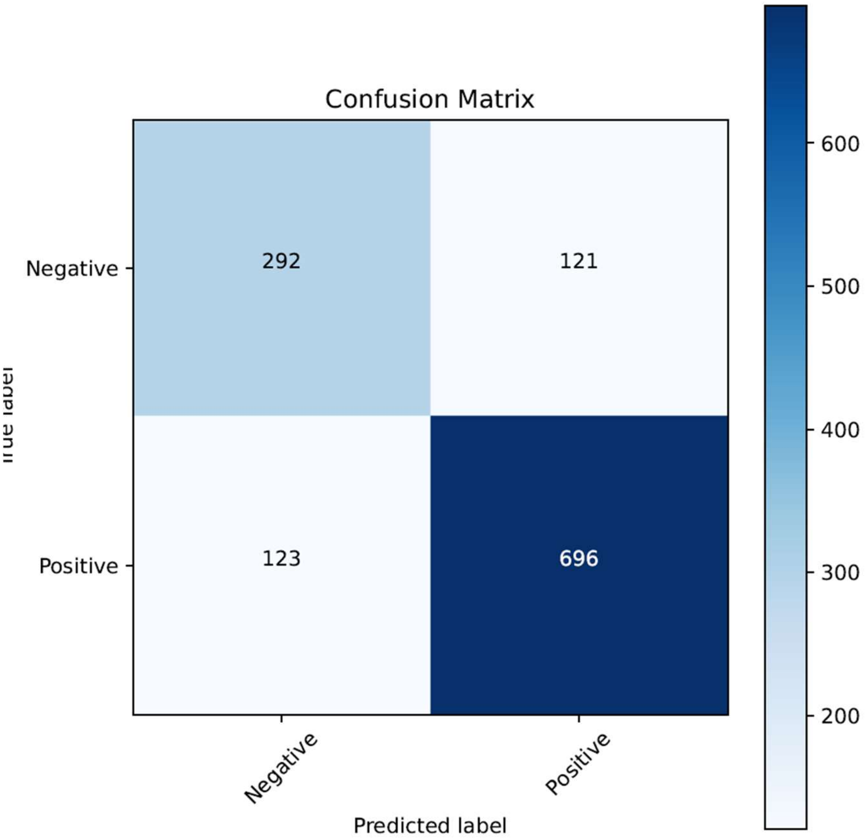

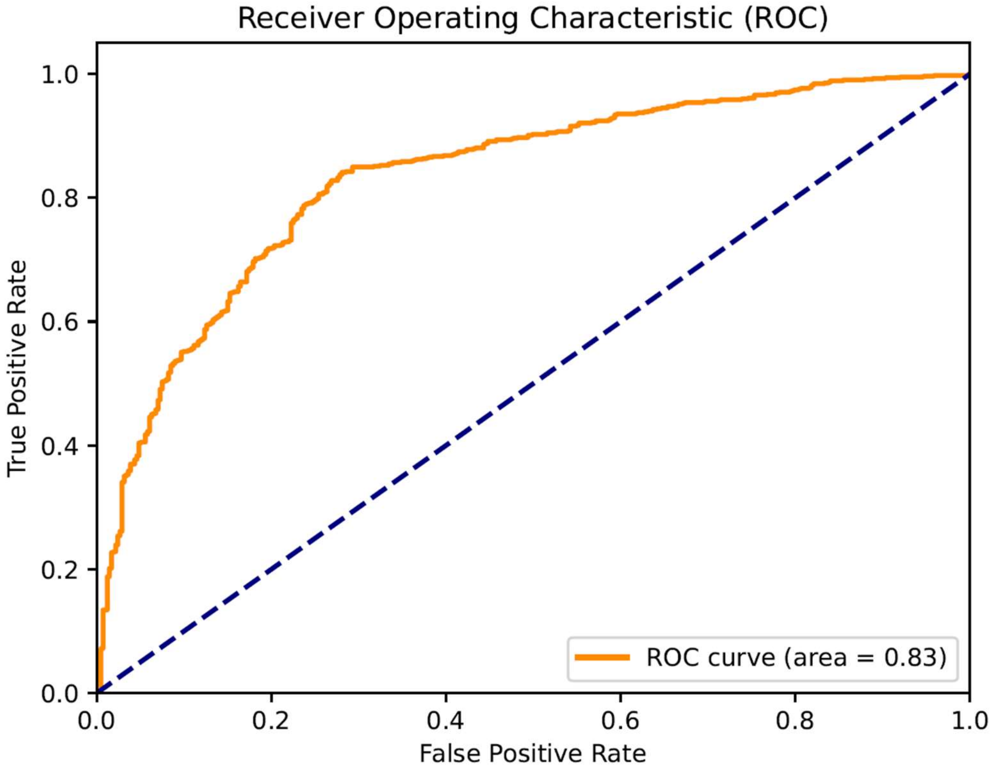

3.8. NuSVM

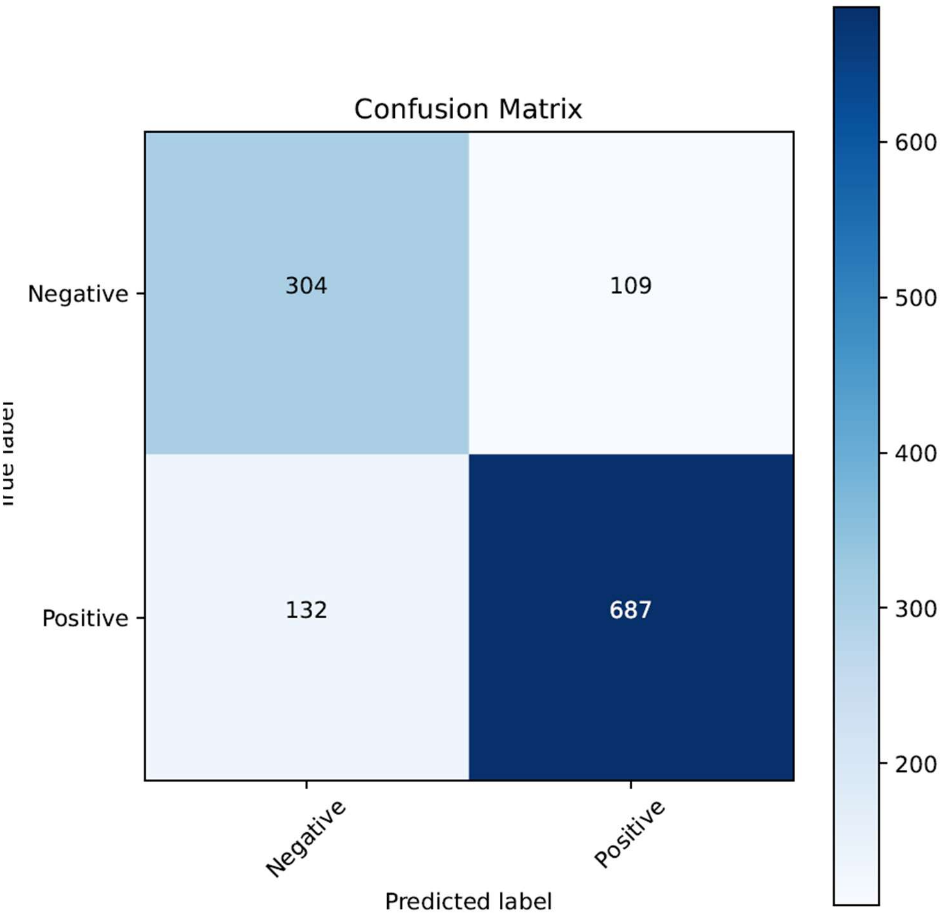

Confusion Matrix
Negative
Positive
304
109
132
687
True label
Negative
Positive
Predicted label
600
500
400
300
200

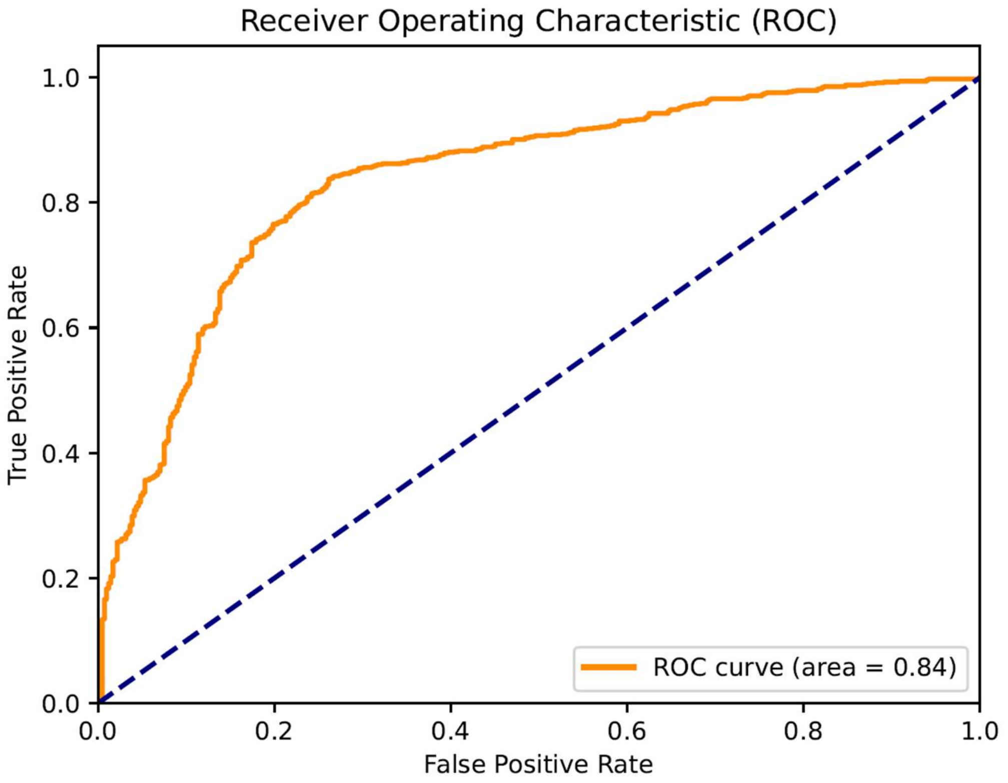

Receiver Operating Characteristic (ROC)
1.0
0.8
0.6
0.4
0.2
0.0
True Positive Rate
ROC curve (area = 0.84)
0.0
0.2
0.4
0.6
0.8
1.0
False Positive Rate

3.9. Random Forest

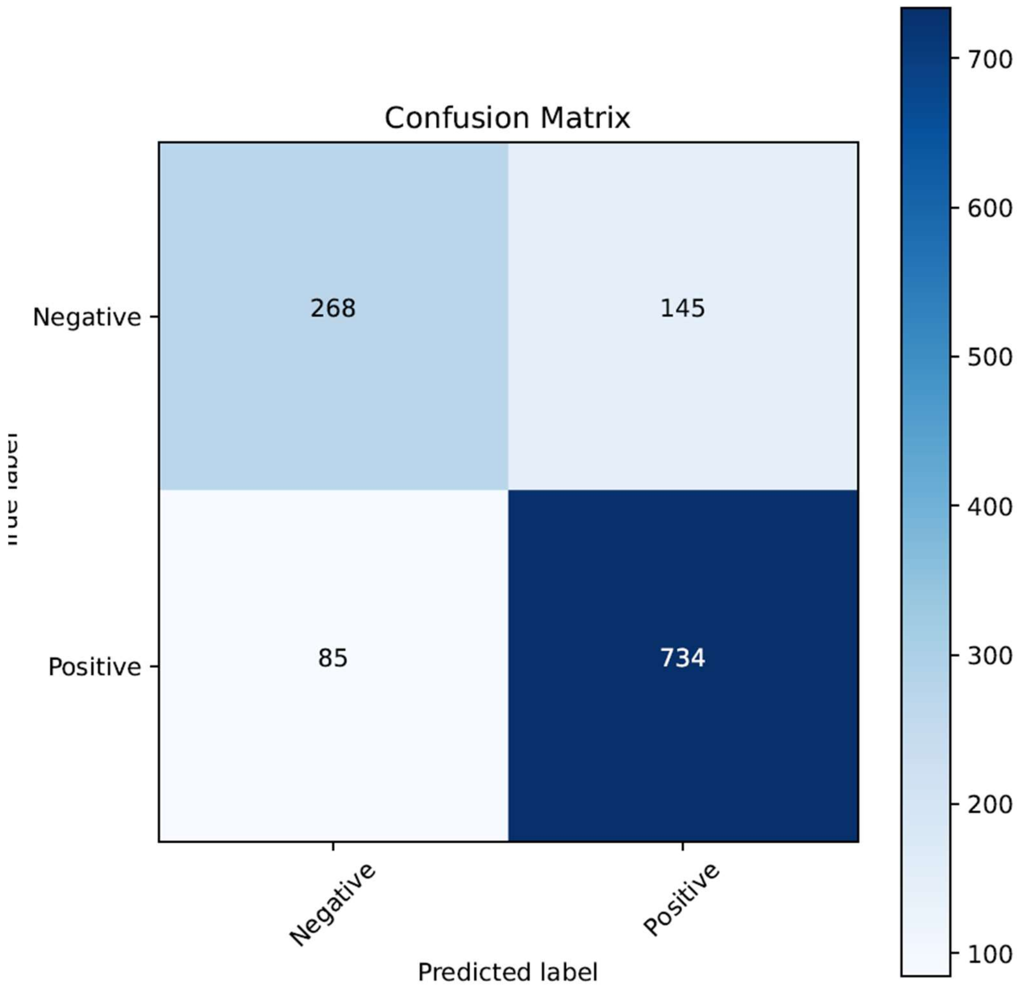

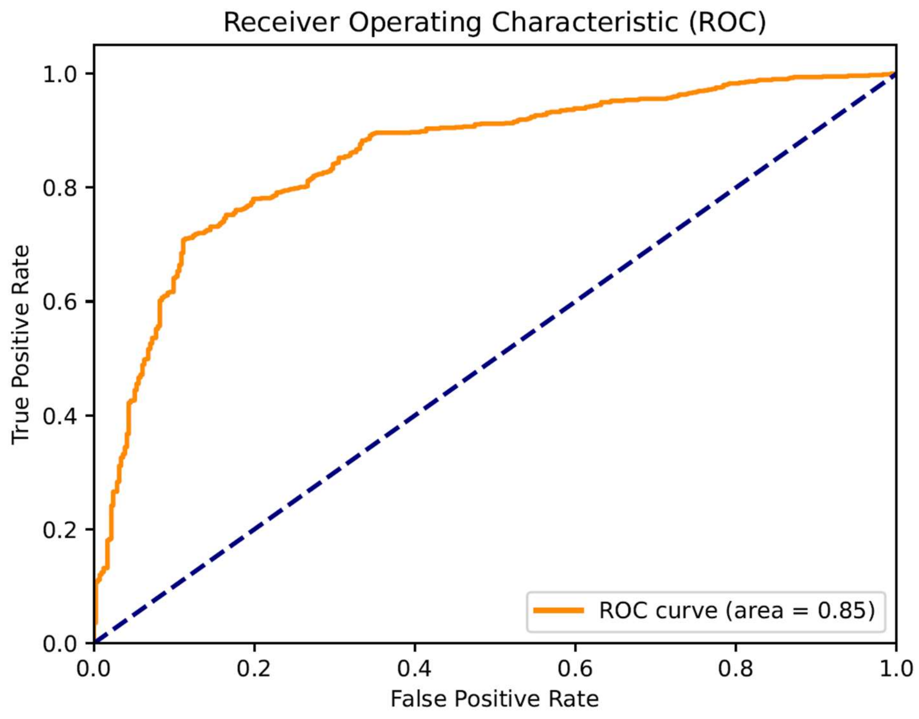

3.10. XgBoost

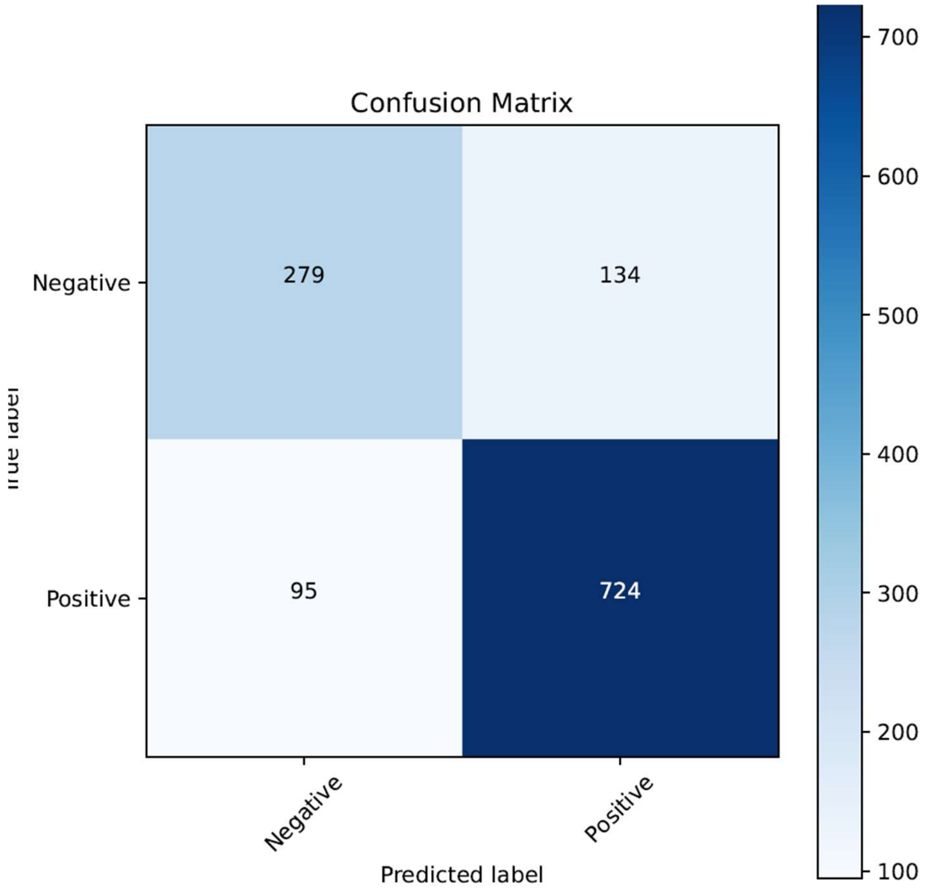
Confusion Matrix
Negative
Positive
279
134
95
724
Negative
Positive
Predicted label
700
600
500
400
300
200
100

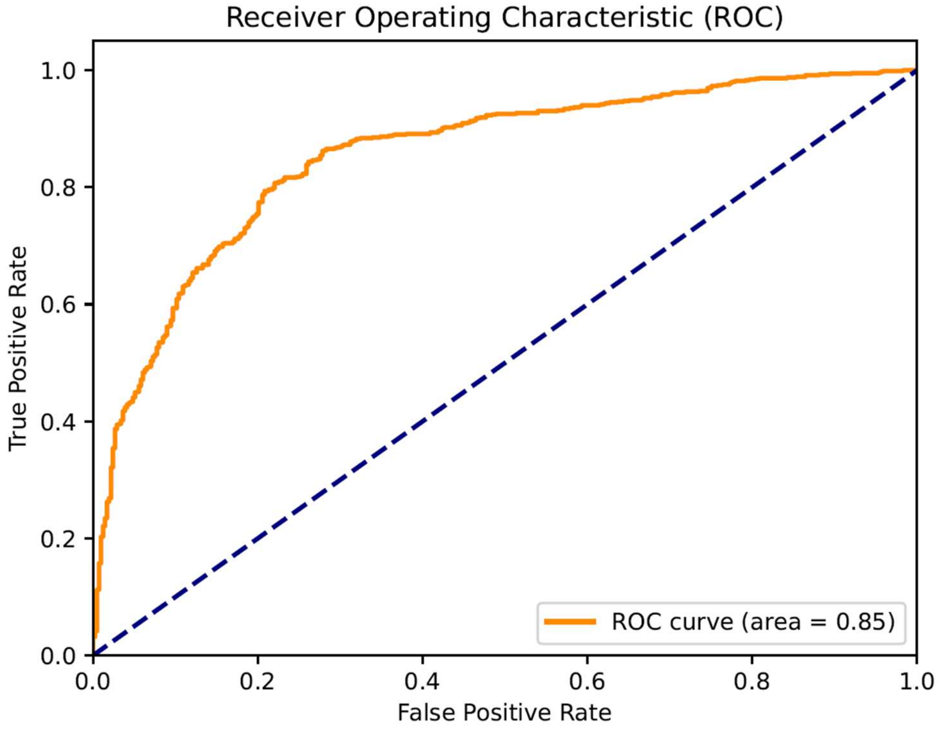
Receiver Operating Characteristic (ROC)
True Positive Rate
False Positive Rate
0.0
0.2
0.4
0.6
0.8
1.0
ROC curve (area = 0.85)

4. Optimization of classifier parameters and feature selection using the Naked Mole-Rat Algorithm with oversampling techniques.

4.1. Decision tree

4.1.1. SMOTE

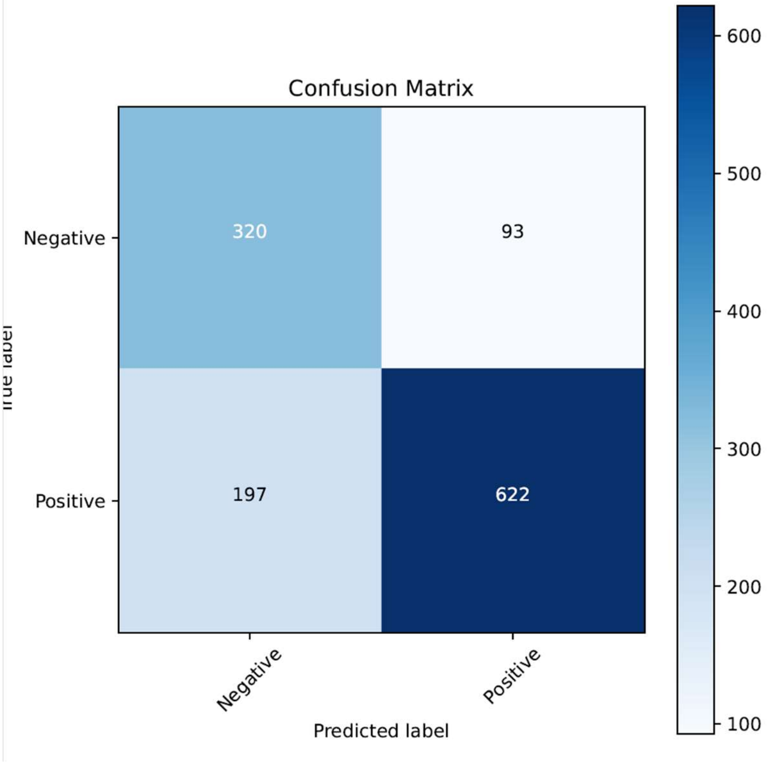

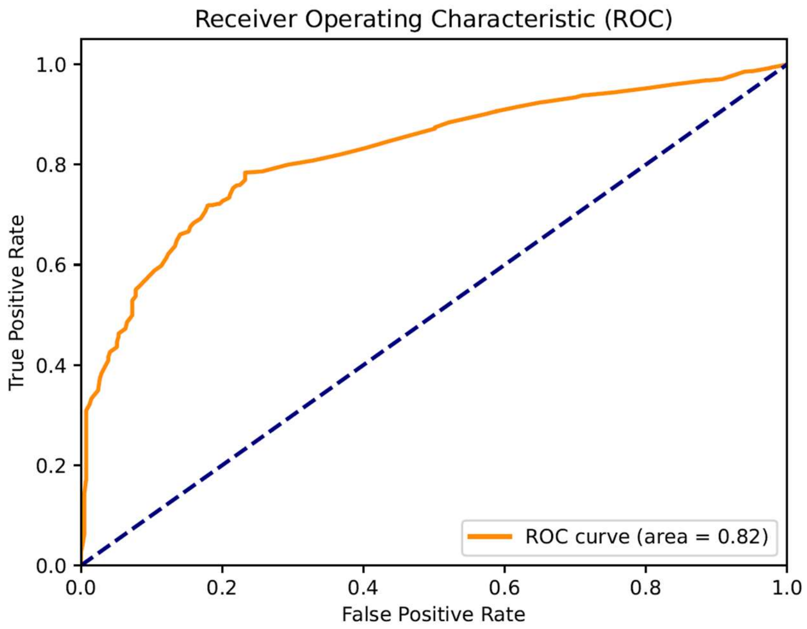

4.1.2. ADASYN

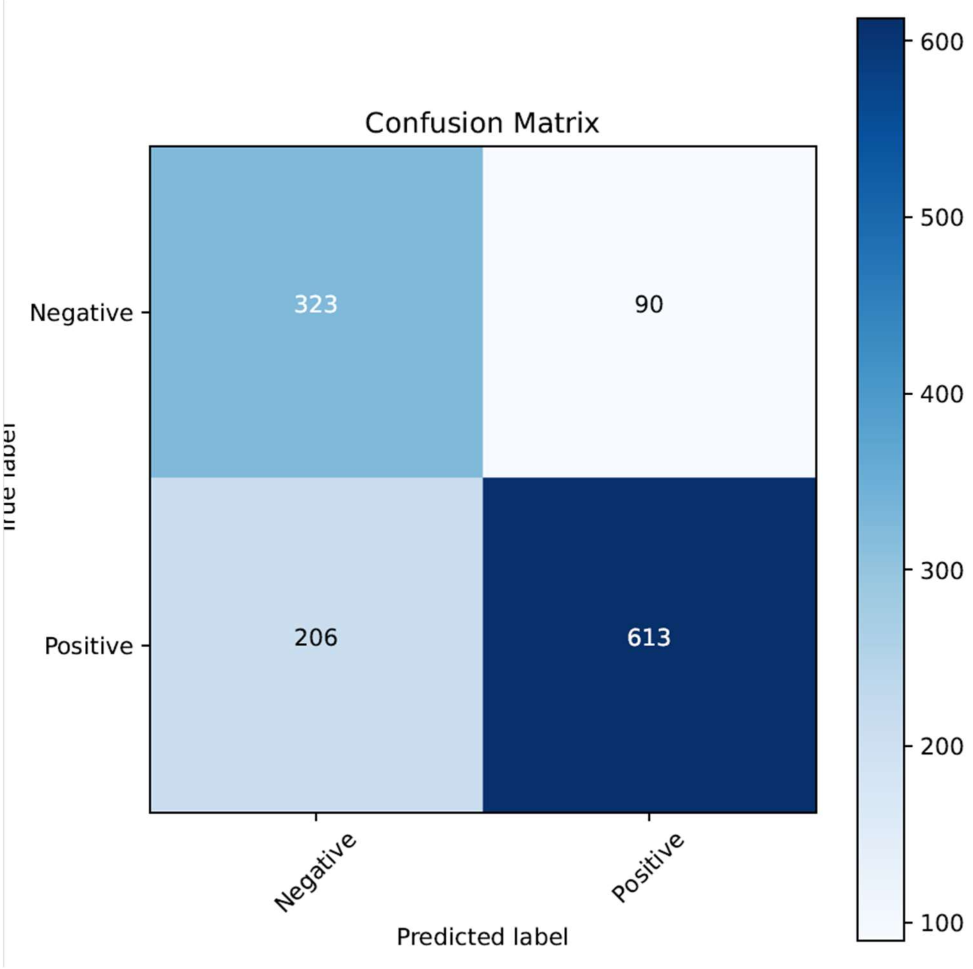
Confusion Matrix
Negative
Positive
323
90
206
613
True label
Negative
Positive
Predicted label
600
500
400
300
200
100

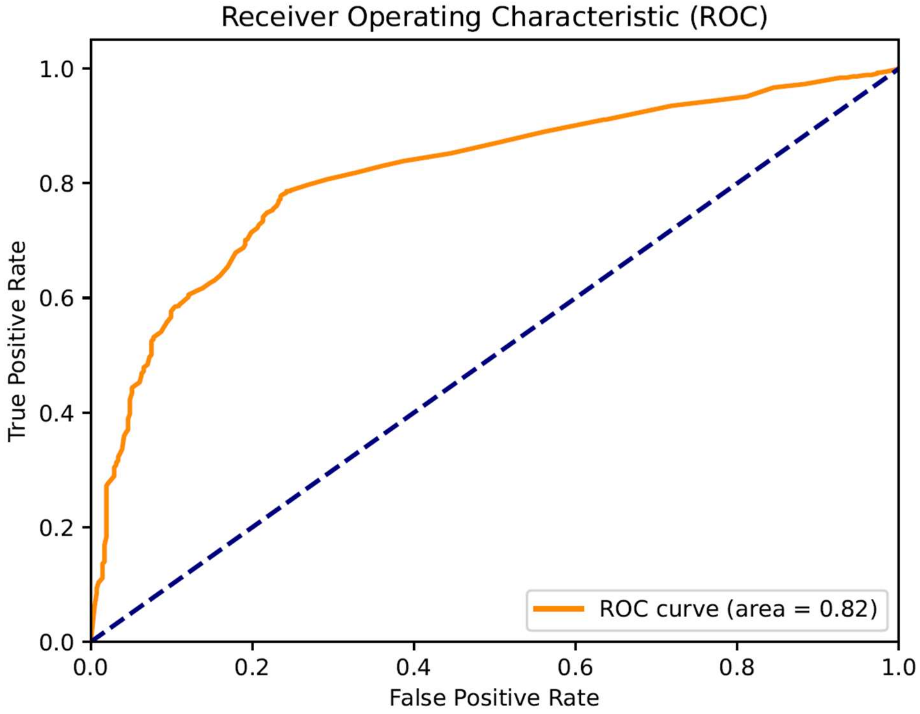
Receiver Operating Characteristic (ROC)
1.0
0.8
0.6
0.4
0.2
0.0
True Positive Rate
0.0
0.2
0.4
0.6
0.8
1.0
False Positive Rate
ROC curve (area = 0.82)

4.2. Extra Tree

4.2.1. SMOTE

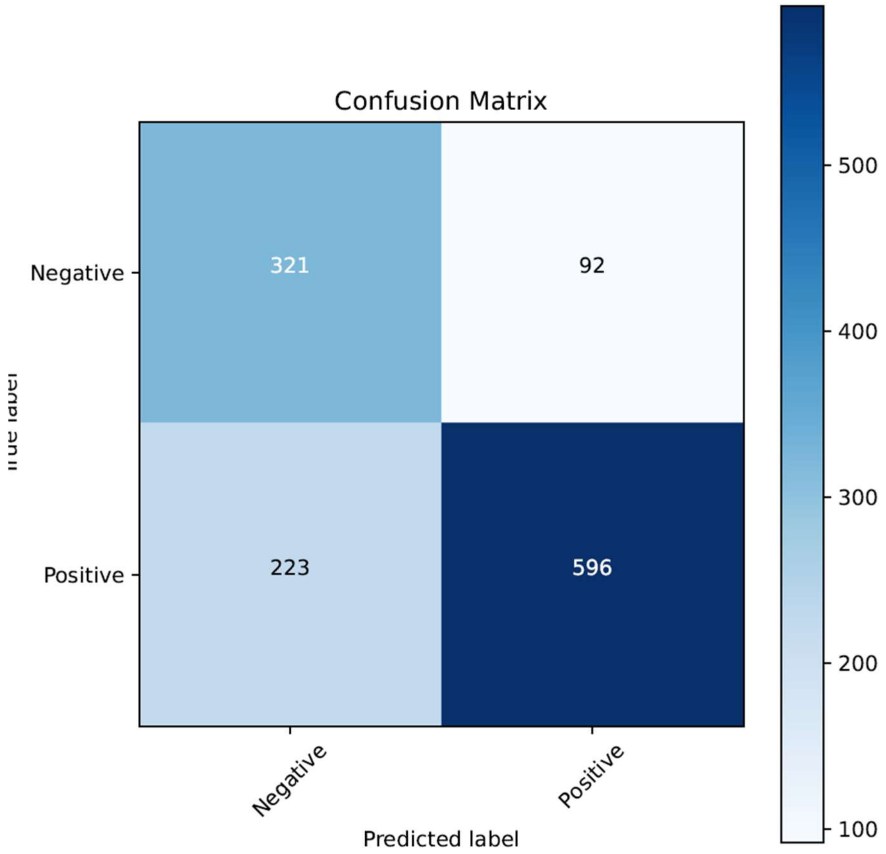

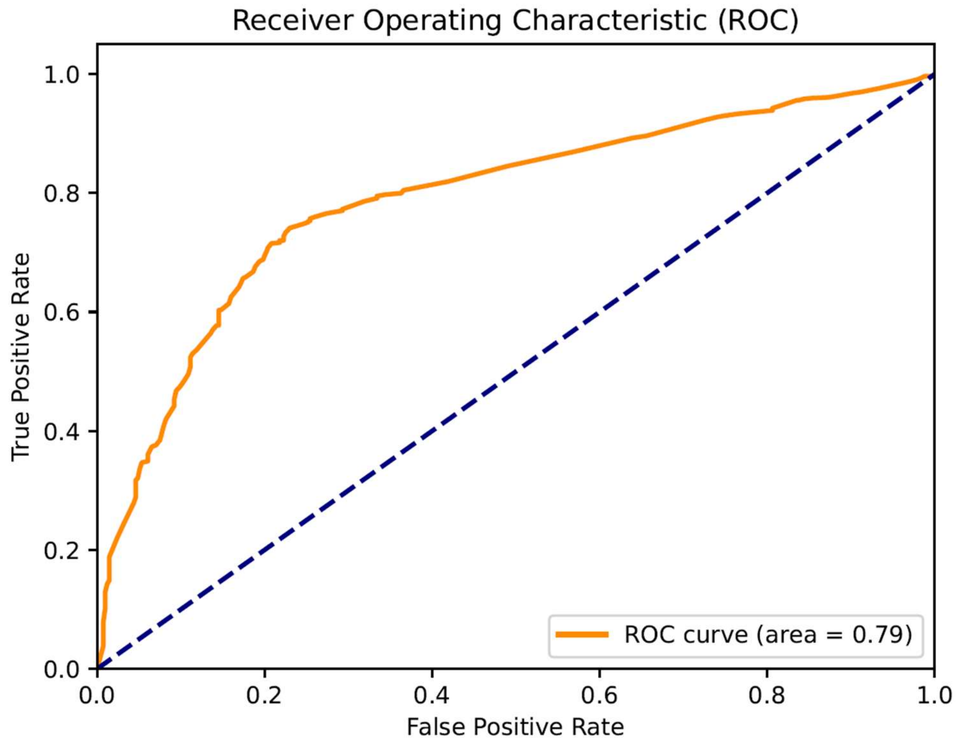

4.2.2. ADASYN

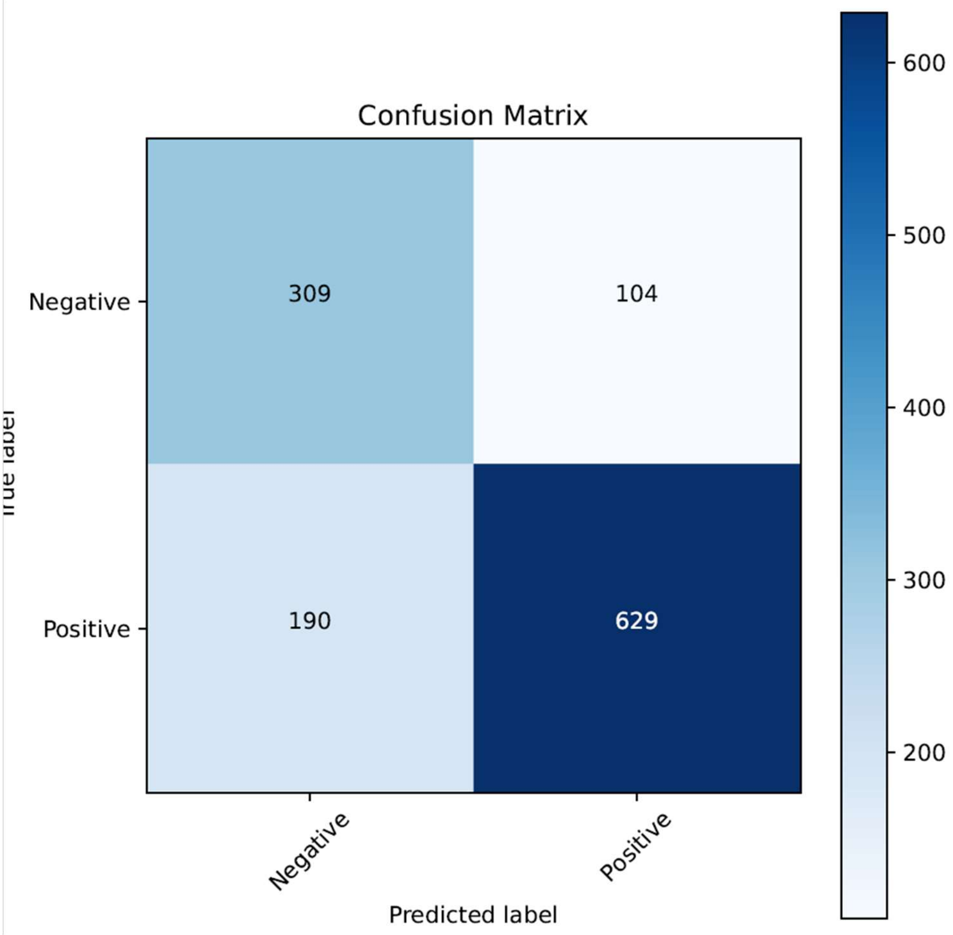

Confusion Matrix
Negative
Positive
309
104
190
629
True label
Predicted label
600
500
400
300
200

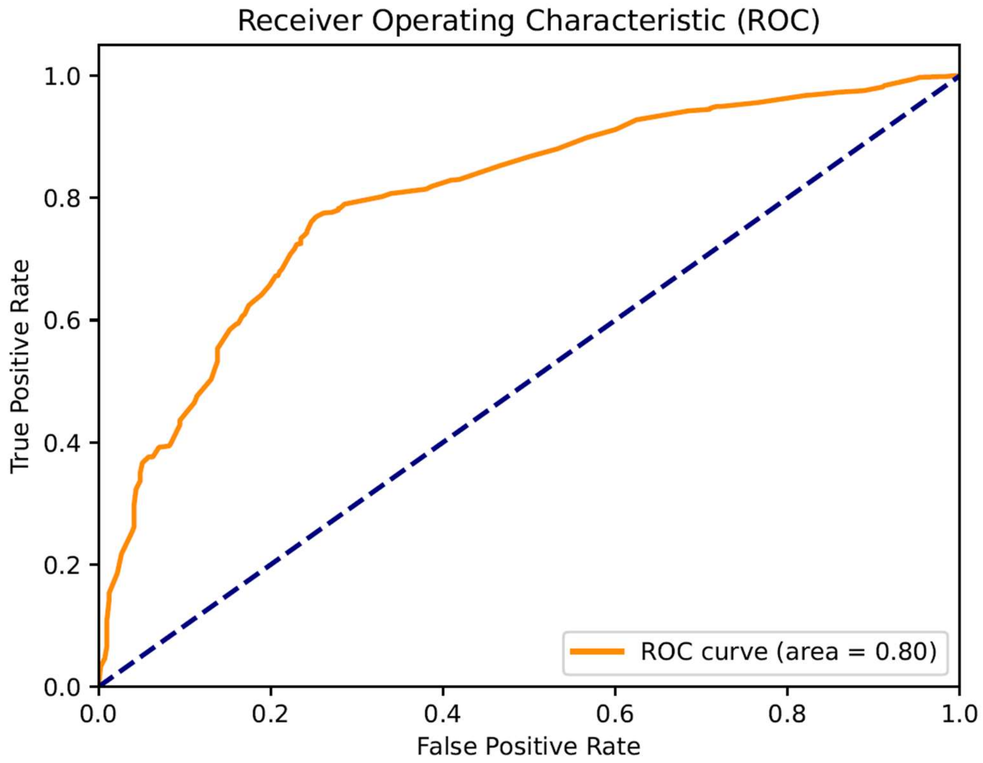

Receiver Operating Characteristic (ROC)
True Positive Rate
False Positive Rate
0.0
0.2
0.4
0.6
0.8
1.0
ROC curve (area = 0.80)

4.3. Extra Trees

4.3.1. SMOTE

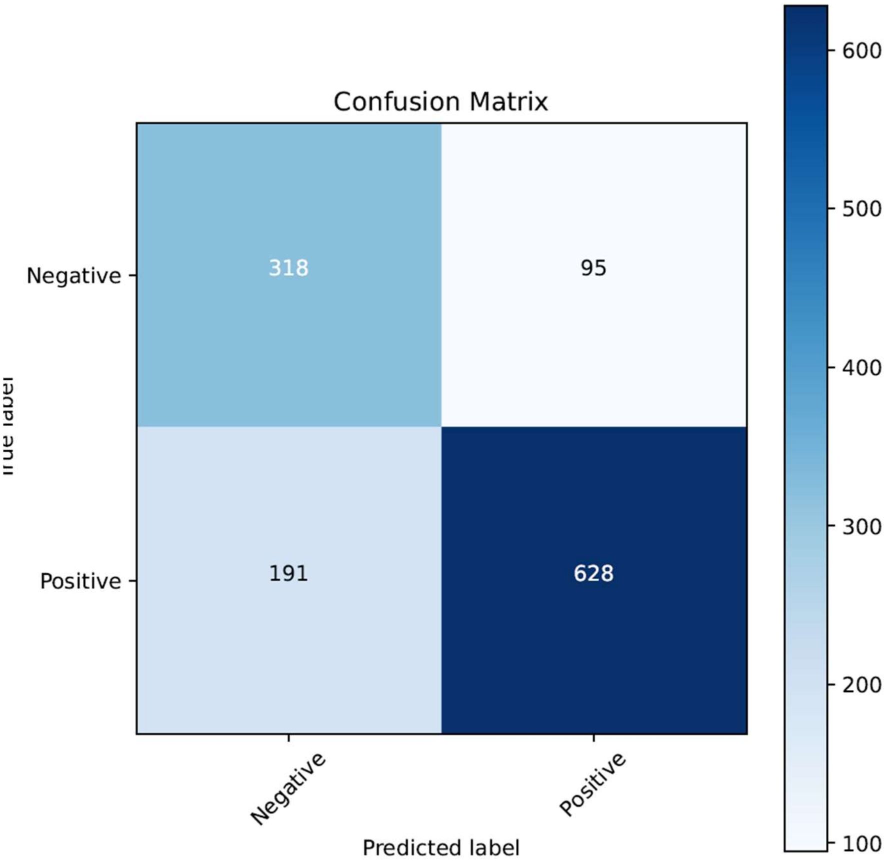

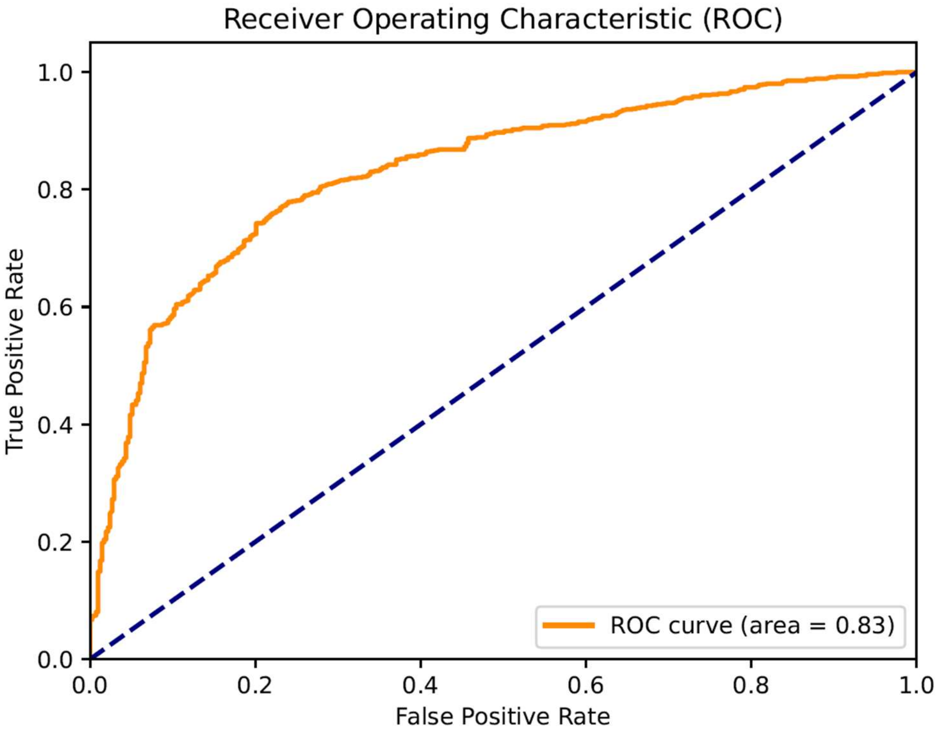

4.3.2. ADASYN

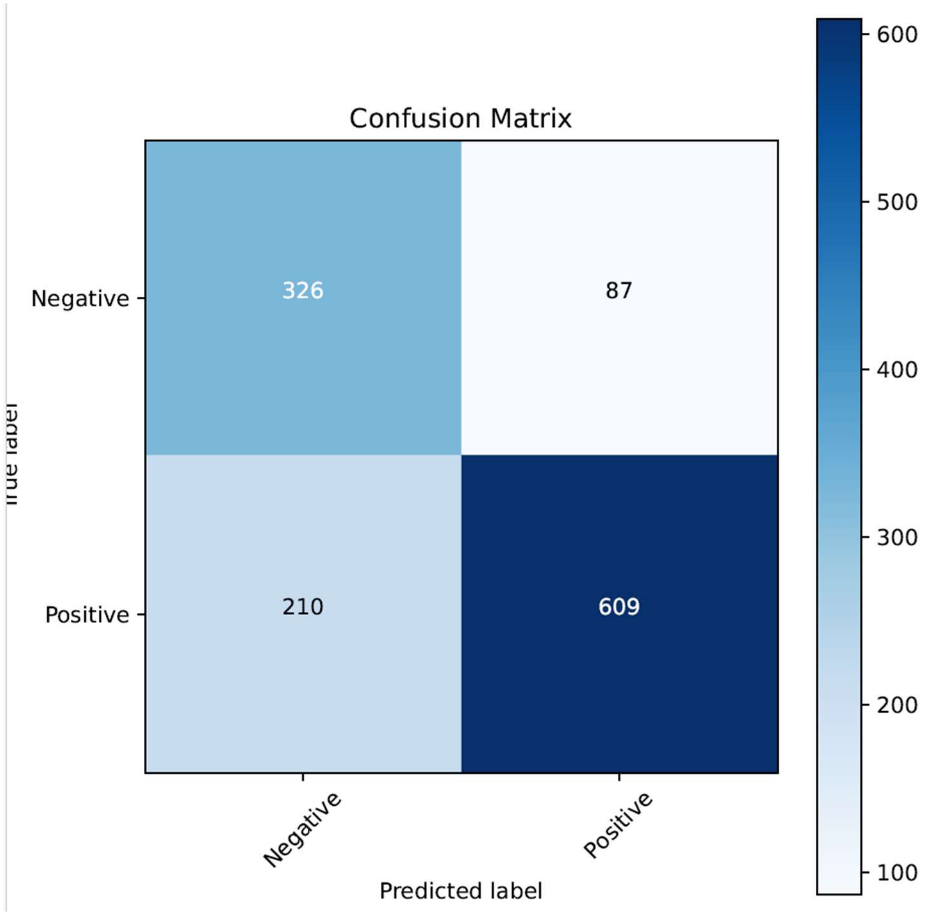

Confusion Matrix
Negative
Positive
326
87
210
609
True label
Predicted label
600
500
400
300
200
100

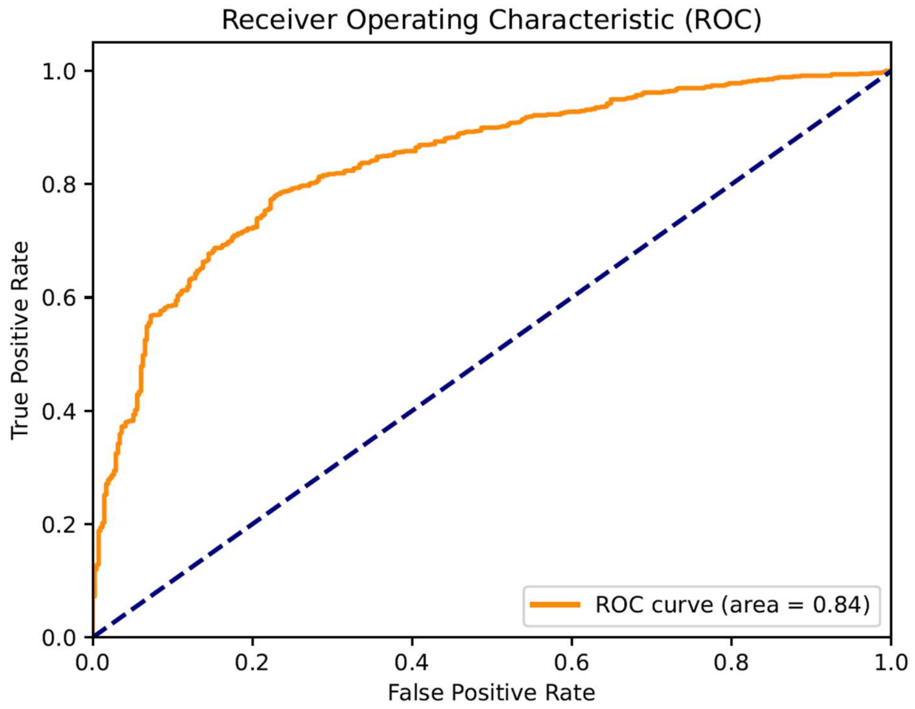

Receiver Operating Characteristic (ROC)
True Positive Rate
False Positive Rate
0.0
0.2
0.4
0.6
0.8
1.0
ROC curve (area = 0.84)

4.4. Gradient Boosting

4.4.1. SMOTE

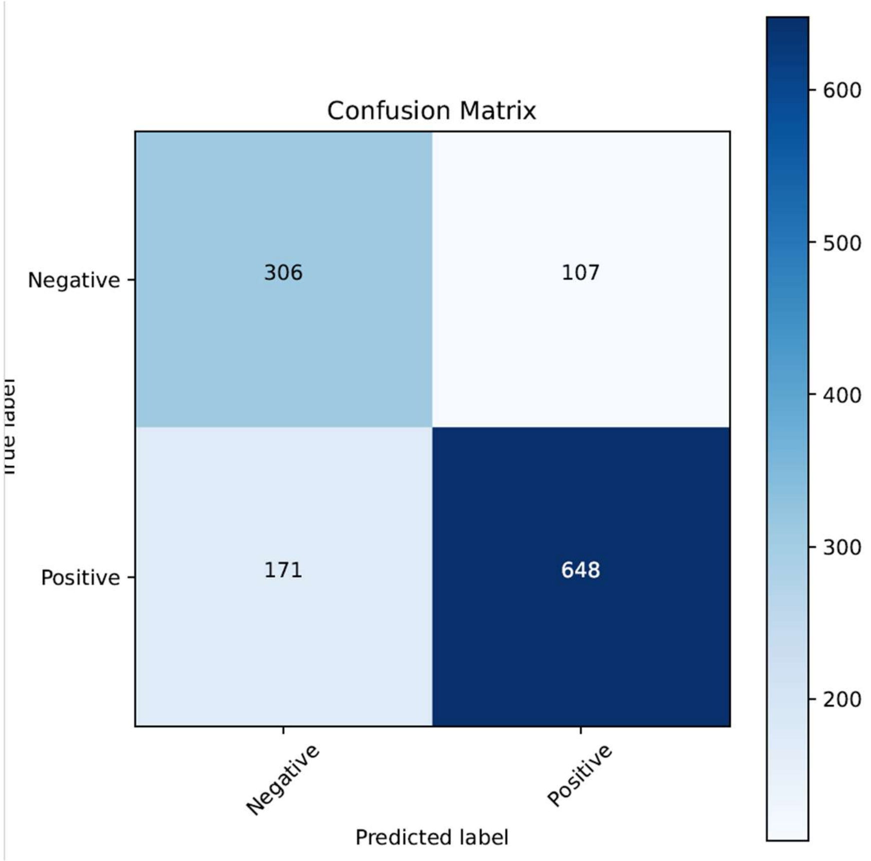

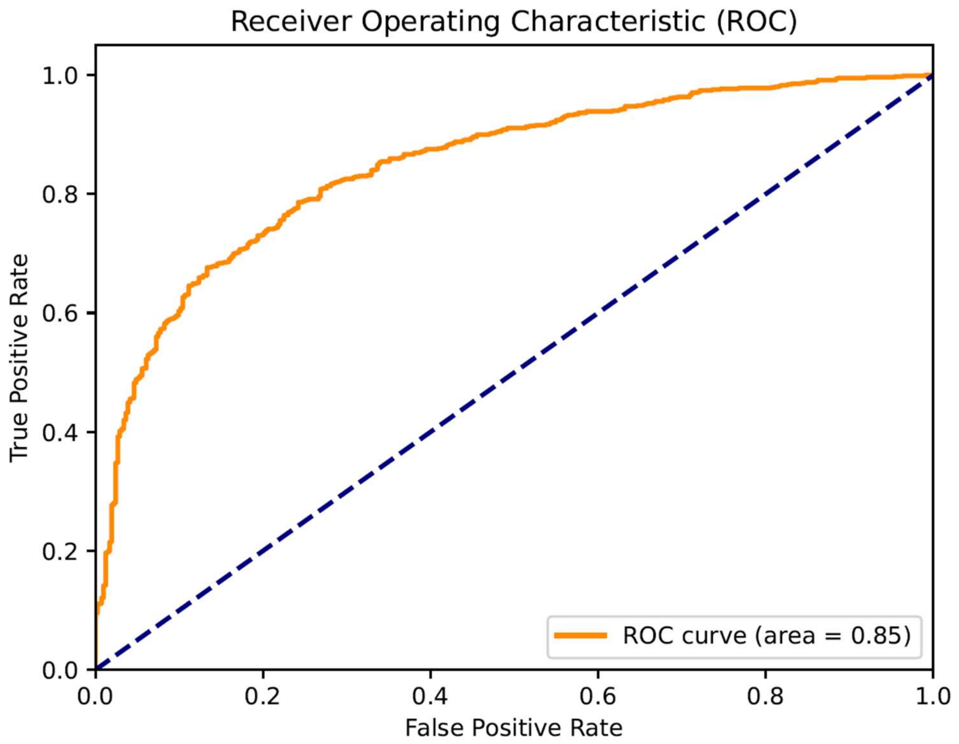

4.4.2. ADASYN

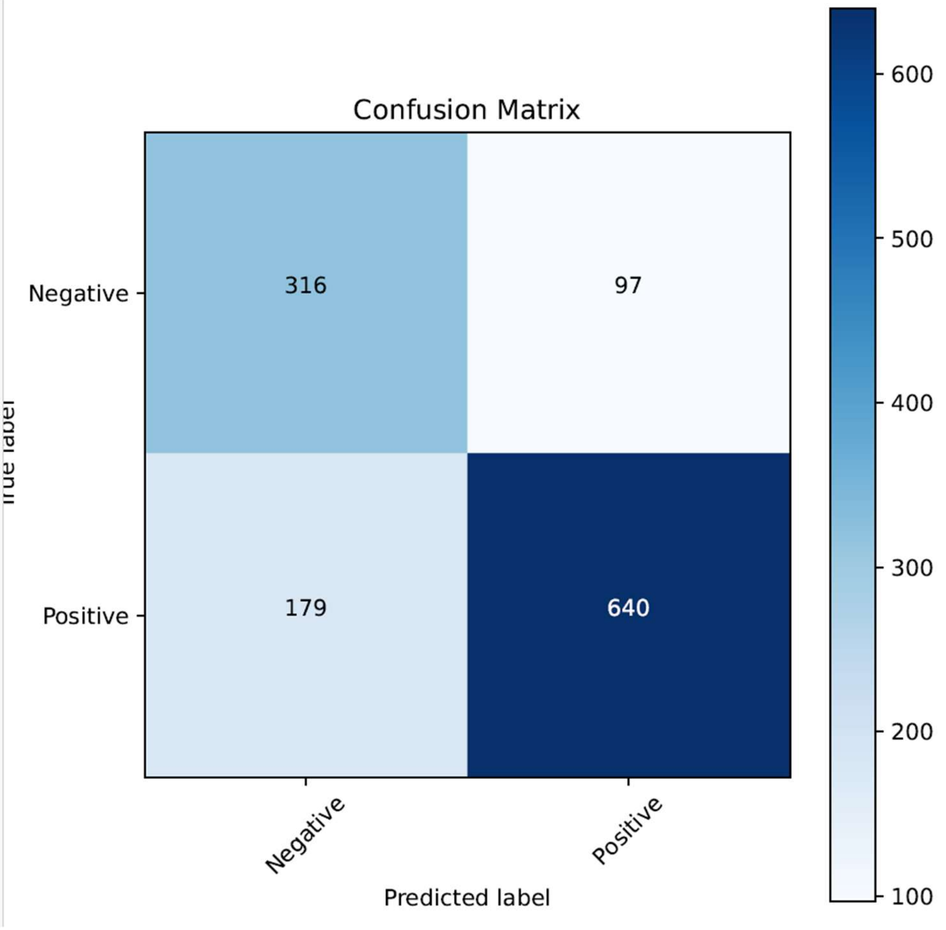
Confusion Matrix
Negative
Positive
316
97
179
640
True label
Negative
Positive
Predicted label
600
500
400
300
200
100

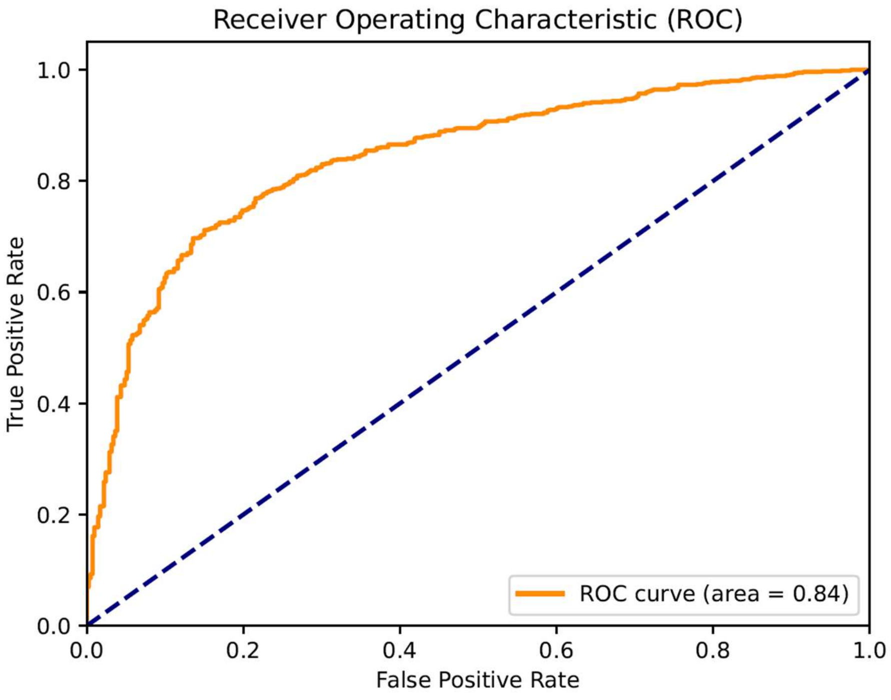
Receiver Operating Characteristic (ROC)
1.0
0.8
0.6
0.4
0.2
0.0
True Positive Rate
0.0
0.2
0.4
0.6
0.8
1.0
False Positive Rate
ROC curve (area = 0.84)

4.5. KNN

4.5.1. SMOTE

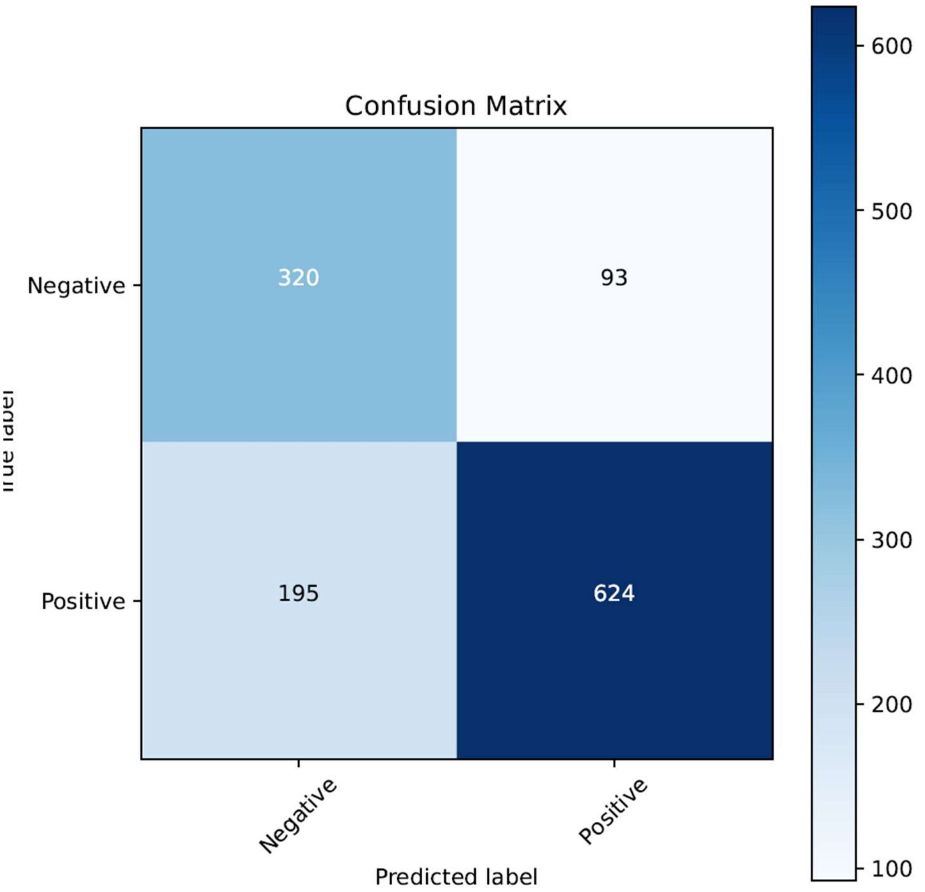

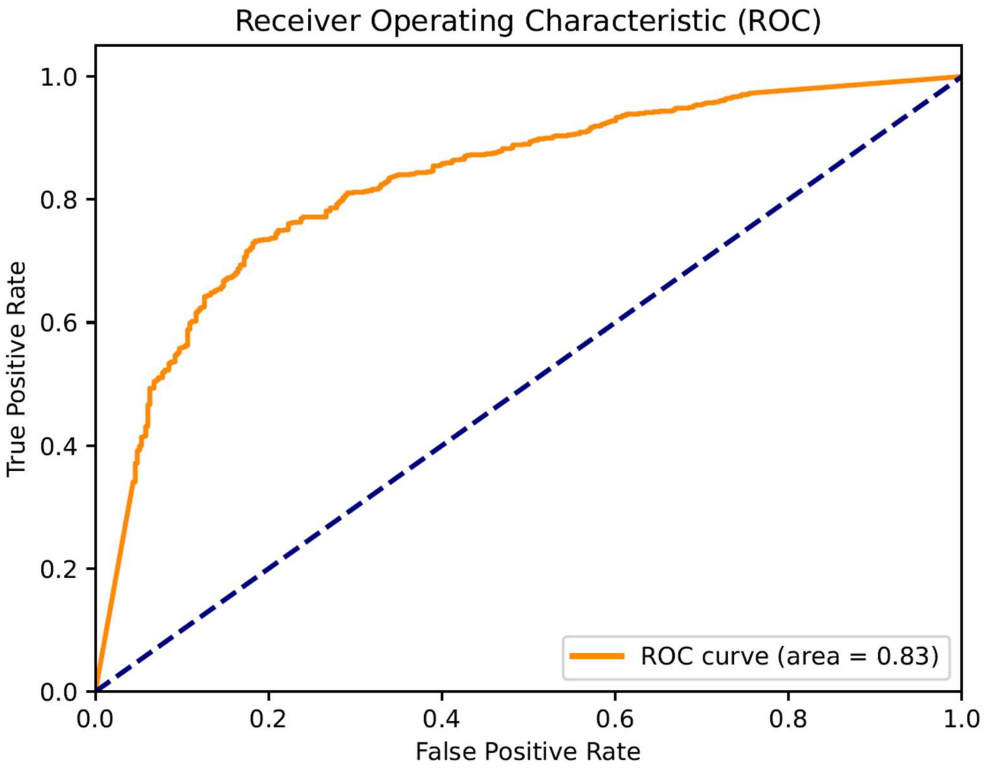

4.5.2. ADASYN

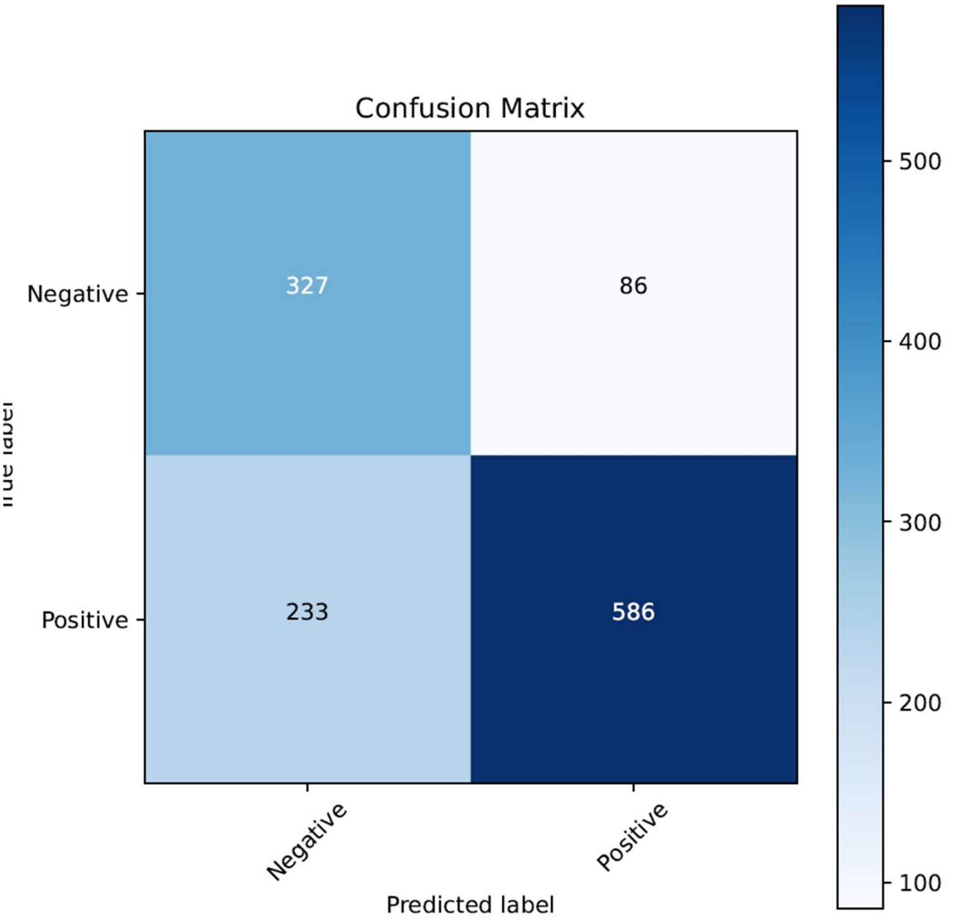

Confusion Matrix
Negative
Positive
327
86
233
586
Negative
Positive
Predicted label
500
400
300
200
100

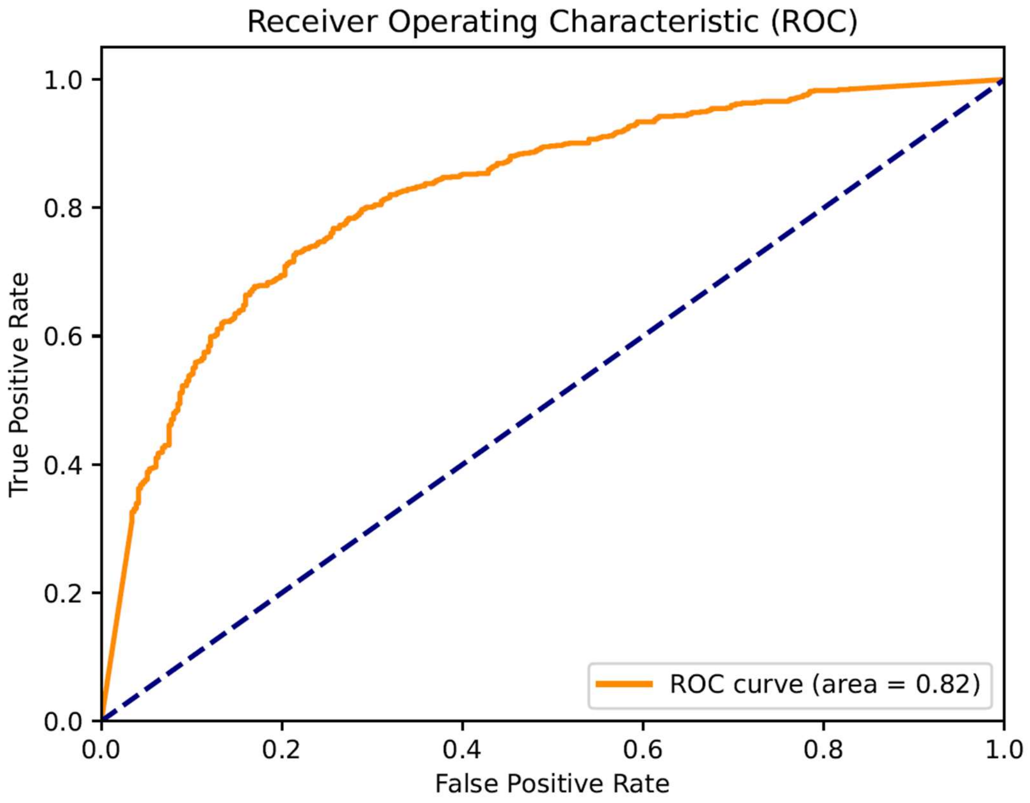

Receiver Operating Characteristic (ROC)
True Positive Rate
False Positive Rate
ROC curve (area = 0.82)
0.0
0.2
0.4
0.6
0.8
1.0

4.6. LightGBM

4.6.1. SMOTE

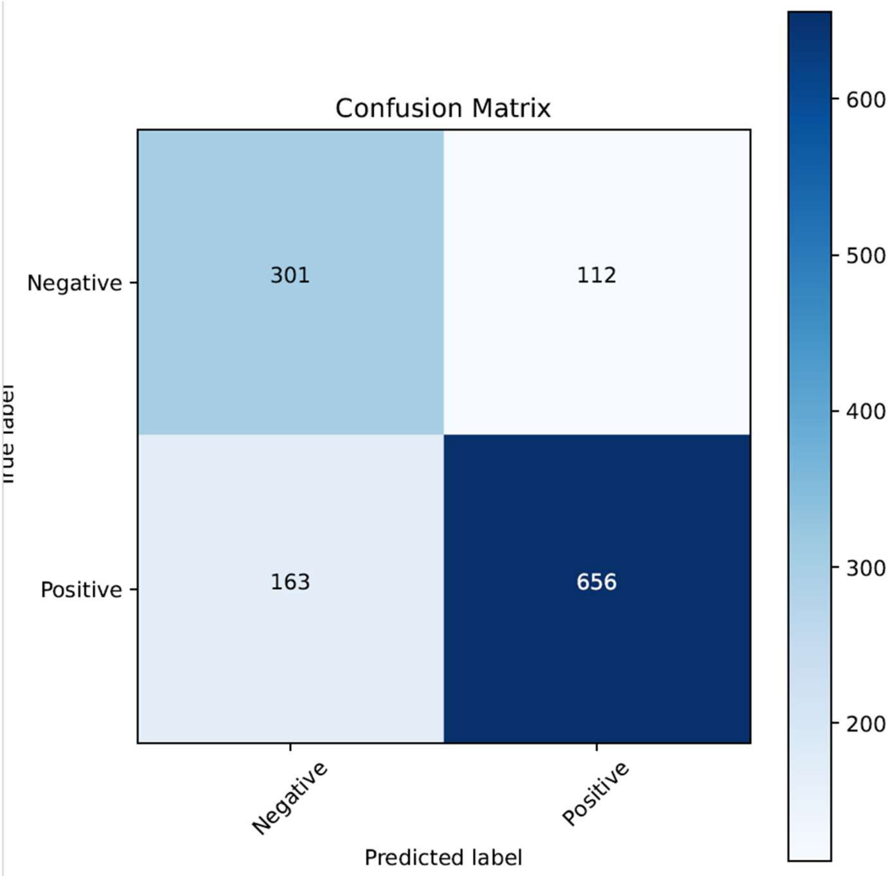

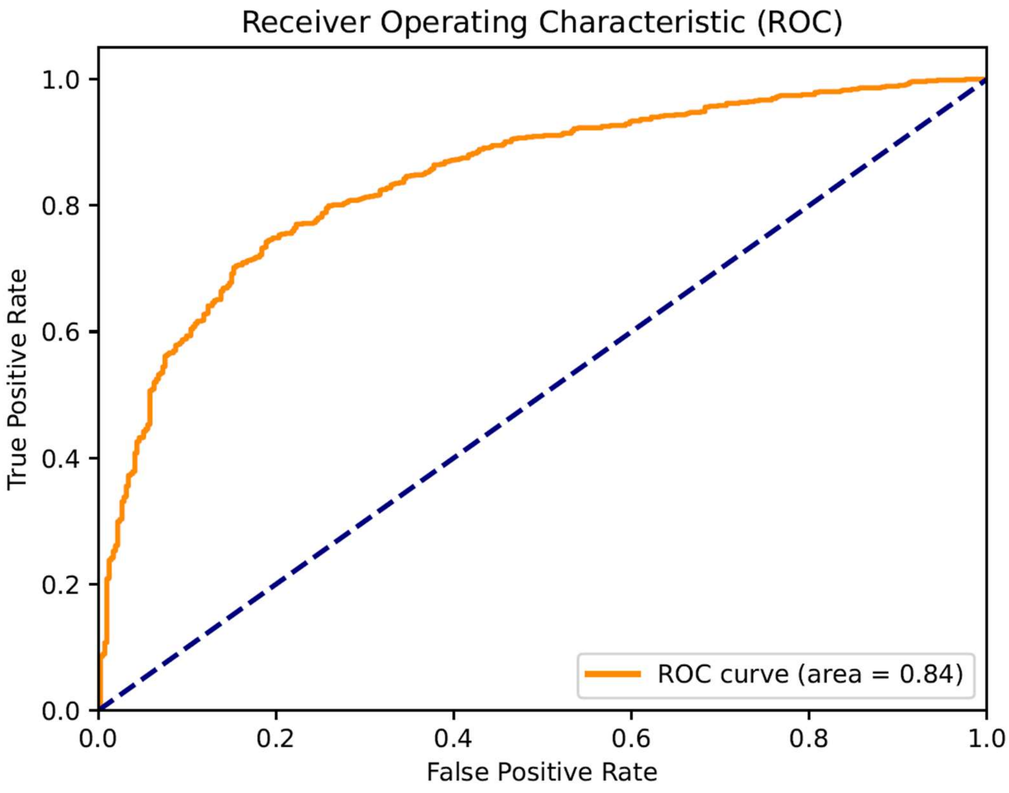

4.6.2. ADASYN

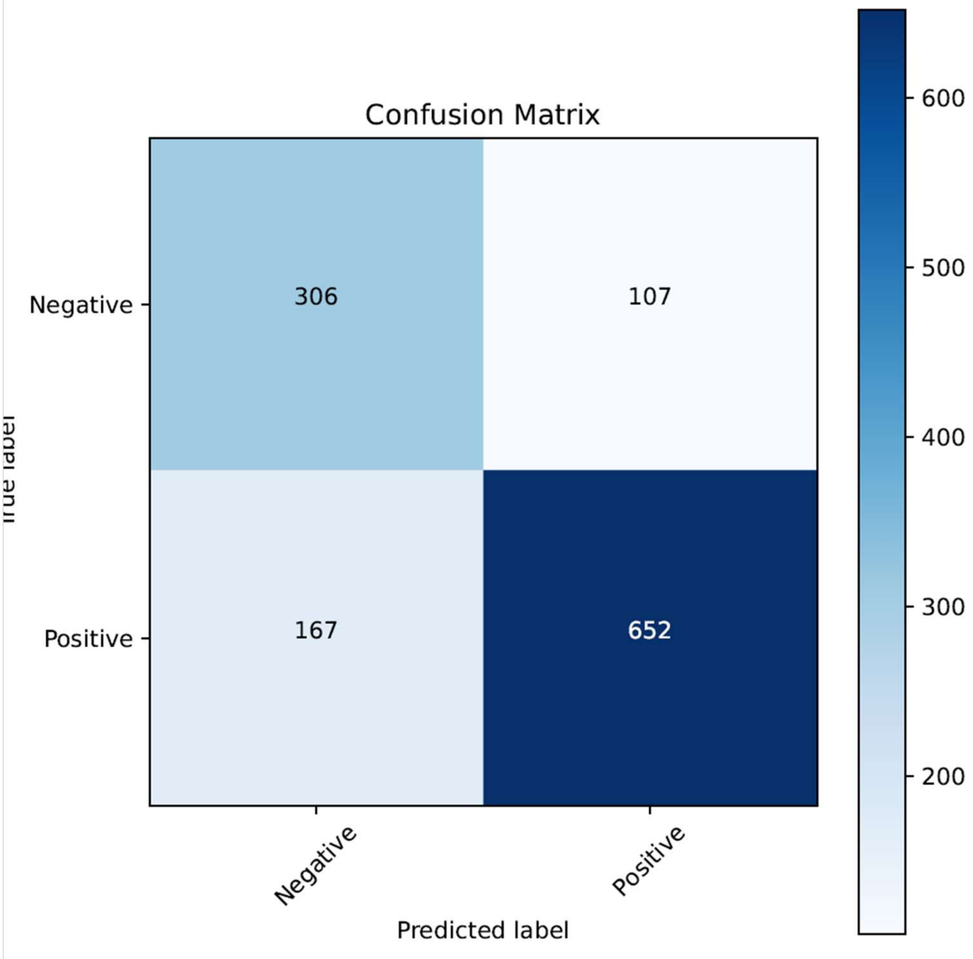

Confusion Matrix
Negative
Positive
True label
306
107
167
652
Negative
Positive
Predicted label
600
500
400
300
200

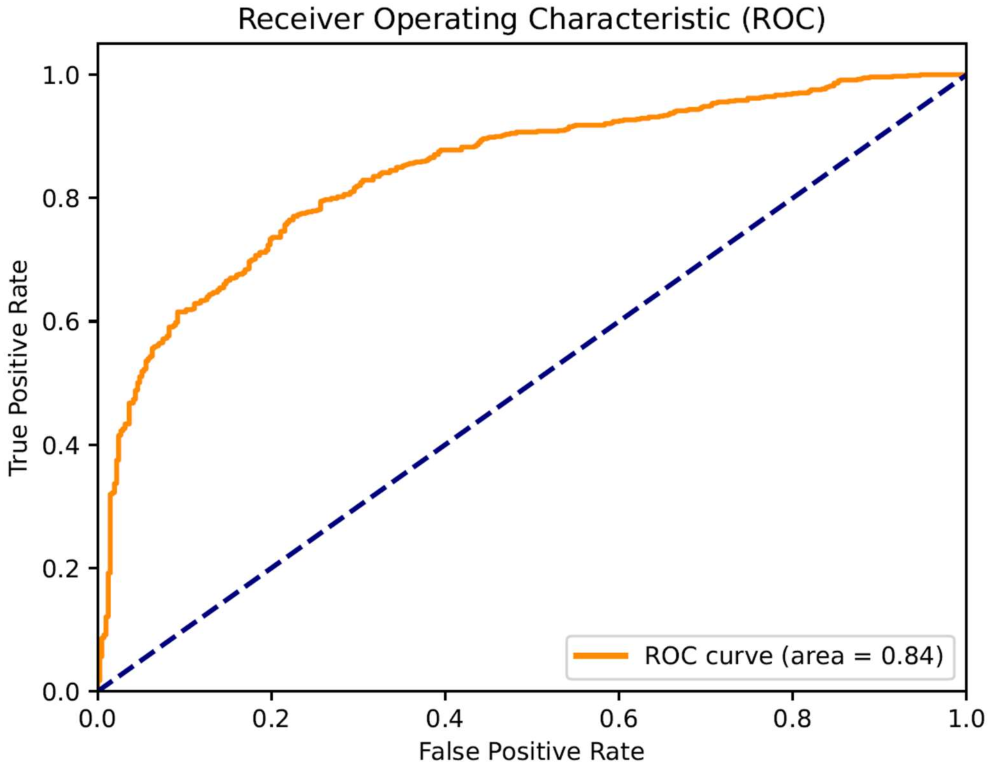

Receiver Operating Characteristic (ROC)
True Positive Rate
False Positive Rate
0.0
0.2
0.4
0.6
0.8
1.0
ROC curve (area = 0.84)

4.7. Logistic Regression

4.7.1. SMOTE

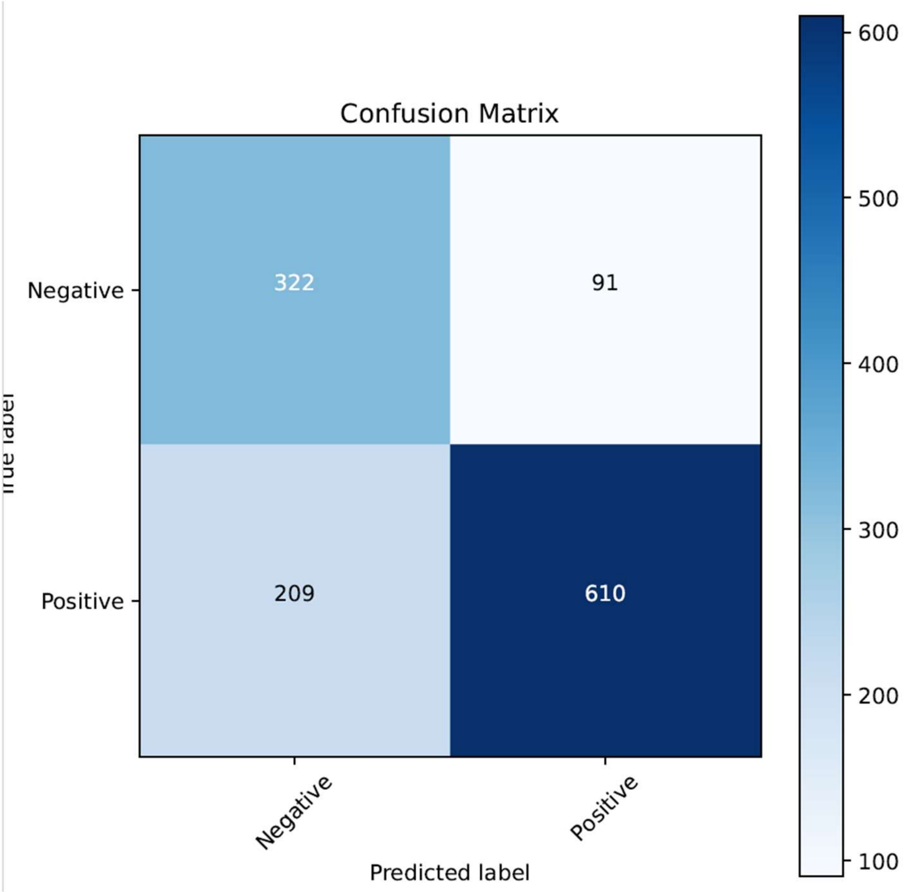

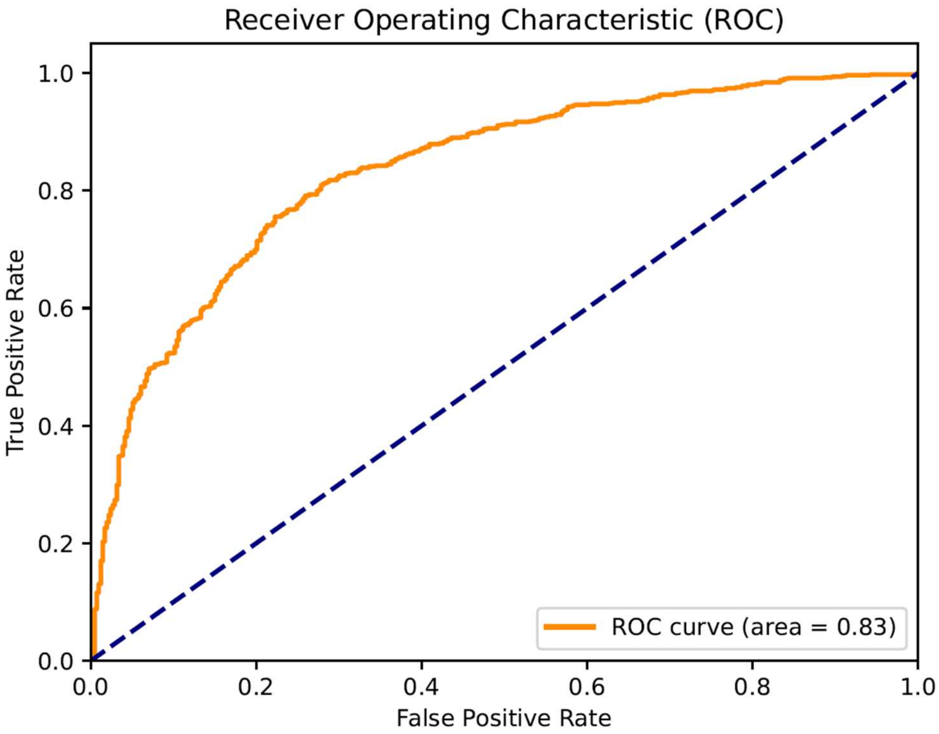

4.7.2. ADASYN

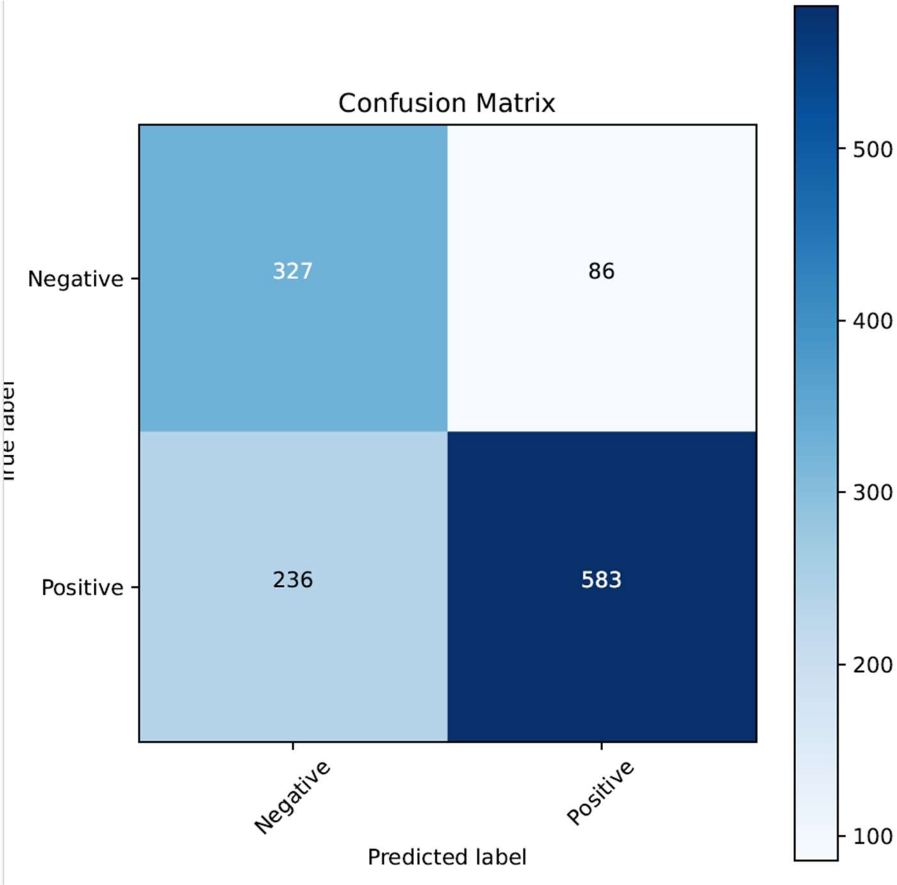

Confusion Matrix
327
86
236
583
Negative
Positive
True label
Negative
Positive
Predicted label
500
400
300
200
100

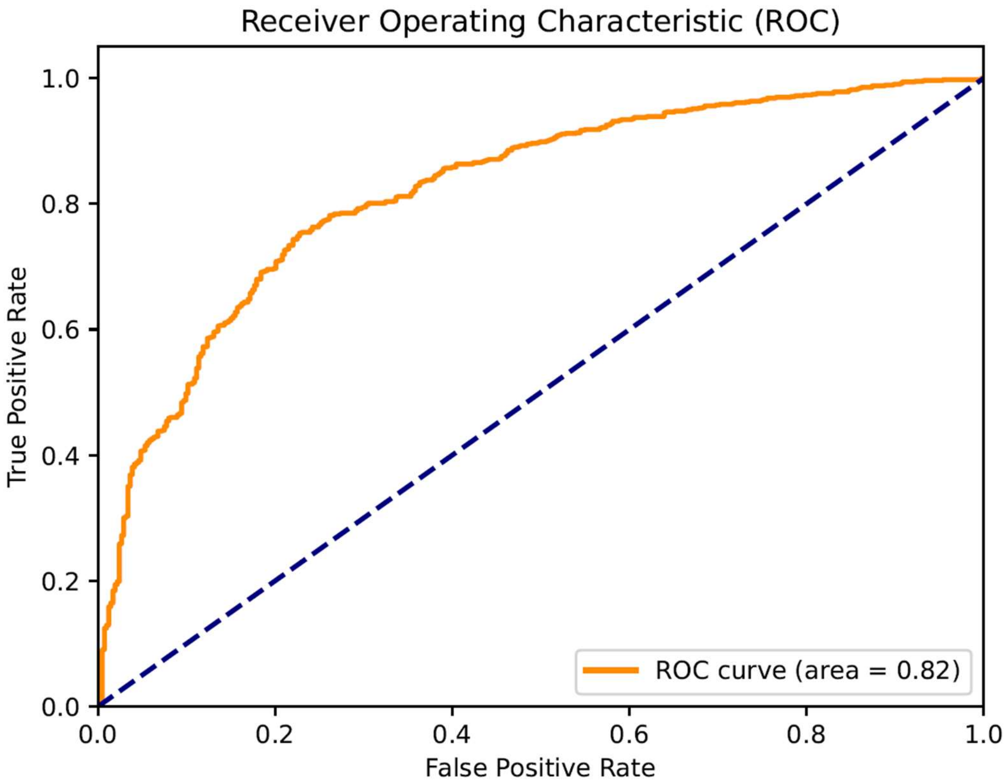

Receiver Operating Characteristic (ROC)
True Positive Rate
False Positive Rate
0.0
0.2
0.4
0.6
0.8
1.0
ROC curve (area = 0.82)

4.8. NuSVM

4.8.1. SMOTE

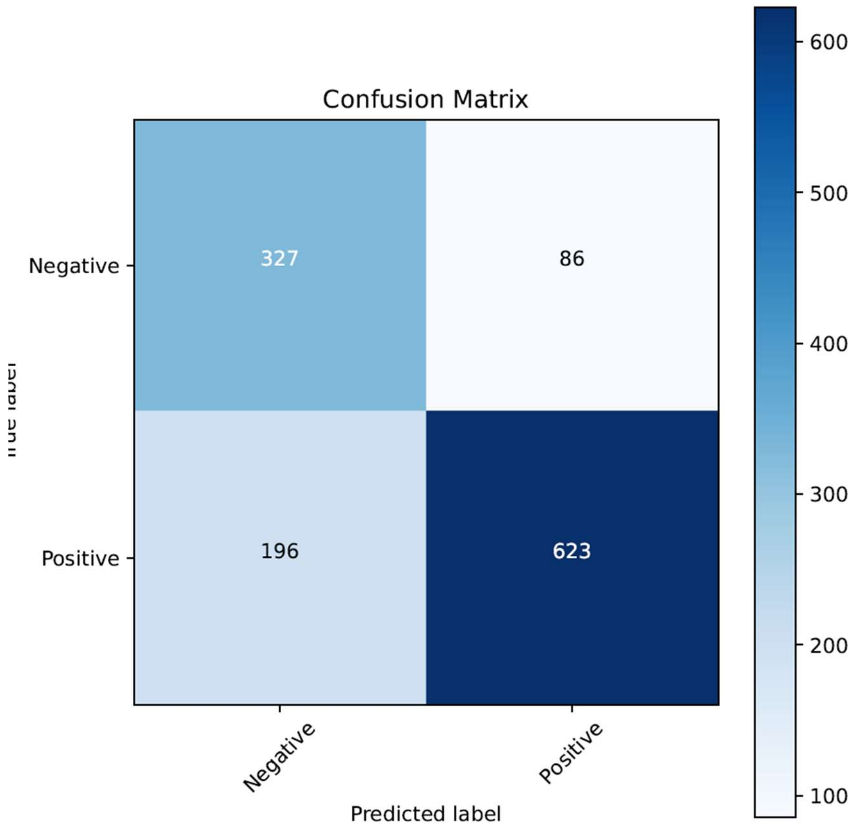

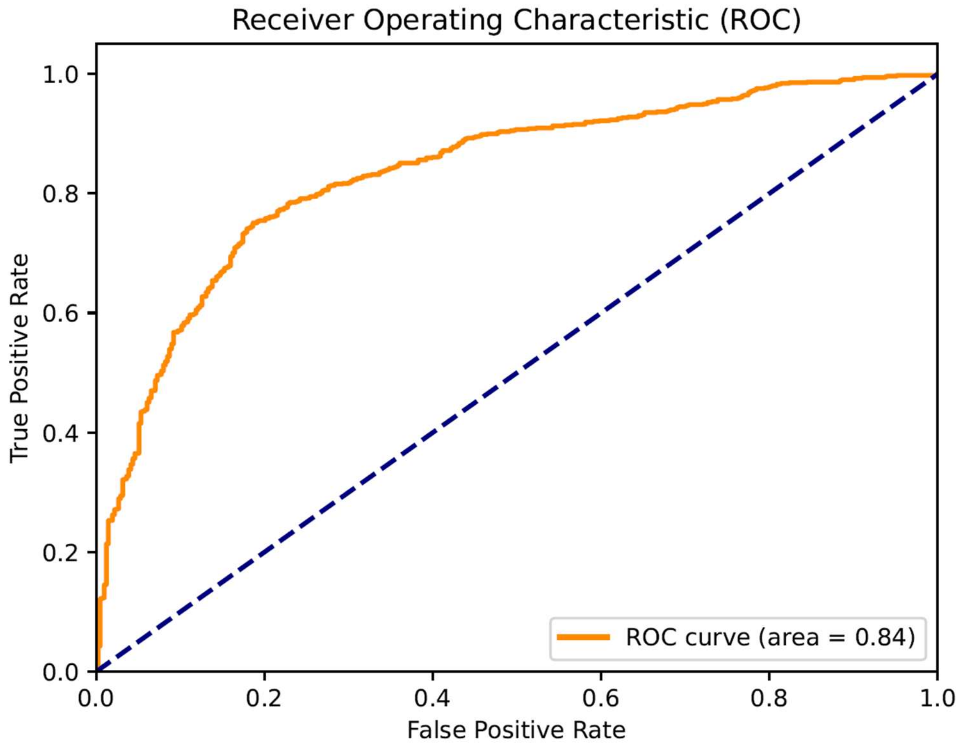

4.8.2. ADASYN

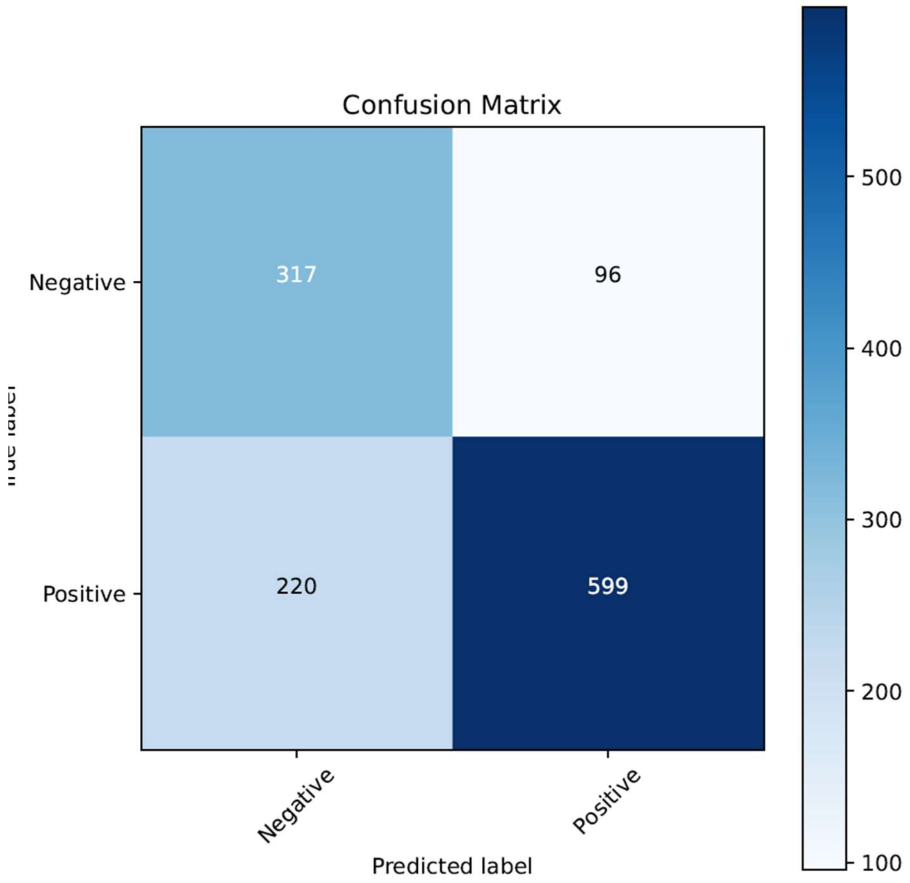

Confusion Matrix
Negative
Positive
317
96
220
599
Negative
Positive
Predicted label
500
400
300
200
100

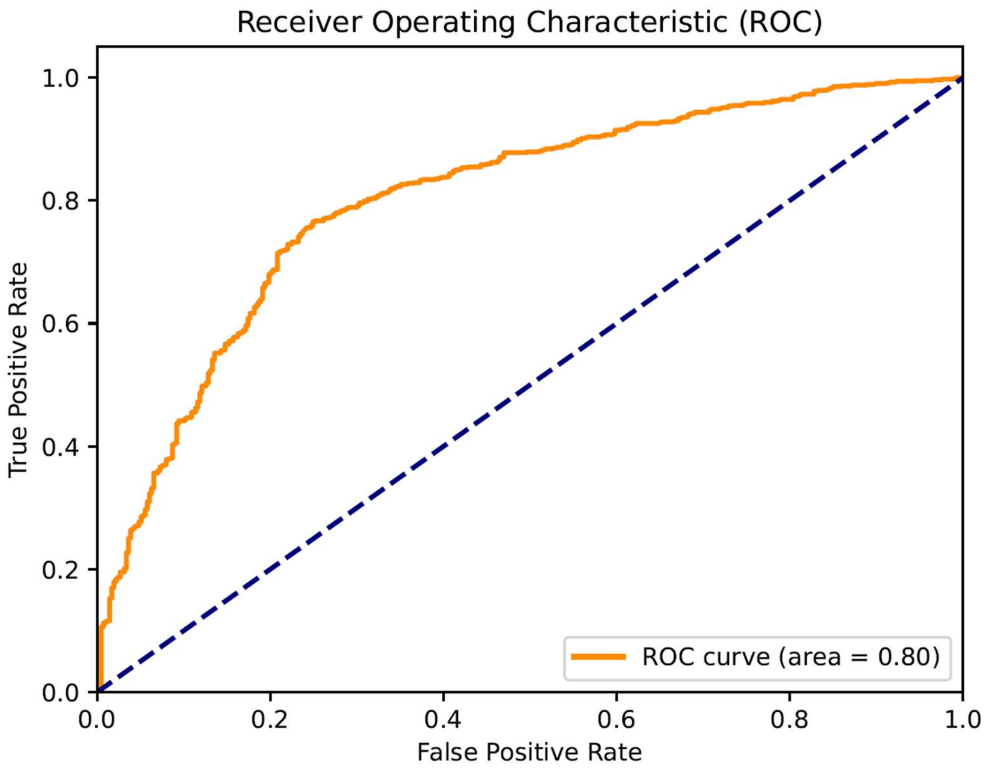

Receiver Operating Characteristic (ROC)
True Positive Rate
False Positive Rate
0.0
0.2
0.4
0.6
0.8
1.0
ROC curve (area = 0.80)

4.9. Random Forest

4.9.1. SMOTE

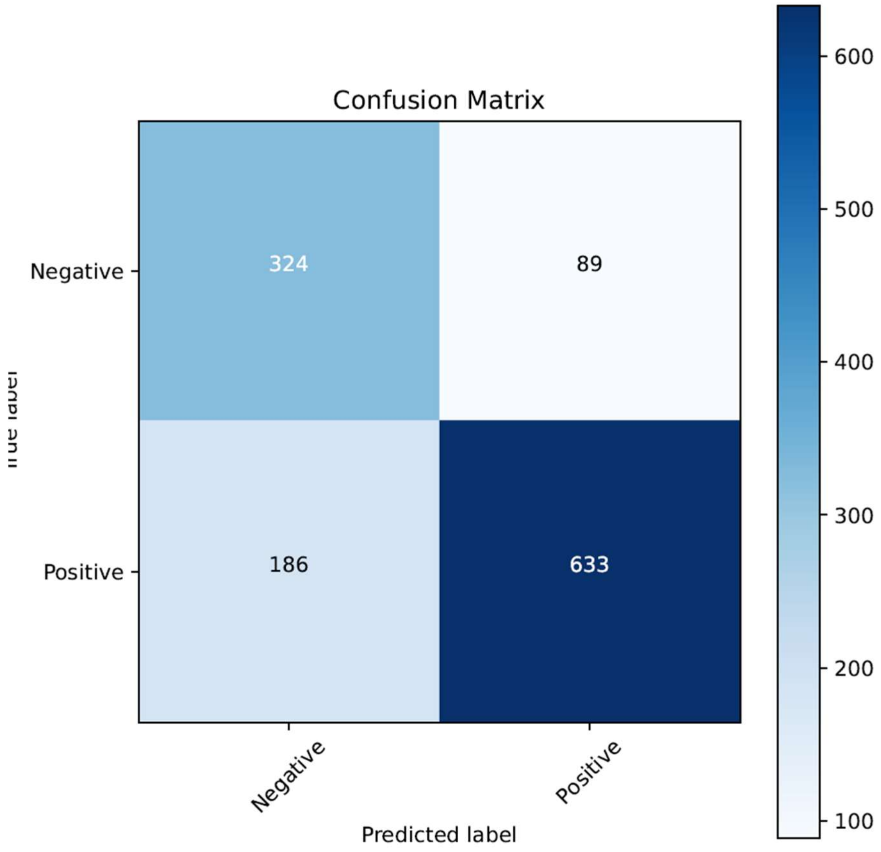

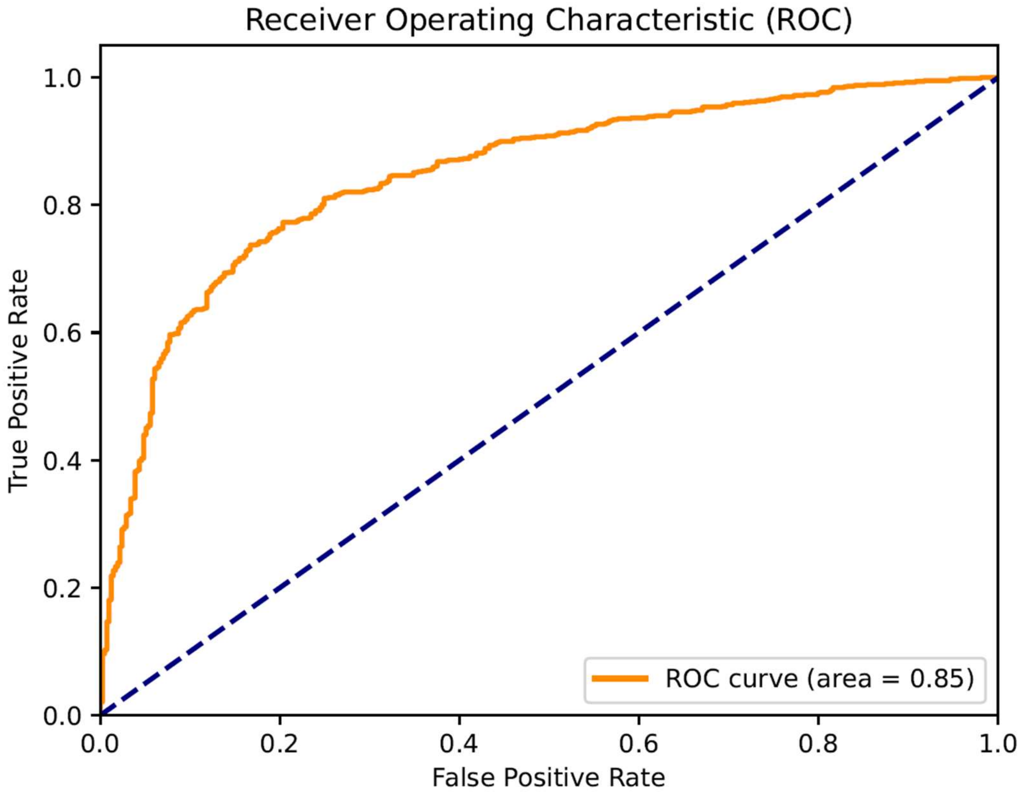

4.9.2. ADASYN

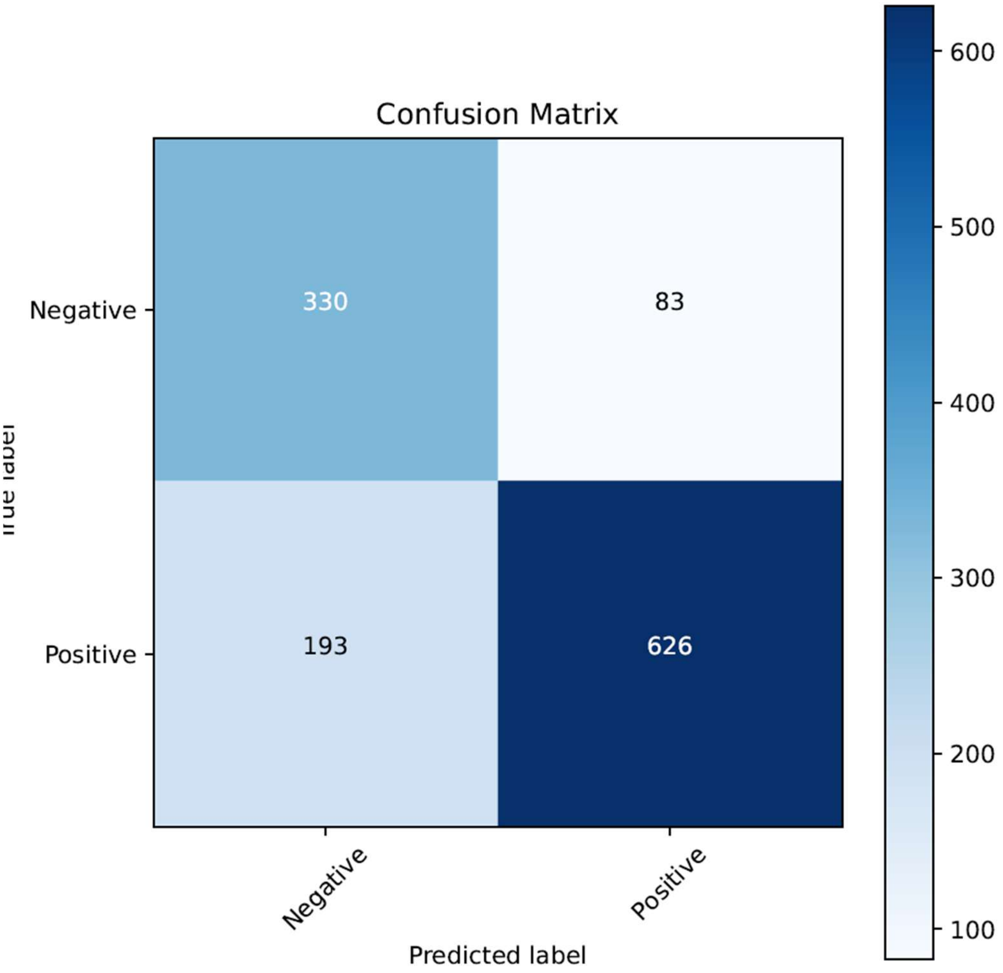

Confusion Matrix
Negative
Positive
330
83
193
626
Negative
Positive
Predicted label
600
500
400
300
200
100

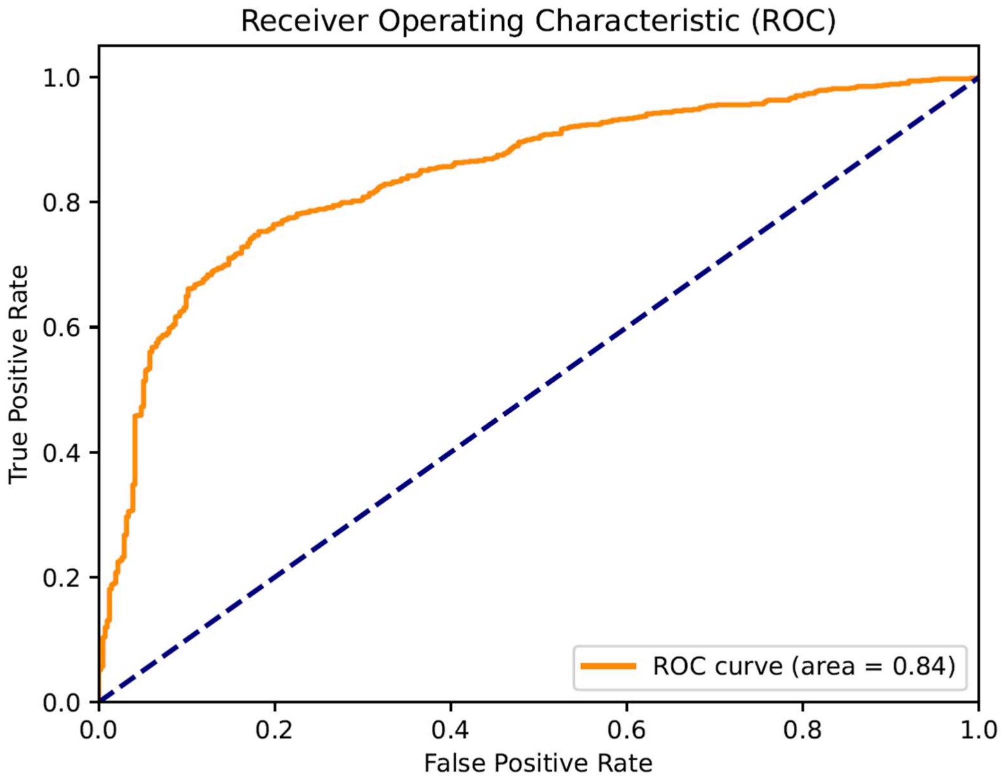

Receiver Operating Characteristic (ROC)
True Positive Rate
False Positive Rate
0.0
0.2
0.4
0.6
0.8
1.0
0.0
0.2
0.4
0.6
0.8
1.0
ROC curve (area = 0.84)

4.10. XgBoost

4.10.1. SMOTE

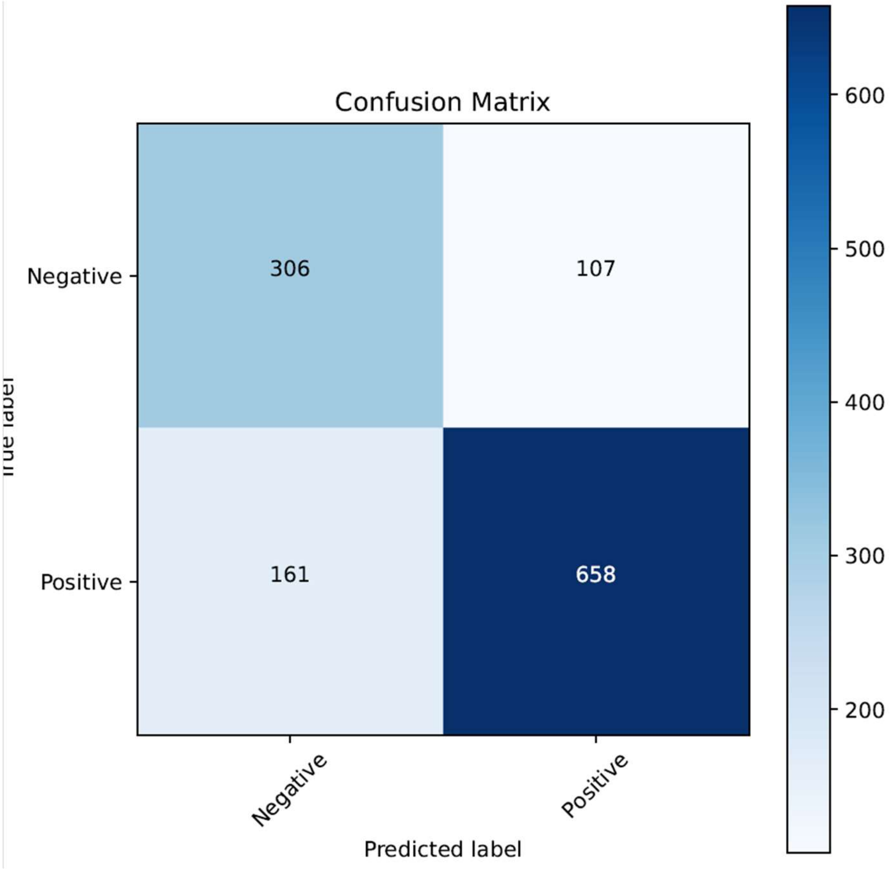

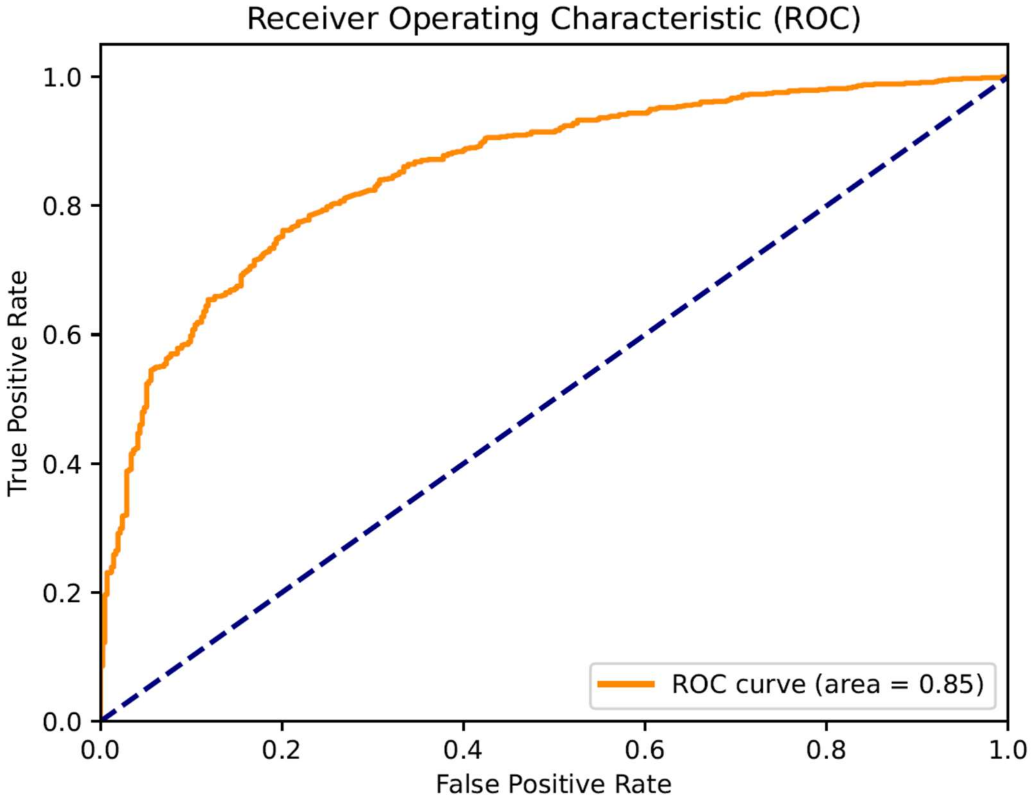

4.10.2. ADASYN

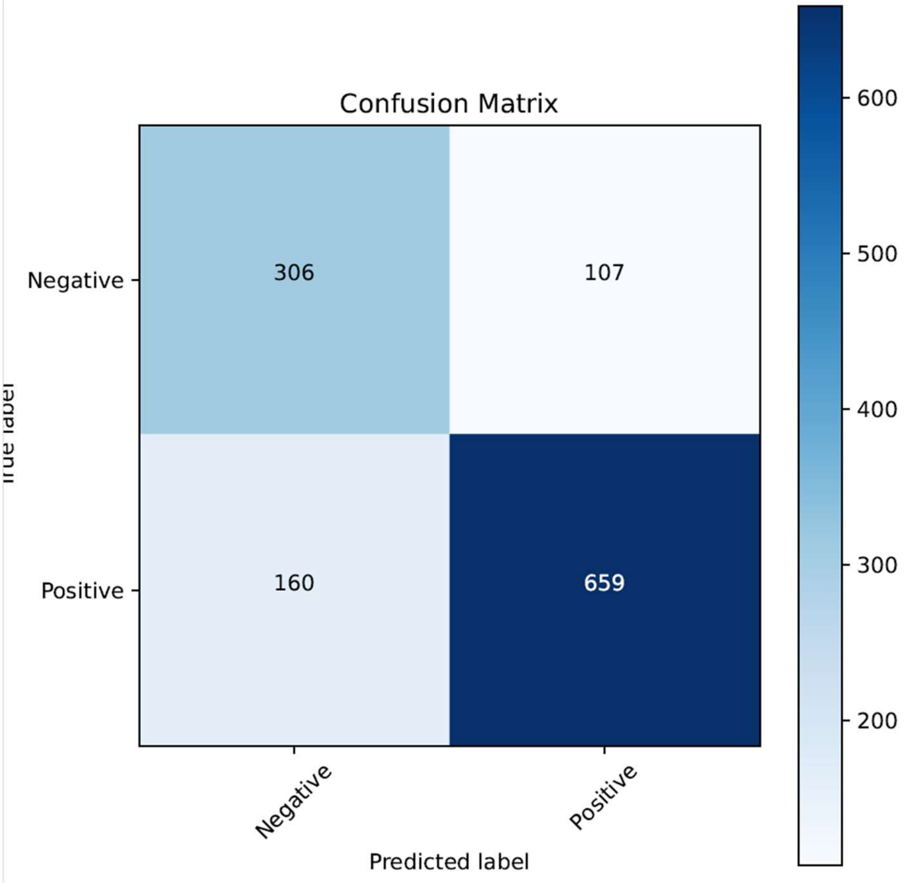

Confusion Matrix
Negative
Positive
306
107
160
659
True label
Predicted label
600
500
400
300
200

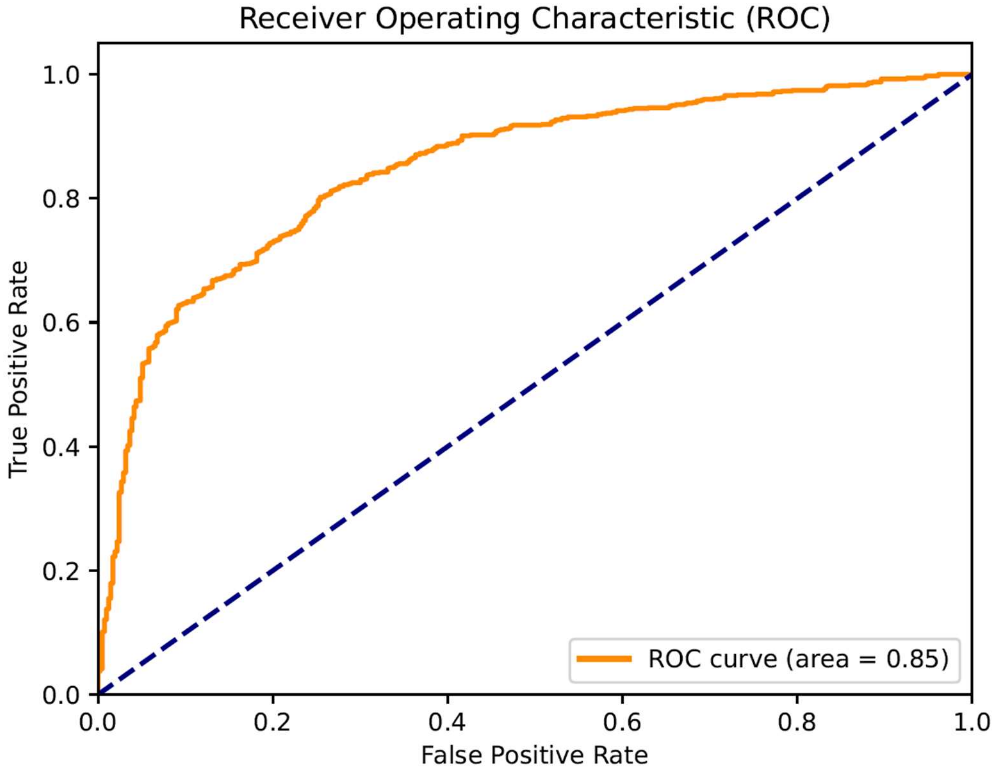

Receiver Operating Characteristic (ROC)
True Positive Rate
False Positive Rate
ROC curve (area = 0.85)
0.0
0.2
0.4
0.6
0.8
1.0

5. Ranking, Absolute numbers, Incidence, Both sexes, in 2022 for thyroid cancer.

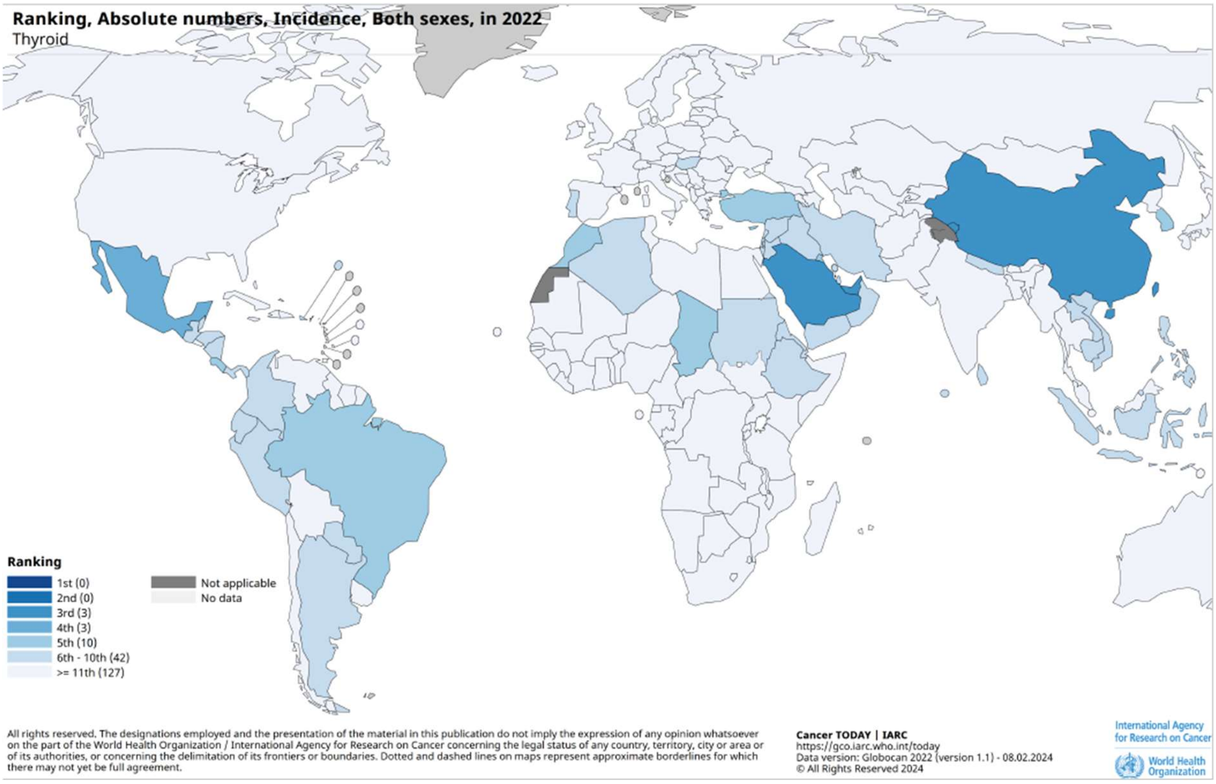

6. Optimized parameters of individual classifiers

6.1. Decision tree

**Parameter Name**	**Allowed Values**
criterion	"gini", "entropy", "log_loss"
splitter	"best", "random"
max_depth	Integer (from 1 to 20)
min_samples_split	Integer (from 2 to 10)
min_samples_leaf	Integer (from 1 to 10)
min_weight_fraction_leaf	Floating point value (from 0.0 to 0.5)
max_leaf_nodes	Integer (from 2 to 20)
min_impurity_decrease	Floating point value (from 0 to 1)
ccp_alpha	Floating point value (from 0 to 1)

6.2. Extra Tree

Parameter Name	Allowed Values
criterion	"gini", "entropy", "log_loss"
splitter	"best", "random"
max_depth	Integer (from 1 to 20)
min_samples_split	Integer (from 2 to 10)
min_samples_leaf	Integer (from 1 to 10)
min_weight_fraction_leaf	Floating point value (from 0.0 to 0.5)
max_leaf_nodes	Integer (from 2 to 20)
min_impurity_decrease	Floating point value (from 0 to 1)
ccp_alpha	Floating point value (from 0 to 1)

6.3. Extra Trees

Parameter Name	Allowed Values
criterion	"gini", "entropy", "log_loss"
n_estimators	Integer (from 1 to 100)
max_depth	Integer (from 2 to 20)
min_samples_split	Integer (from 2 to 10)
min_samples_leaf	Integer (from 1 to 10)
min_weight_fraction_leaf	Floating point value (from 0.0 to 0.5)
max_leaf_nodes	Integer (from 2 to 20)
min_impurity_decrease	Floating point value (from 0 to 1)
ccp_alpha	Floating point value (from 0 to 1)
bootstrap	Boolean (True or False)
max_samples	Floating point value (from 0.01 to 1)

6.4. Gradient Boosting

Parameter Name	Allowed Values
max_depth	Integer (from 1 to 30)
loss	"log_loss", "exponential"
learning_rate	Floating point value (from 0 to 0.99)
subsample	Floating point value (from 0.01 to 1)
min_samples_split	Floating point value (from 0.01 to 1)
min_samples_leaf	Floating point value (from 0.01 to 1)
min_weight_fraction_leaf	Floating point value (from 0.01 to 1)
max_leaf_nodes	Integer (from 1 to 20)
min_impurity_decrease	Floating point value (from 0.01 to 0.5)
ccp_alpha	Floating point value (from 0 to 1)
criterion	"friedman_mse", "squared_error"
n_estimators	Integer (from 1 to 100)

6.5. KNN

Parameter Name	Allowed Values
metric	"euclidean", "manhattan", "chebyshev"
weights	"uniform", "distance"
n_neighbors	Integer (from 1 to 10)

6.6. LightGBM

Parameter Name	Allowed Values
max_depth	Integer (1 to 50)
min_child_weight	Integer (1 to 20)
learning_rate	Float (1e-8 to 0.99)
gamma	Float (0.01 to 2)
subsample	Float (0.2 to 1)
colsample_bytree	Float (0.2 to 1)
eval_metric	"rmse", "rmsle", "mae", "logloss", "map", "gamma-deviance", "aft-nloglik"
reg_alpha	Float (1e-8 to 1)
reg_lambda	Float (1e-8 to 1)
num_leaves	Integer (2 to 50)
min_child_samples	Integer (1 to 50)
bagging_freq	Integer (1 to 7)
feature_fraction	Float (0.1 to 1)
bagging_fraction	Float (0.1 to 1)
n_estimators	Integer (1 to 100)

6.7. Logistic Regression

Parameter Name	Allowed Values
C	Float (0.001 to 1000)
max_iter	Integer (1 to 2000)
penalty	"l1", "l2", "elasticnet", "none"
dual	Boolean (True or False)
fit_intercept	Boolean (True or False)
solver	"newton-cg", "lbfgs", "liblinear", "sag", "saga"
l1_ratio	Float (0 to 1, applicable only if penalty="elasticnet")

6.8. NuSVM

Parameter Name	Allowed Values
kernel	"linear", "rbf", "poly", "sigmoid"
nu	Float (0.001 to 0.5)

degree	Integer (1 to 5, applicable only if kernel="poly")
gamma	Float (0.001 to 0.01, applicable for kernels "rbf", "poly", "sigmoid")

6.9. Random Forest

**Parameter Name**	**Allowed Values**
criterion	"gini", "entropy", "log_loss"
n_estimators	Integer (1 to 100)
max_depth	Integer (1 to 20)
min_samples_split	Integer (2 to 10)
min_samples_leaf	Integer (1 to 10)
min_weight_fraction_leaf	Float (0.0 to 0.5)
max_leaf_nodes	Integer (2 to 20)
min_impurity_decrease	Float (0.0 to 1.0)
ccp_alpha	Float (0.01 to 1.0)
bootstrap	Boolean (True or False)
max_samples	Float (0.01 to 1.0)

6.10. XgBoost

**Parameter Name**	**Allowed Values**
max_depth	Integer (1 to 300)
min_child_weight	Float (1 to 20)
learning_rate	Float (1e-8 to 0.99)
gamma	Float (0.01 to 2)
subsample	Float (0.2 to 1)
colsample_bytree	Float (0.2 to 1)
eval_metric	"rmse", "rmsle", "mae", "logloss", "map", "gamma-deviance", "aft-nloglik"
n_estimators	Integer (1 to 100)
reg_alpha	Float (1e-8 to 1)
reg_lambda	Float (1e-8 to 1)
max_depth	Integer (1 to 300)
min_child_weight	Float (1 to 20)
learning_rate	Float (1e-8 to 0.99)
gamma	Float (0.01 to 2)
subsample	Float (0.2 to 1)
